# Rational Design and Evaluation of Novel TGR5 Agonists for Diabetes

**DOI:** 10.3390/molecules31071093

**Published:** 2026-03-26

**Authors:** Rachana S. Bhimanwar, Zachary Detwiler, Jinge G. Zhu, Samuel T. Saghafi, Carolyn A. Winder, Dawn Belt Davis, Amit Mittal, Vikas Sharma, David A. Harris, Snehal N. Chaudhari

**Affiliations:** 1 Department of Pharmaceutical Chemistry, Dr. D. Y. Patil Institute of Pharmaceutical Sciences and Research, Pune 411018, India; rachana.bhimanwar@dypvp.edu.in; 2 Department of Pharmaceutical Chemistry, School of Pharmacy and Research, Dnyaan Prasad Global University, Pune 411018, India; 3 Department of Pharmaceutical Chemistry, School of Pharmaceutical Sciences, Lovely Professional University, Phagwara 144411, India; amitmittal77@yahoo.com (A.M.); vikaspharma26@gmail.com (V.S.); 4 Wisconsin Institute for Discovery, University of Wisconsin-Madison, Madison, WI 53706, USA; zdetwiler@wisc.edu; 5 Wisconsin Surgical Laboratory in Metabolism (WiSLiM), Department of Surgery, University of Wisconsin-Madison, Madison, WI 53705, USA; jgzhu@wisc.edu (J.G.Z.); cwinder@wisc.edu (C.A.W.); 6 Division of Endocrinology, Diabetes, and Metabolism, Department of Medicine, University of Wisconsin-Madison, Madison, WI 53705, USA; ssaghafi@medicine.wisc.edu (S.T.S.); dbd@medicine.wisc.edu (D.B.D.); 7 Geriatrics Research Education and Clinical Center, William S. Middleton Memorial Veterans Hospital, Madison, WI 53705, USA; 8 William S. Middleton Memorial Veterans Hospital, Madison, WI 53705, USA; 9 Department of Biochemistry, University of Wisconsin-Madison, Madison, WI 53706, USA

**Keywords:** TGR5, diabetes, T2D, small molecules, GLP-1, G protein bias

## Abstract

Agonists of the G protein-coupled receptor TGR5 have long been sought-after for their metabolic benefits. Intestinal TGR5 activation induces secretion of the antidiabetic hormone GLP-1, which can systemically improve diabetes phenotypes in multiple organs. However, no TGR5 agonist drug candidate has succeeded in clinical trials due to their low potency and unwanted side effects. A challenge in the field has been the development of TGR5 agonists that are non-toxic, long-acting, and have functional selectivity for G protein-biased agonism. In this study, we propose a systematic pipeline for engineering optimal TGR5 agonists with antidiabetic properties. This pipeline is interdisciplinary, combining in silico, in vitro, and in vivo assays to design and validate drug candidates. We identify 2 lead compounds that outline the necessary beneficial properties for a successful TGR5 agonist against diabetes. We uncover the molecular mechanisms that allow TGR5 agonists to induce the transcription of their target, TGR5, in intestinal enteroendocrine cells. Lastly, we investigate the molecular interactions of our lead candidates in the TGR5 binding pocket to identify optimal parameters for stability and biological activity. Our strategy for TGR5 agonist design and evaluation has the potential to guide the discovery process for targeted TGR5 therapeutics for metabolic diseases.

## 1. Introduction

**Figure 1:**
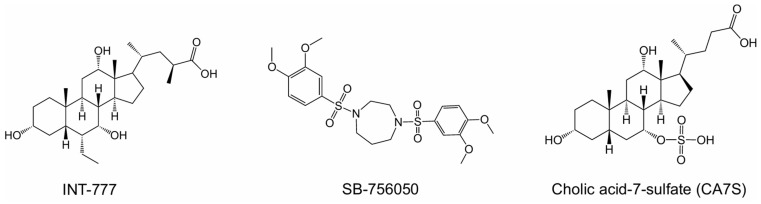
Examples of TGR5 agonists. Structures of INT-777, SB-756050, and CA7S, agonists of TGR5 investigated for their anti-diabetes effects.

Type 2 diabetes (T2D) is a global health threat. The pathogenesis of T2D is multifactorial, involving a complex interplay of genetic, environmental, and lifestyle factors [1]. If left untreated, T2D can lead to severe complications affecting multiple organ systems. Agonists of the Takeda G protein-coupled receptor (TGR5) are known to be potent antidiabetic drugs that work primarily through the induction of the antidiabetic hormone, Glucagon-Like Peptide 1 (GLP-1) [2,3]. Specifically, agonism of TGR5 on intestinal enteroendocrine cells can induce secretion of GLP-1 systemically, which engages target tissues via the GLP-1 receptor to improve T2D phenotypes [4]. Furthermore, TGR5 agonism can improve various T2D-related complications and comorbidities across multiple organs [5,6]. However, despite the beneficial impacts upon metabolic disease, clinical advancement of TGR5 agonists has been stymied by unintended side effects [7,8]. For example, INT-777, one of the earliest potent and selective TGR5 agonists, causes smooth-muscle relaxation, with negative consequences to the cardiac and biliary systems (Figure 1) [9].

Extensive research over the last 20 years has focused on improving TGR5 agonist design strategies to minimize undesirable off-target effects. SB-756050, a recent lead drug candidate and selective TGR5 agonist for T2D, showed good tolerability and no significant safety issues when evaluated in clinical trials (Figure 1) [10]. However, SB-756050 demonstrated highly variable pharmacodynamics, leading to its discontinuation from the drug development pipeline. The reasons for the unpredictable antidiabetic response to SB-756050 are unknown. However, it is suggested that inadequate interaction with TGR5 in the gut led to variable efficacy in reducing systemic glucose levels [10].

While GPCRs have historically been the most successfully targeted receptor class, their complex signal transduction pathways can cause difficulties for drug development. TGR5 activation, in a classic Gαs-coupled receptor activation pathway, can activate adenylyl cyclase (AC), resulting in an increase of cAMP and subsequently GLP-1 secretion from intestinal enteroendocrine cells [11]. Concomitantly, TGR5 activation can induce the recruitment of β-arrestins, resulting in internalization of the cell surface receptor and subsequent desensitization to agonists [12]. Therefore, new research has suggested that precision pharmacology strategies should focus on the development of biased receptor activators that enhance cAMP production with minimal to no arrestin pathway activation [11]. Moreover, activators that increase the expression or recycling of their target would be desirable. We have previously demonstrated the capabilities of specific sulfated bile acid derivatives to agonize TGR5 and induce its expression, thereby enhancing their antidiabetic properties (cholic acid-7-sulfate, Figure 1) [3].

**Figure 2:**
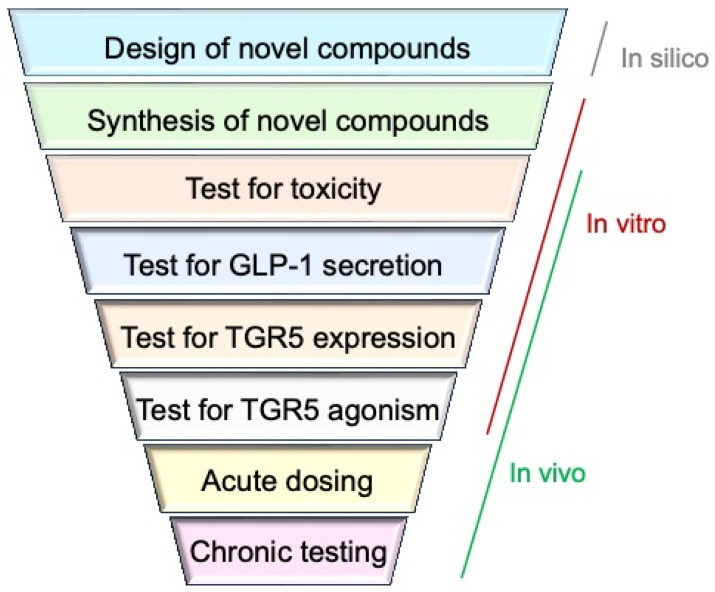
Systematic pipeline for evaluation of TGR5 agonists. In silico, in vitro, and in vivo assays can narrow down the most effective TGR5 agonists for T2D.

In this study, we designed and evaluated novel TGR5 agonists with potent TGR5 activating and expression-inducing capabilities. We provide a systematic pipeline for assessment of TGR5 agonists for safety, potency, and antidiabetic properties using a combination of in silico, in vitro, and in vivo assays (Figure 2). Furthermore, we evaluate chemical modalities in lead compounds that show G protein-biased agonism without engaging β-arrestins to propose synthesis strategies that can improve the pharmacodynamics of TGR5 agonists for T2D.

## 2. Results

### 2.1. Chemistry and Design Rationale for TGR5 Agonists

**Figure 3:**
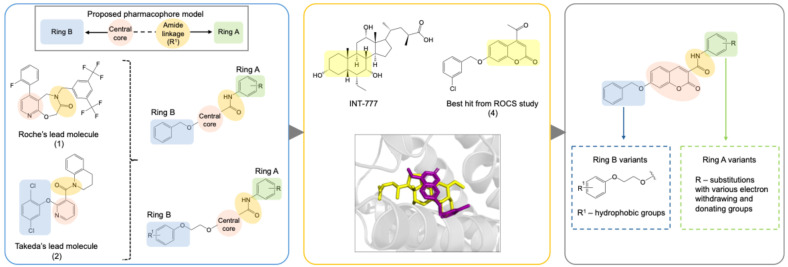
Design strategy for TGR5 agonists.

We analyzed the structures of potent synthetic TGR5 agonists developed by Roche (1) and the Takeda group (2) (Figure 3). We found structural similarities between these compounds; specifically, in distinctive features within the pyridine ring as the monocyclic hydrophobic central core moiety. The pyridine ring is connected to a heteroaromatic ring through an amide linkage and a subsequent phenyl ring linked directly or via an oxygen atom to the opposite end of this central core moiety [13,14]. Thus, our TGR5 agonist design scheme included a central core aromatic ring linked to a phenyl ring via amide linkage (Ring A) and phenoxy substitution to the other end (Ring B) (Figure 3).

The designed pharmacophore was divided into three fragments for the purpose of further exploration: the aryl amide moiety (Ring A), the central core, and the phenoxy moiety (Ring B). To initiate the identification of the central core, the observation was made that Compounds **1** and **2** possess the central monocyclic ring containing one or two heteroatoms. In previous studies, structural investigations primarily concentrated on Ring A and Ring B, with limited exploration of the central core [15]. In this study, we explored the modifications in the central core with the hypothesis that replacing the pyridine of the scaffold from Compounds **1** and **2** with bi-cyclic rings was likely to yield a new class of TGR5 agonists with equivalent or higher potency.

To test this hypothesis, in our previous study [16], iterative application of ligand and structure-based virtual screening using a rapid overlay of 3D chemical structures (ROCS v3.2.2) tool of OpenEye Scientific Software. INT-777 was used as a query to screen the ZINC database, which was used to identify novel TGR5 agonists for diabetes management. As a result of TanimotoCombo and Electrostatic Potential Similarity, the coumarin scaffold was selected for designing of molecules. Even the binding mode analysis of compound 4 with TGR5 was investigated and compared with INT-777. We found that the central heterocycle (coumarin) of compound 4 aligns with the steroid core moiety of INT-777 (Figure 1 and Figure 3). Therefore, we selected the coumarin ring as a central core for the discovery of new derivatives. It has been shown in previous studies that the binding pocket in TGR5 is predominantly hydrophobic [17,18]. Considering this and the binding analysis of INT-777 with TGR5, we hypothesized that compounds with more hydrophobic rings would increase the binding affinity for TGR5. Thus, we hypothesized that a 2-carbon chain linker between Ring B and the central core would fit suitably in the narrow channel while providing conformational flexibility, positional adaptability, and a nonpolar structure with no significant separation of charge. Modifications on the phenyl ring (Ring A) linked by an amide group were undertaken by substituting electron-withdrawing and electron-donating groups to elucidate the effect of substitutions on the properties of designed compounds.

**Figure 4:**
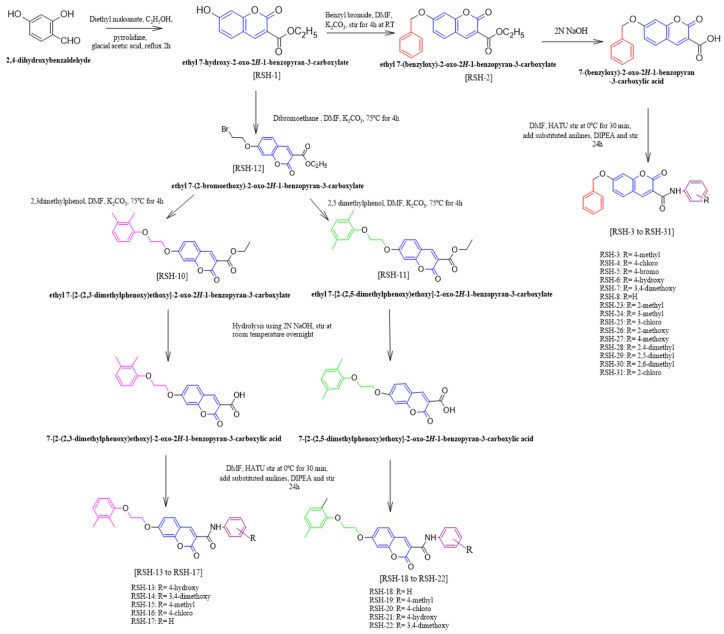
Synthetic pathway for designed coumarin derivatives.

The synthetic scheme of the key compounds is depicted in Figure 4. The synthesis of the intermediate RSH-2 involved a Knoevenagel condensation of 2,4-dihydroxybenzaldehyde and Diethylmalonate to yield RSH-1, followed by a Williamson ether synthesis via an SN2 mechanism [19]. In this step, compound RSH-1 was reacted with benzyl bromide in the presence of potassium carbonate using N,N-dimethylformamide (DMF) as a polar solvent, to form the key intermediate RSH-2 [20]. Following this, the ester derivative RSH-2 was hydrolyzed to a carboxylic acid derivative by dissolving it in ethanol and adding 2N NaOH [21]. Finally, coupling with diverse anilines afforded the corresponding amide derivatives RSH-3-RSH-8 and RSH-23-RSH-31 in moderate to excellent yields in the presence of coupling reagent HATU and DIPEA [22].

Furthermore, the synthesis of intermediate RSH-12 follows the principles of Williamson etherification, reaction with dibromoethane in the presence of potassium carbonate. The intermediates RSH-10 and RSH-11 were synthesized by reacting RSH-12 with 2,5-dimethylphenol and 2,3-dimethylphenol in the presence of a base at a temperature of 75 °C in dimethylformamide (DMF) [21]. Following the formation of ester derivatives 10 and 11, compounds were subjected to hydrolysis to yield carboxylic acid derivatives. Ultimately, reacting with diverse anilines led to the formation of corresponding amide derivatives, specifically RSH-13 to RSH-22 (NMR data, Supplementary Materials) [22].

### 2.2. In Vitro Biological Activities

**Figure 5:**
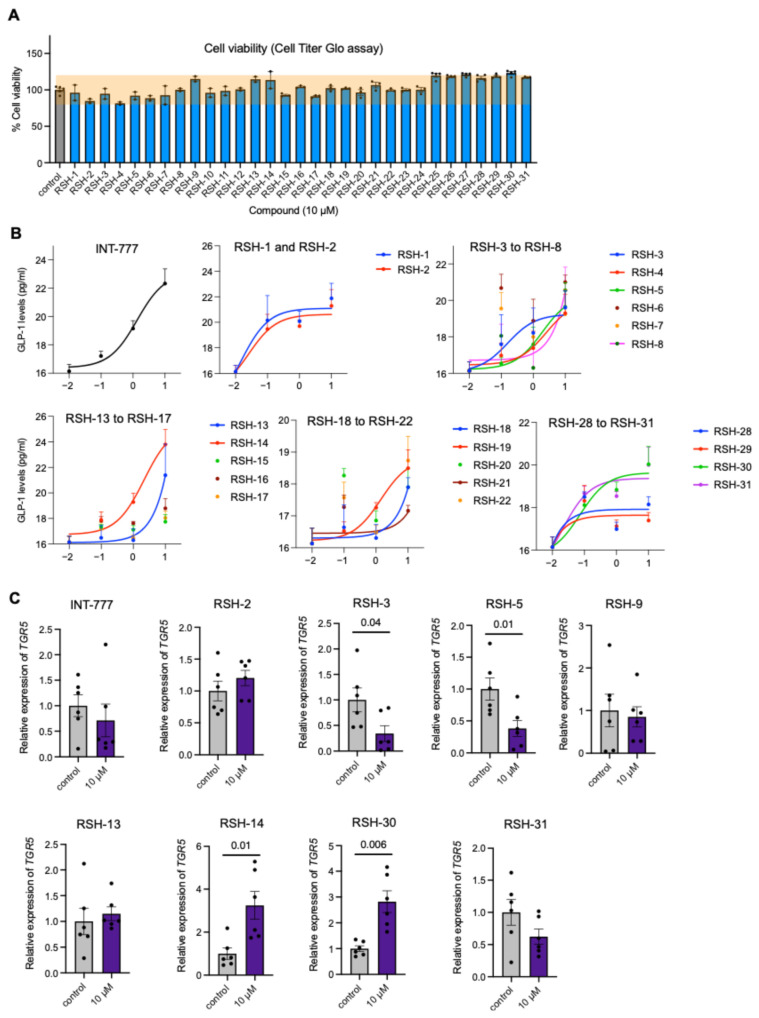
In vitro evaluation of *TGR5* agonists for safety, GLP-1 secretion, and *TGR5* expression. (**A**) RSH-1 to RSH-31 compounds tested for cell viability in NCI-H716 cells compared to DMSO control showed no overt toxicity at a 10 µM concentration. (**B**) Dose-response curves for GLP-1 secretion induced by the indicated compounds in NCI-H716 cells (EC_50_ in Table 1). (**C**) *TGR5* expression was measured by the indicated compounds. RSH-14 and RSH-30 are the only *TGR5* agonists that also induced their expression in NCI-H716 cells.

We next evaluated compounds in vitro (Figure 2). Compounds were preliminarily screened for toxicity in human enteroendocrine NCI-H716 cells incubated overnight at a 10 µM concentration (Figure 5A). NCI-H716 cells were chosen because they are intestinal L cells that are used to screen for compounds that trigger TGR5 for secretion of GLP-1 [3]. No compound appeared to have overt toxicity that affected viability >20% of the DMSO control. Next, we tested the ability of all compounds to induce GLP-1 secretion in NCI-H716 cells in a dose-dependent manner. We determined EC_50_ values for compounds and compared them to INT-777 (Figure 5B, Table 1). We shortlisted 10 compounds with EC_50_ values that were <2.6, which is within 2-fold of the EC_50_ for INT-777 (Table 1, highlighted).

Shortlisted compounds were next tested for their effectiveness in inducing the expression of TGR5, a property that could potentially prolong drug signaling. NCI-H716 cells were incubated with 10 µM of compounds overnight and were tested the next day for TGR5 expression by qPCR. While many studies have evaluated the TGR5 activation and GLP-1 secretion capability of INT-777, none have tested its impact on TGR5 expression. At 10 µM concentration, we observed no induction of TGR5 expression by INT-777 (Figure 5C). Among the shortlisted TGR5 agonists, RSH-14 and RSH-30 showed a ~3-fold increase in TGR5, while other compounds showed either no change or a decrease in TGR5 expression (Figure 5C). Therefore, we selected RSH-14 and RSH-30 for further evaluation.

**Figure 6:**
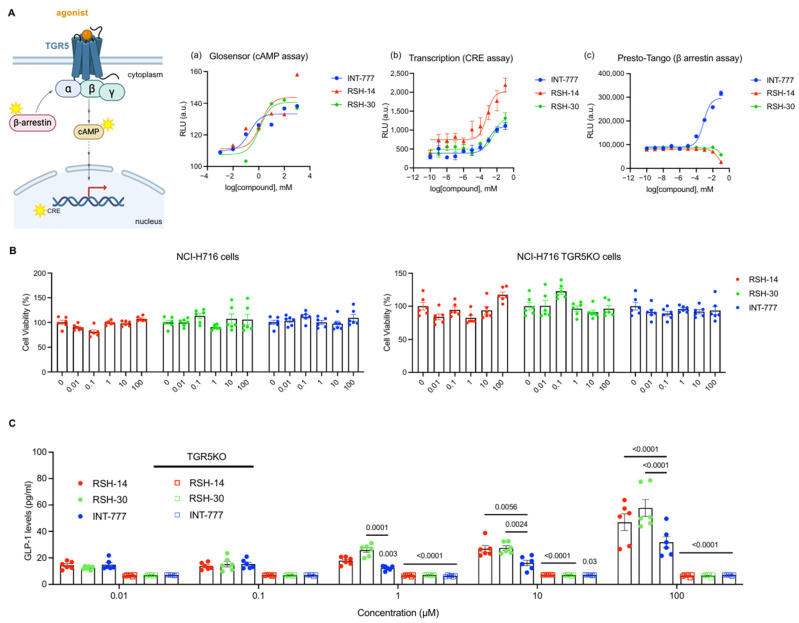
Evaluation of TGR5 agonists for G protein-biased agonism and induction of GLP-1 secretion. (**A**) (left) Cartoon of GPCR activation. (right) Evaluation of indicated TGR5 agonists for G protein-biased agonism as measured by cAMP activation (a), CRE DNA binding (b), and β-arrestin recruitment assays (c). (**B**) RSH-14, RSH-30, and INT-777 compounds tested for cell viability in NCI-H716 WT (top) and TGR5KO (bottom) cells compared to DMSO control showed no overt toxicity at the indicated range of concentrations. (**C**) RSH-14 and RSH-30 induce GLP-1 at a higher capacity than INT-777 in NCI-H716 cells. GLP-1 secretion is abolished in TGR5KO cells.

With our lead compounds RSH-14 and RSH-30, we next evaluated their ability to induce G protein-biased agonism. To test this, we performed luciferase-based measurements of cAMP activation, cAMP-response element DNA binding, and recruitment of β-arrestin, the 3 canonical signaling cascades induced by TGR5 activation [12] (Figure 6A). First, using the GloSensor assay [23], we found that RSH-14, RSH-30, and INT-777 induced a robust increase in cAMP activation. Next, by transfecting a cAMP response element (CRE)-driven luciferase reporter construct in NCI-H716 cells, we measured nuclear transduction of TGR5 activation [3]. RSH-14 activated TGR5 in a dose-dependent manner and was superior to RSH-30 and INT-777, which similarly activated TGR5. However, when testing for β-arrestin recruitment by compounds using HTLA cells transfected with human TGR5 [24], we found that INT-777 showed a dose-dependent increase in activity, while RSH-14 and RSH-30 were inactive in this assay. This suggests that while INT-777 induces activation and internalization of the TGR5 receptor, RSH-14 and RSH-30 maintain high TGR5-induced cytoplasmic and nuclear activation without reducing cell surface expression. Together, this suggests that RSH-14 and RSH-30 show G protein-biased agonism compared to INT-777.

We expanded the dose-response curves in the GLP-1 secretion assay to include concentrations up to 100 µM of compounds, which exceeds the concentration exposed to the intestinal epithelium when mice are administered a maximum dose of 100 mg/kg of agonists. At 1, 10, and 100 µM doses, no compounds showed toxicity (Figure 6B). RSH-14 and RSH-30 performed better than INT-777 in inducing GLP-1 secretion (Figure 6C). RSH-14 and RSH-30 showed ~1.5-fold and ~2-fold higher GLP-1 secretion at 100 µM concentration, respectively, compared to INT-777. Lastly, to test the importance of TGR5 for compounds to induce GLP-1, we generated a CRISPR-based knockout (KO) of TGR5 in NCI-H716 cells. The GLP-1-inducing ability of RSH-14, RSH-30, and INT-777 was significantly impaired in NCI-H716 TGR5KO cells, demonstrating the necessity of this GPCR in promoting secretion of the antidiabetic hormone GLP-1 by these compounds (Figure 6B,C).

### 2.3. In Vivo Evaluation

**Figure 7:**
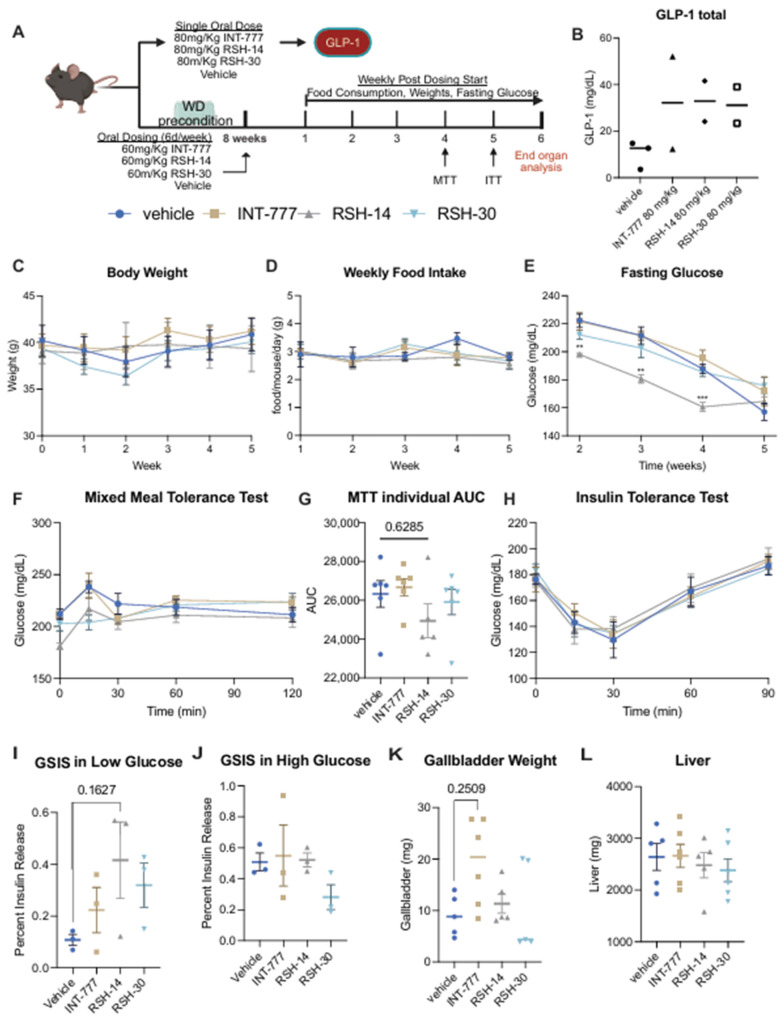
In vivo and ex vivo assessment of compound efficacy. (**A**) Experimental Design (**B**) Circulating GLP-1 after a single dose of compound. Data represent mean (*n* = 3 in control, *n* = 2 for compounds). (**C**–**E**) Weekly body weight, food intake, and fasting blood glucose. ** *p* < 0.001; *** *p* < 0.0001. Unpaired T-test with Welch correction comparing RSH-14 to Vehicle. Multiple comparisons corrected for via Holm-Sidak (*n* = 6 in control, RSH-30, and INT-777 group; *n* = 5 in RSH-14 group). (**F**,**G**) Mixed meal tolerance test with calculated area under the curve (*n* = 6 in control, RSH-30, and INT-777 group; *n* = 5 in RSH-14 group). (**H**) insulin tolerance test (*n* = 6 in RSH-30 and INT-777 group; *n* = 5 in Control and RSH-14 group). (**I**,**J**) Glucose-stimulated insulin secretion under low (basal; 2 mM) and high (feeding; 17 mM) glucose (*n* = 3 in each group). (**K**,**L**) Gallbladder and liver weights at euthanasia (*n* = 5 in control, RSH-30, and RSH-14; *n* = 6 INT-777 group); one control animal died prior to sac, and one gallbladder specimen from the RSH-30 group ruptured during dissection. (**G**,**I**–**L**) The Shapiro–Wilk test showed non-normal distribution. Kruskal–Wallis testing with Dunn’s test for multiple comparisons. All data represent mean ± SEM.

We next sought to investigate the acute (Figure 7A top) and chronic (Figure 7A bottom) impact of our novel TGR5 agonists on metabolism in vivo. First, in a proof-of-concept experiment, we found that a single oral dose of 80 mg/kg of INT-777, RSH-14, and RSH-30 led to a >2-fold increase in total GLP-1 as compared to vehicle (Figure 7B) in diet induce obese mice reared on a western diet (WD; 42% calories from fat; 30% *w*/*v* Sucrose).

Given that the target for these agents is patients with obesity and associated metabolic disease, we performed a head-to-head comparison in diet-induced obese mice. Male mice were reared on Western diet (WD) for 8 weeks to induce obesity and glucose intolerance. Mice were then randomized to receive daily (6 days per week) INT-777, RSH-14, RSH-30, or vehicle by oral gavage for a total of 6 weeks. This dosing strategy did not cause a significant weight change (Figure 7C) or food intake (Figure 7D). However, mice receiving RSH-14 had the lowest fasting glucose across the first 5 weeks of the study period (Figure 7E). After 4 weeks of dosing, RSH-14-treated animals appeared to have slightly improved glucose tolerance as measured by a mixed meal tolerance test (Figure 7F,G), which was largely driven by improved fasting glucose. This occurred in the absence of changes in insulin tolerance (Figure 7H). This suggests that RSH-14 is more potent at regulating fasting glucose levels as compared to our other lead candidate, RSH-30, and our positive control INT-777 in a chronic setting.

Given that there was no improvement in glucose disposal but an apparent improvement in fasting glucose, we next sought to evaluate insulin secretion. Explanted islets from mice treated with compounds were exposed to low and high glucose conditions. Interestingly, in keeping with our in vivo results, RSH-14-treated animals had improved glucose-stimulated insulin secretion (GSIS) under basal conditions (Figure 7I) but not under feeding-like conditions (Figure 7J). Importantly, RSH-14 displayed the strongest fasting GSIS, with a >400% increase in secreted insulin, as compared to RSH-30 (~350%) and INT-777 (~100%). Overall, these results suggest that RSH-14 can improve fasting hyperglycemia in a Western diet model of diabetes.

Importantly, we found that RSH-14 and RSH-30-treated mice did not have an increase in gallbladder weights, which we found in INT-777 mice (Figure 7K). Lastly, there was no change in liver weight (Figure 7L) across our groups. In conclusion, our systematic pipeline for design and evaluation of TGR5 agonists produced a novel compound, RSH-14, that can induce sustained TGR5 agonism and GLP-1 secretion in vitro and in vivo, without deleterious side effects.

### 2.4. In Vitro and In Silico Evaluation of G Protein-Biased Agonism

**Figure 8:**
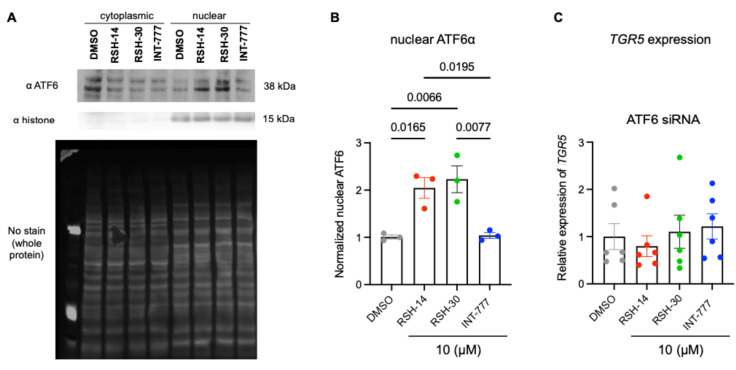
ATF6α is required for TGR5 agonists to induce receptor expression. (**A**,**B**) Western blot analysis of NCI-H716 cells when treated with the indicated agonists and fractionated to separate nuclear and cytoplasmic fractions. RSH-14 and RSH-30 increase nuclear shuttling of ATF6α, while INT-777 does not, compared to DMSO control. Anti-histone antibody staining in only the nuclear fraction indicates successful fractionation of cells. Quantification of nuclear ATF6α from Western blot analyses. One-way ANOVA with Dunnett’s multiple comparison test. All data represent mean ± SEM. (**C**) Induction of *TGR5* expression by RSH-14 and RSH-30 is abolished when NCI-716 cells are treated with ATF-6α siRNA.

Given the superiority of RSH-14 and RSH-30 in vitro and in vivo in the induction of GLP-1 and maintenance of antidiabetic effects, we next wanted to investigate why these compounds increase expression of their target, TGR5. Previous studies have shown that the ATF6α transcription factor can induce expression of TGR5 in skeletal muscle cells [25]. To test whether agonists that induce TGR5 expression also activate ATF6α, we measured nuclear ATF6α levels in NCI-H716 cells incubated with 10 µM of compounds. Briefly, we performed subcellular fractionation of cells [26] to isolate nuclear and cytoplasmic components, followed by Western blot analysis for ATF6α and histone. Histone was only detected in the nuclear and not the cytoplasmic fraction, confirming successful subcellular fractionation (Figure 8A). Strikingly, we observed a 2-fold increase in nuclear ATF6α in RSH-14 and RSH-30 treated cells as compared to INT-777 (Figure 8A,B and Figure S49).

To determine whether ATF6α is required for RSH-14 and RSH-30 to induce TGR5 expression, we performed a siRNA-mediated knockdown (KD) of ATF6α in NCI-H716 cells, followed by an overnight incubation with 10 µM compounds. qPCR analysis for *TGR5* revealed no expression induction by RSH-14 and RSH-30, suggesting that ATF6α is required for TGR5 expression in these cells (Figure 8C). Thus, we identified 2 lead candidates that can activate the TGR5-GLP-1 axis with the capability of G protein-biased agonism and induction of TGR5 expression. Further research on the relationship between TGR5 agonism and its direct transcriptional regulation via ATF6α is warranted.

Finally, we performed docking analysis of our lead compounds RSH-14 and RSH-30 to identify putative interactions with the TGR5 binding pocket that would render G protein-biased agonism without engaging β-arrestins.

**Figure 9:**
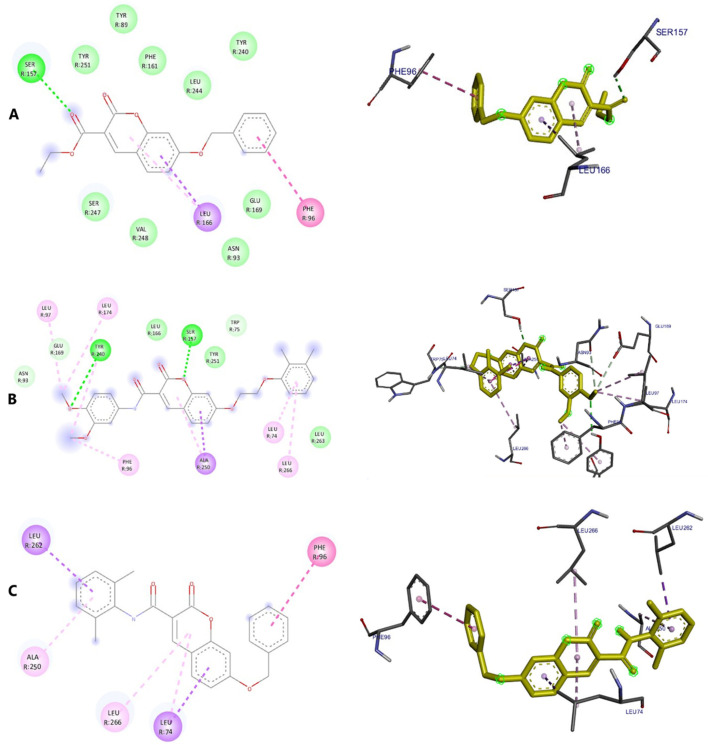
Binding affinities of compounds with TGR5 showing amino acid interactions in the binding pocket. 2D and 3D representations of binding affinities of RSH-2 (**A**), RSH-14 (**B**), and RSH-30 (**C**) with TGR5 (PDB ID: 7CFN).

First, we compared binding pocket interactions across our RSH compounds of RSH-14 and RSH-30 to RSH-2, a compound that showed low EC_50_ for GLP-1 secretion, but a decrease in TGR5 expression (Figure 5B,C). We observed that RSH-14 and RSH-30 had enhanced binding affinity and stability in the TGR5 receptor binding site as compared to RSH-2 (Figure 9). RSH-2 has a moderate binding affinity of −8.2 kcal/mol, and limited hydrophobic interaction range with comparatively lower efficacy (Figure 9A), whereas RSH-14 (−9.8 kcal/mol) and RSH-30 (−10.0 kcal/mol) showed better binding energies. RSH-14 forms several hydrogen bonds with Ser157 and Tyr240, as well as hydrophobic interactions with Ala250, Leu266, and Leu74, facilitated by its methoxy and coumarin ring (Figure 9B). In RSH-30, the greater binding preference is obtained primarily from hydrophobic and *π–π* interactions with important residues (Leu266, Leu74, and Phe96) (Figure 9C). Overall, the more favorable binding profiles of RSH-14 and RSH-30 suggest that they may be more stable and potentially more biologically active than RSH-2.

When we compare the structure–activity relationship analysis across our compounds, we see that TGR5 expression was strongly influenced by both the electronic properties and substitution pattern of Ring A and the steric and hydrophobic features of Ring B. An increase in TGR5 expression (approximately three-fold) was observed for compounds containing disubstituted electron-donating groups on Ring A, most notably the 3,4-dimethoxy (RSH-14) and 2,6-dimethoxy (RSH-30) analogues. This increase may be due to two electron-donating groups present on the benzene ring, which increase the stabilizing effect of aromatic interactions in the TGR5 binding pocket. Compounds such as RSH-7, RSH-22, and RSH-23, which have one electron-donating group on Ring A or one or more electron-withdrawing groups, failed to enhance TGR5 expression, suggesting that mono-substituted compounds do not provide sufficient interaction with the receptor.

The position of the electron-donating and withdrawing group appears to play an important role. Substitution of methyl and methoxy groups at the 3, 4- or 2, 6-positions favors alignment of compounds within the pocket of TGR5 by enhancing hydrophobic and π–π interactions. In addition to Ring A, substitutions on Ring B further influenced TGR5 expression. Hydrophobic substituents such as a benzyl group (RSH-30) or a 2, 3-dimethyl phenoxy moiety (RSH-14) provided further binding within the receptor. Overall, the results suggest that compounds that increase TGR5 expression have a synergistic balance between the electronic and conformational optimization of Ring A, along with sufficient hydrophobic interaction provided by Ring B. The structure–activity relationship (SAR) analysis of designed TGR5 agonists is shown in Table 2.

## 3. Materials and Methods

### 3.1. Synthetic Materials and Methods

All chemicals and reagents used in the synthesis process were acquired from Merck Ltd. (Mumbai, India), and S.D. Fine Chemicals Ltd. (Mumbai, India). Analytical grade pure solvents were used. The synthesis of compounds was carried out using a parallel synthesizer (Radley’s Carousel 6 Plus reaction station). The compounds synthesized were characterized using melting point, FT-IR, ^1^H NMR, ^13^C NMR, and mass spectroscopy (see Supplementary data). FT-IR spectra were recorded using a Fourier Transform InfraRed Spectrometer (8400S, Shimadzu Corporation, Kyoto, Japan). Bruker Avance III HD NMR 500 MHz (Bruker Corporation, Billerica, MA, USA) was used to record ^1^H NMR, i.e., proton nuclear magnetic spectra, and ^13^C NMR, i.e., carbon nuclear magnetic spectra, using CDCl_3_ as solvent. The Bruker Impact II UHR-TOF Mass Spectrometer System was used to obtain HR-MS. The melting point in degrees Celsius of test derivatives was determined using an Electronics Digital melting point apparatus.

#### 3.1.1. Synthesis of Ethyl 7-Hydroxy-2-oxo-2*H*-1-benzopyran-3-carboxylate (RSH-1)

2,4 dihydroxy benzaldehyde (2.0 mmol, 1-equiv) and Diethylmalonate (2.4 mmol, 1.2-equiv) were mixed in 2 mL of ethanol at room temperature. Subsequently, pyrrolidine 2–3 drops and glacial acetic acid were added in order, and the mixture was refluxed for two hours. Once the reaction was completed, the resulting product was poured into chilled water to obtain the product. The product was filtered, washed, and dried. The purified solid was obtained using column chromatography (hexane: ethyl acetate) [27,28]. Yellow solid; %Yield 85%; mp. 140–141 °C; *Rf*: 0.33 (7:13 EtOAc: hexane); IR (cm^−1^): 3500.92 (OH), 1724.42 (C=O Ester), 1600.97 (C=O lactone), 1496.30 (C=C), 1242.20 (C-C), 1026.16 1141.50 (C-O-C), ^1^H NMR (500 MHz, CDCl_3_) δ: 1.39–1.42 (t, 3H, CH_3_), 4.39–4.43 (q, 2H, CH_2_), 6.87–6.89 (d, *J* = 10 Hz, 1H, -C_5_H-chrom), 6.92 (s, 1H, C_8_H-chrom.), 7.50–7.51 (d, *J* = 5 Hz, 1H, -C_6_H-chrom), 8.53 (s, 1H, C_4_H-chrom.) (Figure S1), ^13^C NMR (500 MHz, CDCl_3_) δ: 165.55, 163.91, 157.78, 156.97, 149.74, 132.52, 114.91, 110.55, 102.33, 61.17, 24.11, 14.62.

#### 3.1.2. Synthesis of Ethyl 7-(Benzyloxy)-2-oxo-2*H*-1-benzopyran-3-carboxylate (RSH-2)

An appropriate quantity of ethyl 7-hydroxy-2-oxo-2*H*-chromene-3-carboxylate (0.236 g, 1.0 mmol) was dissolved in 2 mL of dimethylformamide (DMF) at room temperature. Subsequently, Benzyl bromide (0.171 mL, 1.2 mmol) was added. The potassium carbonate (0.151 g, 1.1 mmol) as a catalyst was added to the reaction mixture and stirred for 4 h at RT. Afterwards, the product was precipitated out using ice water and was filtered, washed, and dried. Using hexane: ethyl acetate as a solvent, column chromatography was performed to purify the crude solid. Yellow solid; %Yield: 91%; mp. 180–182 °C; *Rf*: 0.54 (7:13 EtOAc: hexane); IR (KBr) (cm^−1^): 1745.64 (C=O ester), 1610.61 (C=O lactone), 1392.65 (C=C), 1024.26 1120.68 (C-O-C); ^1^H NMR (500 MHz, DMSO) δ: 1.28–1.31 (t, 3H, CH_3_), 4.25–4.29 (q, 2H, CH_2_), 5.26 (s, 2H, OCH_2_Ar), 7.07–7.09 (d, *J* = 10 Hz, 1H, C_5_H-chrom.), 7.13 (s, 1H, C_8_H-chrom.), 7.35–7.37 (t, 1H, Ar), 7.40–7.43 (t, 2H, Ar), 7.47–7.49 (d, *J* = 10 Hz, 2H, Ar), 7.85–7.86 (d, *J* = 5 Hz, 1H, C_6_H-chrom.), 8.72 (s, 1H, C_4_H-chrom.) (Figure S2); ^13^C NMR (125 MHz, CDCl_3_) δ: 164.24, 163.29, 157.35, 156.71, 149.56, 136.45, 132.16, 129.03, 128.69, 128.47, 114.31, 113.99, 112.08, 101.67, 70.69, 61.41, 14.59; HR-MS (*m*/*z*):*m*/*z* calcd for [M+H]^+^ C_19_H_16_O_5_ 325.45; found 325.10.

#### 3.1.3. Synthesis of 7-(Benzyloxy)-2-oxo-2*H*-1-benzopyran-3-carboxylic Acid

An appropriate quantity of ethyl 7-(benzyloxy)-2-oxo-2*H*-chromene-3-carboxylate (2.0 mmol) was dissolved in ethanol as a solvent. A solution of 40 mL 2N NaOH was added, and the stirring of the solution was carried out overnight. Next, 2N HCl was added to neutralize the solution, which resulted in a precipitate that was filtered and washed twice with water. The resulted product was used as is for further reaction [29]. Pale Yellow solid; %Yield: 85%; mp. 184–185 °C; *Rf*: 0.45 (7:13 EtOAc: hexane); IR (cm^−1^): 3043.77 (C-H), 1716.70 (C=O acid), 1595.18 (C=O), 1408.03 (C=C), 1246.05 (C-C), 1132.25 (C-O-C).

#### 3.1.4. Synthesis of Compounds (RSH-3 to RSH-8 and RSH-23 to RSH-31)

7-(benzyloxy)-2-oxo-2*H*-1-benzopyran-3-carboxylic acid (1.0 mmol, 1-equiv) was dissolved in 4–5 mL of DMF previously kept in ice. Further Hexafluorophosphates Azabenzotriazole Tetramethyl Uronium (HATU) (0.72 mmol) was added to the solution and was stirred for 30 min. Next, corresponding substituted anilines (0.75 mmol) and N,N-Diisopropylethylamine (DIPEA) (1.4 mmol) were added and stirred overnight. This mixture was poured into chilled water, then extracted with ethyl acetate as a solvent, and the final product was obtained. After drying with magnesium sulfate, rotary evaporation was used to remove the ethyl layer. Through column, solvent system ethyl acetate: n-hexane was used for purification of crude product (RSH-3 to RSH-8 and RSH-23 to RSH-31) [29,30,31].

#### 3.1.5. 7-(Benzyloxy)-N-(4-methylphenyl)-2-oxo-2*H*-chromene-3-carboxamide (RSH-3)

Brown solid, Yield: 75%; mp: 220–222 °C; *Rf*: 0.80 (7:13 EtOAc: hexane); IR (cm^−1^): 3419.56 (N-H), 3033.82 (C-H), 1703.03 (C=O lactone), 1610.45 (C=O amide), 1508.23 (C=C), 1373.22 (C-N), 1253.64 (C-C), 1074.28 (C-O-C); ^1^H NMR (500 MHz, CDCl_3_) δ: 2.34 (s, 3H, CH), 5.19 (s, 2H, OCH_2_Ar), 6.96–6.97 (s, 1H, C_8_H-chrom), 7.03–7.05 (d, *J* = 10 Hz, 2H, C_5_H-chrom.), 7.17–7.18 (d, *J* = 5 Hz, 2H, Ar), 7.42–7.44 (m, 4H, Ar), 7.61–7.62 (d, 2H, C_6_H-chrom), 7.2–7.64 (d, 2H, Ar), 8.94 (s, 1H, C_4_H-chrom.), 10.71 (s, 1H, amide) (Figure S3); ^13^C NMR (125 MHz, CDCl_3_) δ: 164.07, 162.20, 159.73, 156.62, 148.65, 135.29, 134.29, 131.11, 129.54, 128.61, 128.87, 127.56, 120.50, 115.11, 114.84, 112.72, 101.9, 70.86, 20.95 (Figure S4); HR-MS (ESI): *m*/*z* calcd for [M+H]^+^ C_24_H_20_NO_4_ 386.42; found 386.1399 (Figure S5).

#### 3.1.6. 7-(Benzyloxy)-N-(4-chlorophenyl)-2-oxo-2*H*-chromene-3-carboxamide (RSH-4)

Pale brown solid, Yield: 67%; mp. 220–221 °C; *Rf*: 0.83 (7:13 EtOAc: hexane); IR (cm^−1^): 3267.19 (N-H), 1706.88 (C=O amide), 1610.45 (C=O), 1236.29 (C-C), 1010.63, 1091.63 (C-O-C); ^1^H NMR (500 MHz, CDCl_3_) δ: 5.19 (s, 2H, OCH_2_Ar), 6.97 (s, 1H, C_8_H-chrom), 7.04–7.06 (d, *J* = 10 Hz, 1H, C_5_H-chrom.), 7.32–7.34 (d, *J* = 10 Hz, 3H, Ar), 7.43–7.44 (d, *J* = 5 Hz, 4H, Ar), 7.63–7.65 (d, *J* = 10 Hz, 1H, C_6_H-chrom), 7.68–7.70 (d, *J* = 10 Hz, 2H, Ar), 8.93 (s, 1H, C_4_H-chrom.), 10.83 (s, 1H, amide) (Figure S6); HR-MS (ESI): *m*/*z* calcd for [M+H]^+^ C_23_H_17_NO_4_Cl 406.84; found 406.0841 (Figure S7).

#### 3.1.7. 7-(Benzyloxy)-N-(4-bromophenyl)-2-oxo-2*H*-chromene-3-carboxamide (RSH-5)

Brown solid; Yield: 45%; mp. 180–182 °C; *Rf*: 0.80 (7:13 EtOAc: hexane); IR (cm^−1^): 3496.13 (N-H), 3026.41 (C-H), 1703.20 (C=O lactone), 1599.04 (C=O amide), 1491.02 (C=C), 1375.29 (C-N), 1257.63 (C-C), 1124.54, 1018.45 (C-O-C); ^1^H NMR (500 MHz, CDCl_3_) δ: 5.19 (s, 2H, OCH_2_Ar), 6.97 (s, 1H, C_8_H-chrom), 7.04–7.06 (d, *J* = 10 Hz, 1H, C_5_H-chrom.), 7.42–7.44 (m, 5H, Ar), 7.47–7.49 (d, *J* = 10 Hz, 2H, Ar), 7.63–7.65 (d, *J* = 10 Hz, 3H, Ar, C_6_H-chrom.), 8.93 (s, 1H, C_4_H-chrom.), 10.83 (s, 1H, amide) (Figure S8); HR-MS (ESI): *m*/*z* calcd for [M+H]^+^ C_23_H_16_NO_4_Br 450.28; found 450.0329 (Figure S9).

#### 3.1.8. 7-(Benzyloxy)-N-(4-hydroxyphenyl)-2-oxo-2*H*-1-benzopyran-3-carboxamide (RSH-6)

Yellow solid; Yield: 60%; mp: 160–161 °C; *Rf*: 0.64 (7:13 EtOAc: hexane); IR (cm^−1^): 3419.56 (N-H), 3045.39 (C-H), 1693.38 (C=O lactone), 1622.02 (C=O amide), 1510.16 (C=C), 1367.44 (C-N), 1236.29 (C-C), 1020.27 (C-O-C); ^1^H NMR (500 MHz, CDCl_3_) δ: 5.19 (s, 2H, OCH_2_Ar), 6.83–6.85 (d, 2H, Ar), 6.97 (s, 1H, C_8_H-chrom.), 7.03–7.05 (d, *J* = 10 Hz, 1H, C_5_H-chrom), 7.38–7.39 (d, *J* = 5 Hz, 2H, Ar), 7.41–7.44 (m, 3H, Ar), 7.59–7.61 (d, *J* = 10 Hz, 2H, Ar.), 7.62–7.63 (d, *J* = 5 Hz, 1H, C_6_H-chrom.), 8.93 (s, 1H, C_4_H-chrom.), 10.66 (s, 1H, amide); (Figure S10); ^13^C NMR (125 MHz, CDCl_3_) δ: 163.05, 159.65, 148.56, 131.08, 130.40, 128.86, 128.61, 127.55, 122.30, 115.90, 115.64, 115.07, 114.84, 101.39, 89.08, 70.86, 29.70, 29.67; HR-MS (ESI): *m*/*z* calcd for [M+H]^+^ C_23_H_18_NO_5_ 388.39; found 388.1181 (Figure S11).

#### 3.1.9. 7-(Benzyloxy)-N-(3,4-dimethoxyphenyl)-2-oxo-2*H*-1-benzopyran-3-carboxamide (RSH-7)

Yellow solid; Yield: 65%; mp. 180–181 °C; *Rf*: 0.56 (7:13 EtOAc: hexane); IR (cm^−1^): 3419.56 (N-H), 3045.39 (C-H), 1693.38 (C=O lactone), 1622.02 (C=O amide), 1510.16 (C=C), 1367.44 (C-N), 1236.29 (C-C), 1020.27 (C-O-C); ^1^H NMR (500 MHz, CDCl_3_) δ: 3.89 (s, 3H, OCH_3_), 3.93 (s, 3H, OCH_3_), 5.19 (s, 2H, OCH_2_Ar), 6.85–6.87 (s, 1H, Ar), 6.97–6.98 (s, 1H, C_8_H-chrom.), 7.03–7.05 (d, *J* = 10 Hz, 1H, C_5_H-chrom), 7.17–7.19 (d, *J* = 10 Hz, 1H, Ar), 7.42–7.44 (m, 5H, Ar), 7.50–7.51 (s, 1H, Ar), 7.61–7.63 (d, *J* = 10 Hz, 1H, C_4_H-chrom), 8.93 (s, 1H, C_4_H-chrom.), 10.70 (s, 1H, amide) (Figure S12); ^13^C NMR (125 MHz, CDCl_3_) δ: 164.11, 162.23, 159.60, 156.61, 149.28, 148.53, 146.08, 135.24, 131.50, 131.10, 128.88, 128.63, 127.55, 115.05, 114.89, 112.72, 111.33, 105.05, 101.4, 70.86, 56.11, 29.70 (Figure S13); HR-MS (ESI): *m*/*z* calcd for [M+H]^+^ C_25_H_22_NO_6_ 432.44; found 432.1441 (Figure S14).

#### 3.1.10. 7-(Benzyloxy)-2-oxo-N-phenyl-2*H*-1-benzopyran-3-carboxamide (RSH-8)

Pale Yellow solid; Yield: 43%; mp. 220–221 °C; *Rf*: 0.69 (7:13 EtOAc: hexane); IR (cm^−1^): 3423.41 (N-H), 3047.32 (C-H), 1701.10 (C=O lactone), 1608.52 (C=O amide), 1496.66 (C=C), 1373.22 (C-N), 1222.79 (C-C), 1026.06 (C-O-C), ^1^H NMR (500 MHz, CDCl_3_) δ: 5.19 (s, 2H, OCH_2_Ar), 6.97 (s, 1H, C_8_H-chrom), 7.03–7.05 (d, *J* = 10 Hz, 1H, C_5_H-chrom.), 7.14–7.17 (t, 1H, Ar), 7.38–7.39 (t, 3H, Ar), 7.42–7.44 (d, *J* = 10 Hz, 4H, Ar), 7.62–7.64 (d, *J* = 10 Hz, 1H, C_6_H-chrom), 7.73–7.74 (d, *J* = 5 Hz, 2H, Ar), 8.94 (s, 1H, C_4_H-chrom.), 10.79 (s, 1H, amide) (Figure S15); ^13^C NMR (125 MHz, CDCl_3_) δ: 164.04, 162.21, 159.63, 156.61, 148.82, 135.29, 131.15, 129.03, 128.61, 128.87, 127.56, 124.63, 122.07, 120.50, 115.11, 114.88, 112.73, 101.39, 70.85, 55.49 (Figure S16); HR-MS (ESI): *m*/*z* calcd for [M+H]^+^ C_23_H_18_NO_4_ 372.39; found 372.1234 (Figure S17).

#### 3.1.11. 7-(Benzyloxy)-N-(2-methylphenyl)-2-oxo-2*H*-1-benzopyran-3-carboxamide (RSH-23)

Brown solid; Yield: 50%; mp: 180–182 °C; *Rf*: 0.75 (7:13 EtOAc: hexane); IR (cm^−1^): 3475.49 (N-H), 3045.39 (C-H), 1704.96 (C=O lactone), 1693.38 (C=O amide), 1622.02 (C=O), 1510.16 (C=C), 1367.44 (C-N), 1236.29 (C-C), 1020.27 (C-O-C); ^1^H NMR (500 MHz, CDCl_3_) δ: 2.42 (s, 3H, CH_3_), 5.19 (s, 2H, OCH_2_Ar), 6.98 (s, 1H, C_5_H-chrom), 7.03–7.06 (d, 1H, C_8_H-chrom), 7.07–7.10 (d, 2H, Ar), 7.42–7.44 (m, 6H, Ar), 7.63–7.65 (d, *J* = 10 Hz, 1H, C_6_H-chrom), 8.24–8.25 (d, *J* = 5 Hz, 1H, Ar), 8.96 (s, 1H, C_4_H-chrom.), 10.73 (s, 1H, amide).

#### 3.1.12. 7-(Benzyloxy)-N-(3-methylphenyl)-2-oxo-2*H*-1-benzopyran-3-carboxamide (RSH-24)

Brown solid; Yield: 77%; mp: 210–211 °C; *Rf*: 0.77 (7:13 EtOAc: hexane); IR (cm^−1^): 3687.98 (N-H), 2935.20 (C-H), 1742.98, 1705.69 (C=O lactone), 1609.73 (C=O amide), 1447.24 (C=C), 1369.64 (C-N), 1281.15 (C-C), 1160.78 (C-O-C); ^1^H NMR (500 MHz, CDCl_3_) δ: 2.37 (s, 3H, CH_3_), 5.18 (s, 2H, OCH_2_Ar), 6.96–6.97 (s, 2H, Ar, C_5_H-chrom), 7.02–7.04 (d, 1H, C_8_H-chrom.), 7.41–7.43 (m, 5H, Ar), 7.53–7.55 (d, *J* = 10 Hz, 2H, Ar), 7.61–7.63 (d, *J* = 10 Hz, 1H, C_6_H-chrom), 8.93 (s, 1H, C_4_H-chrom.), 10.71 (s, 1H, amide) (Figure S40).

#### 3.1.13. 7-(Benzyloxy)-N-(3-chlorophenyl)-2-oxo-2*H*-1-benzopyran-3-carboxamide (RSH-25)

Pale brown solid; Yield: 83%; mp. 160–161 °C; *Rf*: 0.78 (7:13 EtOAc: hexane); IR (cm^−1^): 3291.32 (N-H), 2874.09, (C-H), 1701.97 (C=O lactone), 1683 (C=O amide), 1571.46 1489.39 (C=C), 1373.67 (C-N), 1210.84 (C-C), 1072.41, (C-O-C); ^1^H NMR (500 M Hz, CDCl_3_) δ: 5.19 (s, 2H, OCH_2_Ar), 6.97 (s, 1H, C_8_H-chrom), 7.04–7.06 (d, *J* = 10 Hz, 1H, C_5_H-chrom.), 7.12–7.13 (d, *J* = 5 Hz, 1H, Ar), 7.43–7.44 (d, *J* = 5 Hz, 5H, Ar), 7.35–7.55 (d, *J* = 10 Hz, 1H, Ar), 7.63–7.65 (d, *J* = 10 Hz, 1H, C_6_H-chrom), 7.89 (s, 1H, Ar), 8.93 (s, 1H, C_4_H-chrom.), 10.84 (s, 1H, amide).

#### 3.1.14. 7-(Benzyloxy)-N-(2-methoxyphenyl)-2-oxo-2*H*-1-benzopyran-3-carboxamide (RSH-26)

Yellowish brown solid; Yield: 84%; mp: 164–165 °C; *Rf*: 0.78 (7:13 EtOAc: hexane); IR (cm^−1^) 3781.39 (N-H), 2872.88 (C-H), 1710.65 (C=O), 1610.26 (C=O), 1442.30 (C=C), 1373.20 (C-N), 1208.71 (C-C), 1156.22 (C-O-C); ^1^H NMR (500 MHz, CDCl_3_) δ: 3.97 (s, 3H, OCH_3_), 5.18 (s, 2H, OCH_2_Ar), 6.93–6.94 (d, *J* = 10 Hz, 1H, C_5_H-chrom), 6.96 (s, 1H, C_8_H-chrom), 7.00–7.03 (m, 1H, Ar), 7.08–7.11 (t, 1H, Ar), 7.41–7.43 (m, 5H, Ar), 7.61–7.63 (d, 1H, C_6_H-chrom), 8.54–8.55 (d, 1H, Ar), 8.92 (s, 1H, C_4_H-chrom.), 11.24 (s, 1H, amide) (Figure S41).

#### 3.1.15. 7-(Benzyloxy)-N-(4-methoxyphenyl)-2-oxo-2*H*-1-benzopyran-3-carboxamide (RSH-27)

Yellowish brown solid; Yield: 62%; mp: 170–172 °C; *Rf*: 0.70 (7:13 EtOAc: hexane); IR (cm^−1^) 3788.88 (N-H), 2873.96 (C-H), 1704.62 (C=O), 1573.23 (C=O), 1490.74 (C=C), 1374.31 (C-N), 1212.71 (C-C), 1068.59 (C-O-C); ^1^H NMR (500 MHz, CDCl_3_) δ:3.82 (s, 3H, OCH_3_), 5.20 (s, 2H, OCH_2_Ar), 6.90–6.92 (d, *J* = 10 Hz, 2H, Ar), 6.96 (s, 1H, C_8_H-chrom), 7.03–7.05 (d, *J* = 10 Hz, 1H, C_5_H-chrom.), 7.42–7.44 (d, *J* = 10 Hz, 4H, Ar), 7.61–7.63 (d, *J* = 10 Hz, 2H, C_6_H-chrom.), 7.63–7.65 (m, 2H, Ar), 8.93 (s, 1H, C_4_H-chrom.), 10.67 (s, 1H, amide) (Figure S42).

#### 3.1.16. 7-(Benzyloxy)-N-(2,4-dimethylphenyl)-2-oxo-2*H*-1-benzopyran-3-carboxamide (RSH-28)

Brown solid; Yield: 85%; mp. 200–201 °C; *Rf*: 0.71 (7:13 EtOAc: hexane); IR (cm^−1^): 3789.33 (N-H), 2873.27 (C-H), 1704.44 (C=O lactone), 1610, 1573.50 (C=O amide), 1438.51 (C=C), 1373.28 (C-N), 1211.81 (C-C), 1068.78 (C-O-C); ^1^H NMR (500 MHz, CDCl_3_) δ: 2.31 (s, 1H, CH_3_), 2.37 (s, 1H, CH_3_), 5.19 (s, 2H, OCH_2_Ar), 6.96 (s, 1H, C_8_H-chrom), 7.03–7.05 (d, *J* = 10 Hz, 1H, C_5_H-chrom.), 7.05–7.07 (d, 3H, Ar), 7.43–7.44 (m, *J* = 5 Hz, 5H, Ar), 7.62–7.63 (d, *J* = 10 Hz, 1H, C_6_H-chrom), 8.07–8.08 (d, *J* = 5 Hz, Ar), 8.95 (s, 1H, C_4_H-chrom.), 10.64 (s, 1H, amide) (Figure S43); ^13^C NMR (125 MHz, CDCl_3_) δ: 164.02, 162.31, 159.73, 156.57, 148.63, 135.26, 134.58, 134.43, 133.65, 131.11, 130.88, 128.84, 128.73, 128.66, 128.16, 129.03, 128.61, 128.87, 127.56, 121.99, 115.26, 114.77, 112.71, 101.36, 70.65, 20.89, 18.00 (Figure S44); HR-MS (ESI): *m*/*z* calcd for [M+H]^+^ C_25_H_22_NO_4_ 400.45; found 400.1555 (Figure S45).

#### 3.1.17. 7-(Benzyloxy)-N-(2,5-dimethylphenyl)-2-oxo-2*H*-1-benzopyran-3-carboxamide (RSH-29)

Brown solid; Yield: 62%; mp. 171–172 °C; *Rf*: 0.75 (7:13 EtOAc: hexane); IR (cm^−1^): 3782.26 (N-H), 2876.28 (C-H), 1663.63 (C=O lactone), 1609 (C=O amide), 1490.00 (C=C), 1380.72 (C-N), 1214.66 (C-C), 1074.60 (C-O-C); ^1^H NMR (500 MHz, CDCl_3_): 2.36 (s, 1H, CH_3_), 2.37 (s, 1H, CH_3_), 5.19 (s, 2H, OCH_2_Ar), 6.89–6.91 (d, *J* = 10 Hz, 1H, Ar), 6.98 (s, 1H, C_8_H-chrom), 7.03–7.05 (d, *J* = 10 Hz, 1H, C_5_H-chrom.), 7.10–7.11 (d, *J* = 5 Hz, 1H, Ar), 7.43–7.44 (d, *J* = 5 Hz, 5H, Ar), 7.63–7.65 (d, *J* = 10 Hz, 1H, C_6_H-chrom), 8.07 (s, 1H, Ar), 8.95 (s, 1H, C_4_H-chrom.), 10.68 (s, 1H, amide) (Figure S46).

#### 3.1.18. 7-(Benzyloxy)-N-(2,6-dimethylphenyl)-2-oxo-2*H*-1-benzopyran-3-carboxamide (RSH-30)

Brown solid; Yield: 62%, mp. 172–173 °C; *Rf*: 0.71 (7:13 EtOAc: hexane); IR (cm^−1^): 3782.26 (N-H), 2876.28 (C-H), 1710.34 (C=O lactone), 1611.02 (C=O amide), 1490.00 (C=C), 1380.72 (C-N), 1214.66 (C-C), 1074.60 (C-O-C); ^1^H NMR (500 MHz, CDCl_3_): 2.30 (s, 2H, 2CH_3_), 5.21 (s, 2H, OCH_2_Ar), 6.99–7.00 (s, 1H, C_8_H-chrom), 7.05–7.07 (d, *J* = 10 Hz, 1H, C_5_H-chrom.), 7.14–7.15 (t, *J* = 5 Hz, 1H, Ar), 7.43–7.46 (m, *J* = 5 Hz, 5H, Ar), 7.63–7.65 (d, *J* = 10 Hz, 1H, C_6_H-chrom), 8.97 (s, 1H, C_4_H-chrom.), 10.14 (s, 1H, amide) (Figure S47).

#### 3.1.19. 7-(Benzyloxy)-N-(2-chlorophenyl)-2-oxo-2*H*-1-benzopyran-3-carboxamide (RSH-31)

Pale brown solid, Yield: 52%, mp. 120–122 °C; *Rf*: 0.75 (7:13 EtOAc: hexane); IR (cm^−1^): 2882.60, (C-H), 1708.89 (C=O lactone), 1611.68 (C=O amide), 1439.75 (C=C), 1374.85 (C-N), 1207.47 (C-C), 979.89 (C-O-C); ^1^H NMR (500 MHz, CDCl_3_) δ: 5.21 (s, 2H, OCH_2_Ar), 6.99 (s, 1H, C_8_H-chrom), 7.07–7.09 (d, *J* = 10 Hz, 1H, C_5_H-chrom.), 7.10–7.12 (t, 1H, Ar), 7.32–7.35 (t, 1H, Ar), 7.44–7.46 (m, 5H, Ar), 7.65–7.66 (d, *J* = 5 Hz, 1H, C_6_H-chrom), 7.57–7.59 (d, *J* = 10 Hz, 1H, Ar), 8.96 (s, 1H, C_4_H-chrom.), 11.30 (s, 1H, amide) (Figure S48).

#### 3.1.20. Synthesis of 7-(2-Bromoethoxy)-3-propanoyl-2*H*-1-benzopyran-2-one (RSH-12)

A suitable quantity of ethyl 7-hydroxy-2-oxo-2*H*-1-benzopyran-3-carboxylate (0.236 g, 1.0 mmol) and potassium carbonate (0.828 g, 6 mmol) were mixed in 10 mL of DMF and heated for 15 min at 75 °C. In the reaction mixture, 1,2-dibromoethane (0.561 mL, 1.5 mmol) was dissolved in 5 mL of DMF and then heated at 75 °C for 2–3 h. As soon as the reaction was complete, the product was washed with water and dried. For purification of the product, column chromatography (hexane: ethyl acetate) was used. White powder; % Yield 80%, *Rf:* 0.69 (7:13 EtOAc: hexane); mp. 120–121 °C, IR (cm^−1^): 1758.96 (C=O), 1620.09 (C=O), 1496.66 (C=C), 1282.57 (C-C), 1022.20 (C-O-C), 669.25 (C-Br); ^1^H NMR (500 MHz, CDCl_3_) δ: 1.39–1.42 (t, 3H, CH_3_), 3.67–3.69 (t, 2H, CH_2_), 4.37–4.40 (q, 2H, CH_2_), 4.40–4.42 (t, 2H, CH_2_), 6.81–6.82 (s, 1H, C_5_H-chrom), 6.91–6.93 (d, *J* = 10 Hz, 1H, C_8_H-chrom.), 7.52–7.53 (d, *J* = 5 Hz, 1H, C_6_H-chrom.), 8.50 (s, 1H, C_4_H-chrom.).

#### 3.1.21. Synthesis of Ethyl 7-[2-(2,3-Dimethylphenoxy)ethoxy]-2-oxo-2*H*-1-benzopyran-3-carboxylate (RSH-10)

2,3-dimethylphenol (0.13 mL, 1.0 mmol) and potassium carbonate (0.414 g, 3 mmol) were mixed in DMF (10 mL) and were heated (75 °C) for 15 min. Next, 7-(2-bromoethoxy)-3-propanoyl-2*H*-1-benzopyran-2-one (0.34 g, 1 mmol) dissolved in DMF (5 mL) was added to the mixture and further heated for 4 h. Once the reaction had been completed, the mixture was poured into cold water. The precipitated product was filtered, washed, and dried. The product is purified using column chromatography. Brown powder; % Yield: 55%; mp. 112–113 °C; *Rf*: 0.75 (7:13 EtOAc: hexane); IR (cm^−1^): 2941.24 (C-H), 1768.60 (C=O ester), 1616.24 (C=O), 1488.94 (C=C), 1201.57 (C-C), 1037.63 (C-O-C);^1^H NMR (500 MHz, CDCl_3_) δ: 1.39–1.42 (t, 3H, CH_3_), 2.13 (s, 3H, CH_3_), 2.27 (s, 3H, CH_3_), 4.34–4.36 (q, 2H, CH_2_), 4.39–4.41 (m, 1H, CH_2_), 4.42–4.44 (q, 3H, CH_2_), 6.74–6.76 (d, *J* = 10 Hz, 1H, Ar), 6.82–6.83 (d, *J* = 5 Hz, 1H, Ar) 6.89–6.90 (s, 1H, C_8_H-chrom.), 6.94–6.96 (d, *J* = 10 Hz, 1H, C_5_H-chrom), 7.05–7.08 (t, 1H, Ar), 7.51–7.52 (d, *J* = 5 Hz, 1H, C_6_H-chrom), 8.51 (s, 1H, C_4_H-chrom.) (Figure S18); ^13^C NMR (125 MHz, CDCl_3_) δ: 164.27, 163.46, 157.49, 157.10, 156.33, 148.89, 138.29, 130.76, 125.86, 123.09, 114.38, 114.02, 111.87, 109.55, 103.44, 101.16, 99.54, 67.53, 66.75, 61.75, 20.09, 14.29, 11.70 (Figure S19); HR-MS (ESI): *m*/*z* calcd for [M+H]^+^ C_22_H_23_O_6_383.41; found 383.1982 (Figure S20).

#### 3.1.22. Synthesis of Ethyl 7-[2-(2,5-Dimethylphenoxy)ethoxy]-2-oxo-2*H*-benzopyran-3-carboxylate (RSH-11)

2,5-dimethylphenol (0.122 g, 1.0 mmol) and potassium carbonate (0.414 g, 3 mmol) in 10 mL of DMF were heated at 75 °C for 15 min. Next, 7-(2-bromoethoxy)-3-propanoyl-2*H*-1-benzopyran-2-one (0.34 g, 1 mmol) dissolved in DMF (5 mL) was added to the reaction mixture, and further heated (75 °C) for 4 h. The reaction mixture was poured into chilled water. Using a vacuum, the product was filtered, washed, and dried. A column chromatography technique (hexane: ethyl acetate) was used for the purification of the product. Brown powder; % Yield: 60%; mp. 110–111 °C; *Rf:* 0.80 (7:13 EtOAc: hexane); IR (cm^−1^): 2941.24 (C-H), 1757.03 (C=O ester), 1610.45 (C=O), 1492.80 (C=C), 1218.9 (C-C), 1041.49 (C-O-C); ^1^H NMR (500 MHz, CDCl_3_) δ: 1.39–1.42 (t, 3H, CH_3_), 2.16 (s, 3H, CH_3_), 2.33 (s, 3H, CH_3_), 4.34–4.36 (q, 2H, OCH_2_), 4.38–4.41 (m, 2H, CH_2_), 4.42–4.44 (m, 2H, CH_2_), 6.69 (s, 1H, Ar), 6.71–6.72 (d, *J* = 5 Hz, 1H, Ar), 6.89–8.90 (s, 1H, C_5_H-chrom), 6.94–6.96 (d, *J* = 10 Hz, 1H, C_8_H-chrom), 7.01–7.03 (d, *J* = 10 Hz, 1H, Ar), 7.51–7.52 (d, *J* = 5 Hz, 1H, C_6_H-chrom), 8.51 (s, 1H, C_4_H-chrom) (Figure S21).

#### 3.1.23. Synthesis of 7-[2-(2,3-Dimethylphenoxy)ethoxy]-2-oxo-2*H*-1-benzopyran-3-carboxylic Acid

A suitable quantity of ethyl 7-[2-(2,3-dimethylphenoxy)ethoxy]-2-oxo-2*H*-1-benzopyran-3-carboxylate (0.237 g) was added in dimethyl sulfoxide (DMSO) as a solvent. 4 mL 2N NaOH was added and refluxed at 100 °C for 2 h. In the following step, 2N HCl was added to neutralize the solution, which resulted in precipitation. The resulted precipitate was filtered, and the resulted product was used as is for further reaction. Pale brown powder; % Yield: 75%; mp. 130–133 °C; *Rf*: 0.48 (7:13 EtOAc: hexane).

#### 3.1.24. Synthesis of 7-[2-(2,5-Dimethylphenoxy)ethoxy]-2-oxo-2*H*-1-benzopyran-3-carboxylic Acid

A suitable quantity of ethyl 7-[2-(2,5-dimethylphenoxy)ethoxy]-2-oxo-2*H*-1-benzopyran-3-carboxylate (0.237 g) was dissolved in dimethyl sulfoxide (DMSO) as a solvent. A solution of 4 mL 2N NaOH was added to it and was refluxed at 100 °C for two h. In the following step, 2N HCl was added to neutralize the solution, which resulted in precipitation. It was filtered and washed twice with water. The resulting product was used as is for further reaction. Pale brown powder; % Yield: 80%; mp. 132–134 °C; *Rf*: 0.45 (7:13 EtOAc: hexane).

#### 3.1.25. Synthesis of Compounds (RSH-13–RSH-17) [31]

A 7-[2-(2,3-dimethylphenoxy)ethoxy]-2-oxo-2*H*-1-benzopyran-3-carboxylic acid (0.354 g, 1.0 mmol) was dissolved in DMF previously kept in ice. Further Hexafluorophosphate Azabenzotriazole Tetramethyl Uronium (HATU) (0.274 g, 0.72 mmol) was added to the above solution, and it was stirred for 30 min. Next, corresponding substituted anilines (0.75 mmol) and DIPEA (0.127 g, 1 mmol) were added and stirred overnight. On reaction completion, the mixture is dumped in ice and extracted with ethyl acetate. The ethyl layer was dried with magnesium sulfate and evaporated. The crude product was purified using column chromatography to afford final compounds (RSH-13–RSH-17).

#### 3.1.26. 7-[2-(2,3-Dimethylphenoxy)ethoxy]-N-(4-hydroxyphenyl)-2-oxochromene-3-carboxamide (RSH-13)

Brown solid; Yield 45%; mp. 210–211 °C; *Rf:* 0.60 (7:13 EtOAc: hexane); IR (cm^−1^): 2918.40 (C-H), 1703.99 (C=O amide), 1599.04 (C=O), 1445.66 (C=C), 1232.56 (C-C), 1373.52 (C-N), 1037.74 (C-O-C); ^1^H NMR (500 MHz, CDCl_3_) δ: 2.14 (s, 3H, CH_3_), 2.27 (s, 3H, CH_3_), 4.36–4.38 (t, 2H, CH_2_), 4.45–4.47 (t, 2H, CH_2_), 6.75–6.77 (d, *J* = 10 Hz, 1H, Ar), 6.82–6.86 (t, 3H, Ar), 6.98 (s, 1H, C_8_H-chrom), 7.02–7.04 (d, 2H, *J* = 10 Hz, C_5_H-chrom) 7.05–7.07 (d, *J* = 10 Hz, 1H, Ar), 7.60–7.61 (d, *J* = 10 Hz, 1H, C_6_H-chrom), 7.63–7.65 (d, *J* = 10 Hz, 2H, Ar), 8.94 (s, 1H, C_4_H-chrom.), 10.68 (s, 1H, amide) (Figure S23); ^13^C NMR (125 MHz, CDCl_3_) δ: 164.16, 161.65, 159.84, 156.57, 154.56, 148.14, 137.87, 132.12, 130.13, 126.42, 124.95, 122.95, 122.03, 115.82, 114.69, 112.87, 110.26, 101.51, 68.13, 67.18, 20.17, 11.90 (Figure S24).

#### 3.1.27. 7-[2-(2,3-Dimethylphenoxy)ethoxy]-N-(3,4-dimethoxyphenyl)-2-oxochromene-3-carboxamide (RSH-14)

Brown solid; Yield: 60%; mp. 140–141 °C; *Rf:* 0.58 (7:13 EtOAc: hexane); IR (cm-1): 2914.40 (C-H), 1703.99 (C=O lactone), 1583.51 (C=O amide), 1450.52 (C=C), 1238.34 (C-C), 1039.57 (C-O-C);^1^H NMR (500 MHz, CDCl_3_) δ: 2.14 (s, 3H, CH_3_), 2.27 (s, 3H, CH_3_), 3.89 (s, 3H, CH_3_), 3.94 (s, 3H, CH_3_), 4.36–4.38 (t, 2H, CH_2_), 4.45–4.47 (t, 2H, CH_2_), 6.75–6.77 (d, *J* = 10 Hz, 1H, C_8_H-chrom.), 6.82–6.84 (d, *J* = 10 Hz, 1H, Ar), 6.86–6.88 (d, *J* = 10 Hz, 1H, Ar), 6.99 (s, 1H, Ar), 7.02–7.07 (m, 2H, Ar), 7.17–7.19 (d, *J* = 10 Hz, 1H, Ar), 7.51 (s, 1H, Ar), 7.63–7.64 (d, *J* = 5 Hz, 1H, C_5_H-chrom), 8.94 (s, 1H, C_4_H-chrom.), 10.72 (s, 1H, amide) (Figure S25); ^13^C NMR (125 MHz, CDCl_3_) δ: 164.23, 161.58, 160.09, 156.56, 149.17, 148.31, 147.02, 146.01, 137.87, 132.15, 131.98, 126.42, 124.95, 122.95, 115.98, 114.67, 112.94, 110.26, 105.40, 103.24, 101.53, 98.74, 68.15, 67.18, 56.16, 20.17, 11.89 (Figure S26); HR-MS (ESI): *m*/*z* calcd for [M+H]^+^ C_28_H_27_O_7_N 490.52; found 490.1875 (Figure S27).

#### 3.1.28. 7-[2-(2,3-Dimethylphenoxy)ethoxy]-N-(4-methylphenyl)-2-oxochromene-3-carboxamide (RSH-15)

Brown solid; Yield 52%; mp. 230–231 °C; *Rf*: 0.66 (7:13 EtOAc: hexane); IR (cm^−1^): 3417.14 (N-H), 2869.58 (C-H), 1702.95 (C=O lactone), 1572.63 (C=O amide), 1488.26 (C=C), 1154.90 (C-C), 1373.74 (C-N), 961.90 (C-O-C); ^1^H NMR (500 MHz, CDCl_3_) δ: 2.14 (s, 3H, CH_3_), 2.27 (s, 3H, CH_3_), 2.35 (s, 3H, CH_3_), 4.35–4.37 (t, 2H, CH_2_), 4.45–4.47 (t, 2H, CH_2_), 6.75–6.77 (d, *J* = 10 Hz, 1H, Ar), 6.82–6.84 (d, 1H, Ar), 6.99 (s, 1H, C_8_H-chrom.), 7.02–7.04 (d, 1H, C_5_H-chrom), 7.05–7.09 (t, 1H, Ar), 7.17–7.19 (t, 1H, Ar), 7.61–7.65 (d, 3H, Ar, C_6_H-chrom), 8.95 (s, 1H, C_4_H-chrom.), 10.73 (s, 1H, amide) (Figure S28); ^13^C NMR (125 MHz, CDCl_3_) δ: 164.20, 162.20, 159.72, 156.63, 156.32, 148.64, 138.31, 135.30, 134.29, 131.13, 129.54, 128. 87, 127.56, 125.87, 125.12, 123.12, 120.50, 115.18, 114.84, 112.77, 109.55, 101.39, 70.86, 67.61, 66.75, 22.95, 20.09, 11.70 (Figure S29); HR-MS (ESI): *m*/*z* calcd for [M+H]^+^ C_27_H_26_NO_5_ 444.50; found 444.15 (Figure S30).

#### 3.1.29. 7-[2-(2,3-Dimethylphenoxy)ethoxy]-N-(4-chlorophenyl)-2-oxochromene-3-carboxamide (RSH-16)

Brown solid; Yield 82%; mp. 210–212 °C; *Rf*: 0.81 (7:13 EtOAc: hexane); IR (cm^−1^): 3783.71 (N-H), 2875.22 (C-H), 1710.24 (C=O lactone), 1605.84 (C=O amide), 1441.53 (C=C), 1143.52 (C-C), 1372.79 (C-N), 989.17 (C-O-C); ^1^H NMR (500 MHz, CDCl_3_) δ: 2.14 (s, 3H, CH_3_), 2.27 (s, 3H, CH_3_), 4.36–4.38 (t, 2H, CH_2_), 4.46–4.48 (t, 2H, CH_2_), 6.75–6.77 (d, *J* = 10 Hz, 1H, Ar), 6.82–6.84 (d, *J* = 10 Hz, 1H, Ar), 6.99 (s, 1H, C_8_H-chrom.), 7.02–7.04 (d, 1H, C_5_H-chrom), 7.07–7.09 (t, 1H, Ar), 7.33–7.35 (d, 2H, *J* = 10 Hz, Ar), 7.64–7.66 (d, *J* = 10 Hz, 1H, C_6_H-chrom), 7.69–7.71 (d, *J* = 10 Hz, 2H, Ar,), 8.96 (s, 1H, C_4_H-chrom.), 10.87 (s, 1H, amide) (Figure S31); HR-MS (ESI): *m*/*z* calcd for [M+H]^+^ C_26_H_23_NO_5_Cl 464.92; found 464.11 (Figure S32).

#### 3.1.30. 7-[2-(2,3-Dimethylphenoxy)ethoxy]-N-phenyl-2-oxochromene-3-carboxamide (RSH-17)

Brown solid, Yield: 55%, mp. 180–181 °C; *Rf:* 0.85 (7:13 EtOAc: hexane); IR (cm^−1^): 3378.54 (N-H), 2872.87 (C-H), 1662.49 (C=O lactone), 1570.35 (C=O amide), 1486.37 (C=C), 1136.60 (C-C), 1394.37 (C-N), 1081.78 (C-O-C); ^1^H NMR (500 MHz, CDCl_3_) δ: 2.14 (s, 3H, CH_3_), 2.27 (s, 3H, CH_3_), 4.36–4.38 (t, 2H, CH_2_), 4.45–4.47 (t, 2H, CH_2_), 6.75–6.77 (d, *J* = 10 Hz, 1H, Ar), 6.82–6.84 (d, *J* = 10 Hz, 1H, Ar), 6.99 (s, 1H, C_8_H-chrom.), 7.02–7.04 (d, *J* = 10 Hz, 1H, C_5_H-chrom), 7.05–7.09 (t, 1H, Ar), 7.15–7.17 (t, 1H, Ar), 7.37–7.40 (t, 2H, Ar), 7.64–7.65 (d, 1H, C_6_H-chrom), 7.73–7.75 (d, *J* = 10 Hz, 2H, Ar), 8.96 (s, 1H, C_4_H-chrom.), 10.80 (s, 1H, amide) (Figure S33); ^13^C NMR (125 MHz, CDCl_3_) δ: 164.27, 162.19, 159.87, 156.67, 156.30, 148.81, 138.31, 131.29, 11.17, 129.04, 125.73, 124.63, 123.12, 120.53, 115.08, 114.63, 112.74, 109.54, 101.18, 67.62, 66.74, 20.09, 11.70 (Figure S34); HR-MS (ESI): *m*/*z* calcd for [M+H]^+^ C_26_H_24_NO_5_ 430.47; found 430.1653 (Figure S35).

#### 3.1.31. Synthesis of Compounds (RSH-18–RSH-22) [32]

7-[2-(2,5-dimethylphenoxy)ethoxy]-2-oxo-2*H*-1-benzopyran-3-carboxylic acid (0.354 g, 1.0 mmol) was dissolved in 4–5 mL of DMF previously kept on ice. Further Hexafluorophosphates Azabenzotriazole Tetramethyl Uronium (HATU) (0.274 g, 0.72 mmol) was added to the above solution, and it was stirred for 30 min. Then, corresponding substituted anilines (0.75 mmol) and DIPEA (0.127 g, 1 mmol) were added and stirred at RT overnight. The mixture is then added to ice and extracted with ethyl acetate. Using magnesium sulfate, the ethyl layer was dried and rotary evaporated. Final pure compounds were obtained using column chromatography (Mobile Phase: ethyl acetate and n-hexane) (RSH-18–RSH-22).

#### 3.1.32. 7-[2-(2,5-Dimethylphenoxy)ethoxy]-N-phenyl-2-oxochromene-3-carboxamide (RSH-18)

Brown solid; Yield 65%; mp. 170–172 °C; *Rf*: 0.75 (7:13 EtOAc: hexane); IR (cm^−1^) 3278.04 (N-H), 2873.92 (C-H), 1705.14 (C=O lactone), 1573.13 (C=O amide), 1491.61 (C=C), 1142.52 (C-C), 1374.34 (C-N), 1072.42 (C-O-C); ^1^H NMR (500 MHz, CDCl_3_) δ: 2.17 (s, 3H, CH_3_), 2.33 (s, 3H, CH_3_), 4.36–4.38 (t, 2H, CH_2_), 4.46–4.48 (t, 2H, CH_2_), 6.69 (s, 1H, Ar), 6.72–6.73 (d, *J* = 5 Hz, 1H, Ar), 6.99 (s, 1H, C_5_H-chrom.), 7.02–7.04 (d, *J* = 10 Hz, 1H, Ar), 7.14–7.16 (d, *J* = 10 Hz, 1H, C_8_H-chrom), 7.36–7.40 (t, 3H, Ar), 7.64–7.65 (d, *J* = 5 Hz, 1H, C_6_H-chrom), 7.73–7.75 (d, *J* = 10 Hz, 2H Ar), 8.96 (s, 1H, C_4_H-chrom.), 10.80 (s, 1H, amide) (Figure S36).

#### 3.1.33. 7-[2-(2,5-Dimethylphenoxy)ethoxy]-N-(4-methylphenyl-2-oxochromene-3-carboxamide (RSH-19)

Brown solid; Yield 55%; mp. 190–192 °C; *Rf*: 0.65 (7:13 EtOAc: hexane); IR (cm^−1^): 3758.39 (N-H), 2881.75 (C-H), 1703.20 (C=O lactone), 1600.97 (C=O amide), 1483.1 (C=C), 1253.71 (C-C), 1383.01 (C-N), 1018.45 (C-O-C); ^1^H NMR (500 MHz, CDCl_3_) δ: 2.16 (s, 3H, CH_3_), 2.33 (s, 3H, CH_3_), 2.35 (s, 3H, CH_3_), 4.36–4.38 (t, 2H, CH_2_), 4.45–4.47 (t, 2H, CH_2_), 6.69 (s, 1H, Ar), 6.71–6.73 (d, *J* = 10 Hz, 1H, Ar), 6.98 (s, 1H, C_5_H-chrom), 7.02–7.03 (d, *J* = 5 Hz, 2H, C_8_H-chrom), 7.17–7.19 (d, 2H, Ar), 7.61–7.64 (dd, 3H, C_6_H-chrom, Ar), 8.94 (s, 1H, C_4_H-chrom.), 10.72 (s, 1H, amide) (Figure S37).

#### 3.1.34. 7-[2-(2,5-Dimethylphenoxy)ethoxy]-N-(4-hydroxyphenyl-2-oxochromene-3-carboxamide (RSH-21)

Brown solid, Yield: 45%, mp. 180–182 °C; *Rf:* 0.63 (7:13 EtOAc: hexane); IR (cm^−1^): 3790.14 (N-H), 2880.17 (C-H), 1712.90 (C=O lactone), 1611.77 (C=O amide), 1496.42 (C=C), 1207.96 (C-C), 1373.31 (C-N), 976.34 (C-O-C); ^1^H NMR (500 MHz, CDCl_3_) δ: 2.18 (s, 3H, CH_3_), 2.31 (s, 3H, CH_3_), 4.36–4.38 (t, 2H, CH_2_), 4.45–4.47 (t, 2H, CH_2_), 6.69 (s, 1H, Ar), 6.72–6.73 (d, *J* = 5 Hz, 1H, Ar), 6.82–6.88 (d, 3H, Ar), 6.98 (s, 1H, C_5_H-chrom), 7.02–7.09 (m, 2H, C_8_H-chrom), 7.58–7.64 (d, 3H, Ar), 8.94 (s, 1H, C_4_H-chrom.), 10.68 (s, 1H, amide) (Figure S38); HR-MS (ESI): *m*/*z* calcd for [M+H]^+^ C_26_H_24_O_6_ N 446.47; found 446.1609 (Figure S39).

### 3.2. In Vitro Studies

#### 3.2.1. Cell Culture

Human NCI-H716 enteroendocrine cell line was purchased from the American Type Culture Collection (ATCC, #CCL-251, https://www.atcc.org/products/ccl-251 accessed on 6 December 2017). Cells were cultured in RPMI 1640 with L-glutamine (GenClone). Cell culture media were supplemented with 10% fetal bovine serum (FBS), 100 units/mL of penicillin, and 100 µg/mL of streptomycin (GenClone). Cells were grown in this RPMI with FBS- and antibiotic-containing ‘complete’ media at 37 °C in a culturing incubator in an atmosphere of 5% CO_2_.

#### 3.2.2. Cell Viability Assay

NCI-H716 cells were treated with the indicated compounds in complete media. Compounds were dissolved in DMSO, and DMSO was used as a negative control, while the DMSO concentration was kept constant in all treatments with dose responses. Cells were incubated with compounds overnight in complete RPMI media in a culturing incubator at 37 °C in a 5% CO_2_ atmosphere. Cell viability was measured using Cell Titer-Glo Luminescent Cell Viability Assay (G7570, Promega, Madison, WI, USA) according to the manufacturer’s instructions. Luminescence was measured using a Varioskan Lux plate reader (ThermoFisher Scientific, Waltham, MA, USA). Luminescence reading was normalized to the DMSO control and plotted as relative cell viability.

#### 3.2.3. GLP-1 Secretion Assay

NCI-H716 cells are a suspension cell line, and need to be attached and polarized for GLP-1 secretion. Cells were seeded in plastic cell culture plates coated with Matrigel (Corning Life Sciences, Tewksbury, MA, USA, catalog no. 356234), which was diluted while ice cold in refrigerated Hanks’ balanced salt solution (HBSS, Gibco, Thermo Fisher Scientific, Waltham, MA, USA) according to the manufacturer’s instructions. The cells were allowed to grow for 2 days in complete RPMI medium with FBS and antibiotics as detailed in Section 3.2.1. On the day of the treatment with compounds, Matrigel-attached cells were washed with low-serum (0.5% FBS) RPMI media without antibiotics. Compounds were diluted in DMSO and added to cells carefully in the low-serum medium (0.5% FBS, RPMI 1640) without antibiotics. DMSO concentrations were kept constant throughout the treatments and used as a negative control. After a 2 h incubation, the cell culture media containing secreted GLP-1 were collected in 96-well plates and stored at −20 °C until further analysis. Secreted total GLP-1 levels were then measured using the Invitrogen, Waltham, MA, USA, Human GLP-1 (1-37a) ELISA Kit from ThermoFisher Scientific, Waltham, MA, USA (catalog # EH221RB) according to the manufacturer’s instructions.

#### 3.2.4. Real-Time qPCR Assay

NCI-H716 enteroendocrine cells were treated with compounds in complete RPMI media with DMSO as the negative control, as detailed in Section 3.2.1 and Section 3.2.2. Cells were incubated with compounds overnight in complete RPMI media with FBS and antibiotics in a mammalian cell culture incubator at 37 °C in a 5% CO_2_ atmosphere. The next day, cells were collected in RNase-free Eppendorf tubes and washed with 1X PBS by centrifugation at 4 °C at 500× *g* to remove compound and media. Cell pellets were resuspended in TRIzol (Ambion, Life Technologies, Thermo Fisher Scientific, Waltham, MA, USA) and tubes were snap frozen in liquid N_2_ and stored at −80 °C until further analysis. Tubes with cells were thawed on ice for RNA extraction and kept on ice throughout the extraction process using TRIzol extraction and ethanol precipitation. Briefly, tubes were vortexed for 30 s to disrupt cell membranes and release RNA. Chloroform was added (200 mL chloroform/1 mL TRIzol) and vortexed for 30 s. Tubes were centrifuged at 12,000 rpm for 15 min at 4 °C. The clear top layer containing RNA was transferred to new RNase-free Eppendorf tubes, to which an equal volume of 2-propanol was added, and the tubes were inverted to mix (500 mL 2-propanol/1 mL TRIzol). Tubes were then centrifuged at 12,000 rpm for 10 min at 4 °C. The RNA pellet was washed with 70% EtOH (made with RNase and DNase-free water) and centrifuged at 14,000 rpm for 5 min at 4 °C. The RNA pellet was air-dried and resuspended in RNase-free water. cDNA synthesis was performed using the Maxima First Strand cDNA Synthesis Kit with DNase (ThermoFisher Scientific). qPCR was performed using the SYBR Green Master Mix (ThermoFisher Scientific) in a 384-well format using the QuantStudio 5 (ThermoFisher Scientific). The 2^−ΔΔCt^ method was used to calculate the relative change in gene expression normalized to the loading control. Data were then averaged for comparison between groups. qPCR primers used were human *TGR5*: Forward: 5′-CCTAGGAAGTGCCAGTGCAG-3′, Reverse: 5′-CTTGGGTGGTAGGCAATGCT-3′; human *GAPDH*: Forward: 5′-GAAGGTGAAGGTCGGAGT-3′, Reverse: 5′-CATGGGTGGAATCATATTGGAA-3′.

#### 3.2.5. CRISPR KO Cell Lines

Guide RNAs targeting human TGR5 and SpCas9 protein were purchased from EditCo (Redwood City, CA, USA). Transfection was performed using the Neon Transfection System (ThermoFisher Scientific) according to the manufacturer’s instructions. Briefly, gRNA (30 µM) and SpCas9 protein (20 µM) were combined to create an RNP solution and incubated for 10 min to allow gRNA and SpCas9 protein to complex. NCI-H716 cells were centrifuged at 150× *g* for 5 min, then resuspended in 50 µL of Buffer R (10 µL per 200k cells). A total of 10 μL of cell suspension was added to each RNP solution. Electroporation was performed with Buffer E as instructed by the manufacturer. Electroporation parameters used were 1700 V, 20 ms, 1 pulse. Cells were allowed to recover for 3–4 days, and knockout was confirmed with PCR and Sanger Sequencing.

#### 3.2.6. Luciferase Reporter Assay

For luciferase reporter assays for TGR5 activation, we used the cAMP response element (CRE)-driven luciferase construct pGL4.29 [luc2P/CRE/Hygro] plasmid (Promega Corporation, Madison, WI, USA) and the pGL4.74 [hRluc/CMV] plasmid (Promega Corporation) at a concentration of 2 μg/mL and 0.05 μg/mL of cell culture media volume, respectively. All plasmids were transfected using Opti-MEM (Gibco, Thermo Fisher Scientific, Waltham, MA, USA) and Lipofectamine 2000 (Invitrogen, Life Technologies, Grand Island, NY, USA). Transfections were performed in RPMI media without antibiotics with 10% FBS. After overnight incubation with plasmids, compounds were added in complete RPMI media with FBS and antibiotics, incubated overnight, and harvested the next day for luciferase assay. Cells were washed with PBS and lysed in PLB from the kit. Matrigel-attached cells were scraped in PLB. Luminescence was measured using the Dual-Luciferase Reporter Assay System (Promega Corporation) according to the manufacturer’s instructions. Luminescence readings were captured using the Varioskan Lux Reader. Luminescence was normalized to Renilla luciferase activity.

#### 3.2.7. Presto-Tango Assay

Human TGR5-Tango construct (GPBA-Tango, #66298) was purchased from Addgene, Watertown, MA, USA. HTLA cells were a kind gift from Dr. Richard Axel. A total of 4 million HTLA cells were seeded in a T-75 flask. At 80% confluence, 30 µg of GPBA-Tango plasmid was transfected into cells using Opti-MEM (Gibco) and Lipofectamine CRISPRMAX (Invitrogen, Life Technologies, Grand Island, NY, USA). After 24 h, cells were trypsinized and transferred to a PLL-coated 96-well plate. Cells were allowed to attach for 2 h at 37 °C in an atmosphere of 5% CO_2_. Compounds were added to the complete media and incubated overnight. BrightGlo (Promega, Madison, WI, USA) was used to trigger luminescence. Luminescence was measured using the Varioskan Lux Reader.

#### 3.2.8. ATF6α siRNA

ATF6α siRNA (Santa Cruz Biotechnology, Dallas, TX, USA) and negative siRNA (Ambion, Thermo Fisher Scientific, Waltham, MA, USA) transfection was performed using Opti-MEM and Lipofectamine RNAiMAX according to the manufacturer’s instructions. Briefly, siRNAs were resuspended and diluted in RNase-free water (SCBT) and added at a final concentration of 40 nM in RPMI media without FBS and antibiotics. After siRNA transfection, cells were incubated in antibiotic- and FBS-free media for 24 h and then complete RPMI media with FBS and antibiotics for an additional 24 h. Compounds were then added (48 h post-siRNA transfection) in complete RPMI media with FBS and antibiotics. After an overnight incubation, cells were harvested for RNA extraction as described in Section 3.2.4.

##### Subcellular Fractionation

Cells treated with compounds overnight were harvested, and subcellular fractionation was performed on the same day on fresh (never-frozen) samples. Cells were harvested from a T-75 flask into 500 μL of cold fractionation buffer by scraping. Fractionation buffer components: 20 mM HEPES (pH 7.4), 10 mM KCl, 2 mM MgCl_2_, 1 mM EDTA, 1 mM EGTA; just before use, 1 mM DTT, and Protease and Phosphatase inhibitor cocktail (Roche) was added. The fractionation buffer was chilled on ice for at least 15 min. Using a 1 mL syringe, cells were passed through a 27-gauge needle 10 times (or until all cells are lysed). Lysate was incubated on ice for 20 min, followed by centrifugation at 720× *g* for 5 min. The pellet was the crude nuclear fraction, and the supernatant contained the cytoplasm, membrane, and mitochondria. The supernatant was transferred into a fresh tube and kept on ice. The nuclear pellet was resuspended in 500 μL fractionation buffer and passed through a 25-gauge needle 10 times. Samples were centrifuged at 720× *g* for 10 min. The supernatant was discarded, and the pellet was snap frozen as the nuclear fraction. The cytoplasm, mitochondria, and membrane supernatant were centrifuged at 10,000× *g* for 5 min to pellet the mitochondria. The pellet was discarded, and the supernatant was snap frozen as the cytoplasm fraction.

#### 3.2.9. Western Blot Analysis

Protein was extracted from cell lysates using Cell Lysis Buffer (Cell Signaling Technology, Danvers, MA, USA) supplemented with Protease and Phosphatase inhibitor cocktail (Roche, Basel, Switzerland). Samples were vortexed for 30 s, followed by centrifugation at 10,000× *g* for 10 min to pellet debris. Supernatant was denatured with NuPAGE LDS Sample Buffer (ThermoFisher Scientific) with 1% β-mercaptoethanol, and boiled at 100 °C for 5 min. Samples were centrifuged briefly to collect condensates and loaded onto Bolt Bis-Tris Plus Mini Protein Gels, 4–12% (Life Technologies) for SDS PAGE in X-Cell SureLock Mini-Cell apparatus (Invitrogen). Transfer was performed in the X-Cell Sure Lock on PVDF membranes (Invitrogen), and stained with No-Stain Protein Labeling Reagent (Invitrogen) according to the manufacturer’s instructions. Blots were imaged for total protein on an iBright imager (ThermoFisher Scientific), then blocked with Pierce Protein-free blocking buffer (ThermoFisher Scientific). Blots were incubated with primary and HRP-conjugated secondary antibodies (Cell Signaling Technology) in 5% BSA. Blots were developed with the Pierce ECL Plus Western Blotting Substrate (ThermoFisher Scientific) and imaged on an iBright imager, and band intensity was quantified and normalized to No-Stain using Adobe Photoshop.

### 3.3. In Vivo Studies

#### 3.3.1. Animals

We ascribed to and followed the ARRIVE guidelines for all experiments. C57BL/6J male mice were purchased from Jackson Laboratories at five weeks of age. For acute experiments, mice were preconditioned on a high-fat WD (Inotiv TD.88137; 42% calories from fat; 30% *w*/*v* sucrose) for 8 weeks to induce obesity and glucose intolerance. Mice were housed in groups of two and had ad libitum access to food and water in a climate-controlled environment with a 12-h light/dark cycle. For acute drug dosing experiments, mice were assigned to weight-matched groups for drug testing: vehicle, INT-777, RSH-14, and RSH-30.

Guided by our acute experiment, we completed a power calculation to inform our chronic experiment (G*Power 3.1.9.6, Heinrich-Heinie Universität Düsseldorf). We calculated an effect size of 2.33 between RSH-14 and the vehicle. Thus, with 5 animals per group, we would be able to detect a difference in efficacy with an alpha of 0.05 and a power of 0.9. To account for loss, we started with 6 mice per group. For chronic dosing experiments, mice were assigned to weight-matched groups for 6 weeks of drug testing: vehicle, INT-777, RSH-14, and RSH-30. One mouse died during preconditioning, and thus the RSH-14 group had only 5 mice per group. A single mouse in the vehicle arm died after a traumatic gavage, and all data after this were excluded. Animal data were collected in an unblinded fashion.

C57BL/6J mice purchased from Jackson Laboratories at five weeks of age were preconditioned on a high-fat WD (Inotiv TD.88137; 42% calories from fat; 30% *w*/*v* sucrose) for 5 weeks to induce obesity and glucose intolerance. Mice were housed in groups of two and had ad libitum access to food and water in a climate-controlled environment with a 12-h light/dark cycle. All procedures were approved by the Institutional Animal Care and Use Committee (UW-Madison), and animals were cared for according to guidelines set forth by the American Association for Laboratory Animal Science in an AALAC-accredited facility.

#### 3.3.2. Functional Glucose Testing

Animal body weights and food consumption were quantified throughout the experiment. For MTT assays, animals were fasted for 4 h (7 a.m. to 11 a.m.). ITT assays were performed without a fast. The MTT assay was performed in week 4. Fasted mice were administered an oral bolus of Ensure (Abbot Nutrition, IL, USA; 10 mL/kg) and blood glucose was measured at time points 0, 15, 30, 60, and 120 min. The ITT assay was performed in week 5. Non-fasted mice received an intraperitoneal injection of regular insulin (0.5 U/kg; Novolin^®^ R, Novo Nordisk). Blood glucose was measured at time points 0, 15, 30, 60, and 90 min. The blood glucose measurements were made using a Bayer Contour glucose meter with test strips. Total GLP-1 was measured using the total GLP1 ELISA kit (Thermo Fisher BMS2194) according to the manufacturer’s instructions.

#### 3.3.3. Pancreatic Islet Isolation

Pancreatic islets were isolated by collagenase (Sigma #C7657-5G, Sigma-Aldrich, St. Louis, MO, USA) digestion of the pancreas and purified by gradient centrifugation using Lymphocyte Separation Media (Corning #25-072-CV, Glendale, AZ, USA). Islets were hand-picked and cultured in RPMI (Gibco #11879-020, Paisley, Scotland) containing 8 mM glucose, supplemented with 10% FBS (Corning 35-010-CV), 100 I.U./mL penicillin, and 100 μg/mL streptomycin (Corning 30-001-CI) at 37 °C and 5% CO_2_. (PMC7028949).

#### 3.3.4. Glucose-Stimulated Insulin Secretion

Following overnight culture, glucose-stimulated insulin secretion assays were performed in Krebs-Ringer Bicarbonate buffer (KRB) at pH 7.4 containing (mmol/L) NaCl (120), KCl (4.8), CaCl_2_ (2.5), MgCl_2_ (1.2), NaHCO_3_ (24), and 1 g/L BSA. Batches of 10 islets were pre-incubated for 45 min in 1 mL of KRB containing 2 mM glucose in non-coated 24-well plates (Costar #3738, Corning Life Sciences Inc., Tewksbury, MA, USA) at 37 °C. Then islets were transferred into 1 mL of KRB containing either 2 mM (low) or 17 mM (high) glucose and incubated for an additional 45 min. Afterwards, media samples were collected, and islets were lysed in 1 mL 1X Cell Lysis Buffer (Cell Signaling Technology #9803S, Danvers, MA, USA). Insulin content was measured using an in-house ELISA as previously described [32].

### 3.4. In Silico Studies

The three-dimensional structure of Takeda G-protein-coupled receptor 5 (TGR5) in complex with its known agonist INT-777 was downloaded from the Protein Data Bank (PDB ID: 7CFN), determined by cryo-electron microscopy at a resolution of 3.0 Å. The analysis of the structural and sequence of deposited models has identified five separate protein chains that create a diverse assembly of crystals for TGR5. The five chains are denoted as Chains A (394 AA), B (358 AA), G (58 AA), N (128 AA), and R (330 AA). Because Chain R has been shown to have a direct binding site for INT-777 and because Chain R is representative of all agonists that target TGR5, it was reasonable to direct our docking studies exclusively toward Chain R in order to further evaluate agonist-receptor interactions.

In silico docking studies were conducted using AutoDockVina version 1.2.3 (The Scripps Research Institute, La Jolla, San Diego, CA, USA). The ligands and receptors were optimized, and grid boxes were created. By arranging the grid coordinates (X, Y, and Z) around the active site of the receptor using the co-crystal ligand, a grid box was generated. A grid size of 40 × 40 × 40 xyz points was specified for the target area, with the grid center located at dimensions (x, y, z): (96.30, 121.46, 109.69) [16].

### 3.5. Quantification and Statistical Analysis

Data were acquired using software linked to instruments and plotted in Microsoft Excel or GraphPad Prism version 10. Statistical analyses were performed using GraphPad Prism and Microsoft Excel software. Data were first tested for normality, and then either parametric or non-parametric tests were performed. Statistical significance was assessed using Welch’s *t*-tests, or one-way ANOVA followed by Dunnett’s multiple comparisons tests, wherever appropriate, based on normality tests.

## 4. Conclusions

This study offers a strategy for the design and evaluation of novel TGR5 agonists. We build upon previous attempts at TGR5 agonist drug development programs to establish a workflow that tests for toxicity, safety, side-effect profile, increase in receptor expression, and, for the first time, the ability of compounds to induce TGR5 G protein-biased agonism without recruitment of β-arrestins. Structure–activity relationship analysis exploration reveals beneficial compound–receptor interaction motifs that could lead to improved TGR5 agonists for diabetes and potentially other diseases. We identified novel TGR5 agonists that can produce sustained increases in GLP-1 and prolonged antidiabetic properties. Overall, this study provides an interdisciplinary framework for the development of agonists that integrate in silico, in vitro, and in vivo systems, which can be used for a variety of GPCR-targeted therapeutics, while contributing to the biology of TGR5 agonism and expression in the gut.

## Figures and Tables

**Table 1:** EC_50_ for indicated compounds.

Compound		hTGR5 EC_50_ (µM)
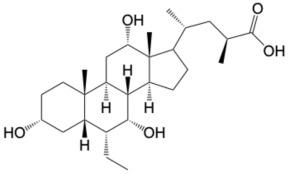		
INT-777		1.34
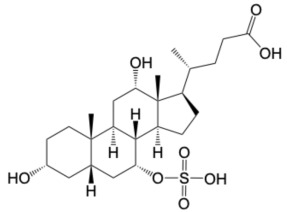		
CA7S		0.01
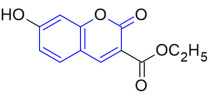		
RSH1		0.01
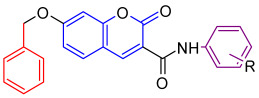		
RSH2		0.02
**Compound**	**R**	**hTGR5 EC_50_ (µM)**
RSH-3	4-Me	0.16
RSH-4	4-Cl	2.58
RSH-5	4-Br	1.94
RSH-6	4-OH	Unstable
RSH-7	3,4-di-Me	Unstable
RSH-8	H	>100
RSH-23	2-Me	6.15
RSH-24	3-Me	Unstable
RSH-25	3-Cl	Unstable
RSH-26	2-OMe	1.64
RSH-27	4-OMe	Unstable
RSH-28	2,4-di-Me	Unstable
RSH-29	2,5-di-Me	Unstable
RSH-30	2,6-di-Me	0.07
RSH-31	2-Cl	0.02
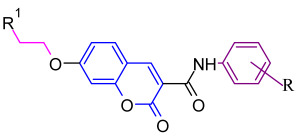		
R^1^ = 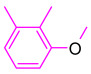		
RSH-13	4-OH	>100
RSH-14	3,4-di-Me	2.18
RSH-15	4-Me	Unstable
RSH-16	4-Cl	Unstable
RSH-17	H	Unstable
R^1^ = 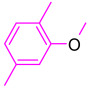		
RSH-18	H	>100
RSH-19	4-Me	1.42
RSH-20	4-Cl	Unstable
RSH-21	4-OH	>100
RSH-22	3,4-di-Me	Unstable

**Table 2:** Structure–activity relationship (SAR) analysis of designed TGR5 agonists.

Compound	Ring A Substitution	Electronic Nature (Ring A)	Ring B Substitution	Key Structural Feature	Relative TGR5 Expression	SAR Interpretation
RSH-14	3,4-Dimethoxy	Strong electron-donating	2,3-Dimethyl phenoxy	Disubstituted electron-donating groups with a hydrophobic substituent	High (~3-fold)	Indicates an optimal balance of electronic donation and hydrophobic interactions within the TGR5 binding pocket
RSH-30	2,6-Dimethoxy	Strong electron-donating	Benzyl	Disubstituted electron-donating groups with a bulky hydrophobic moiety	High (~3-fold)	Bulky hydrophobic substitution likely enhances π–π stacking and hydrophobic interactions
RSH-7	Mono-substituted methoxy/methyl	Moderate electron-donating	Phenyl derivative	Single electron-donating substituent	Low	A single substituent appears insufficient to promote strong receptor interactions
RSH-22	Electron-withdrawing substituent	Electron-withdrawing	Phenyl derivative	Presence of an electron-withdrawing group on Ring A	Very low	Electron-withdrawing effects may reduce the electronic stabilization required for receptor binding
RSH-23	Mixed substituents	Weak electron-donating/withdrawing	Phenyl derivative	Non-optimal substitution pattern	Low	Substituent combination likely leads to poor alignment within the TGR5 binding site

## Data Availability

Data not provided in the manuscript or Supplementary information are available from the authors on request.

## Supplementary Materials

The following supporting information can be downloaded at: https://www.mdpi.com/article/10.3390/molecules31071093/s1, Figure S1: ^1^H NMR spectra of RSH-1; Figure S2: ^1^H NMR spectra of RSH-2; Figure S3: ^1^H NMR spectra of RSH-3; Figure S4: ^13^C NMR spectra of RSH-3; Figure S5: HR-MS spectra of RSH-3; Figure S6: ^1^H NMR spectra of RSH-4; Figure S7: HR MS spectra of RSH-4; Figure S8: ^1^H NMR Spectra of RSH-5; Figure S9: HR MS spectra of RSH-5; Figure S10: ^1^H NMR Spectra of RSH-6; Figure S11: HR MS spectra of RSH-6; Figure S12: ^1^H NMR spectra of RSH-7; Figure S13: ^13^C NMR spectra of RSH-7; Figure S14: HR-MS spectra of RSH-7; Figure S15: ^1^H NMR spectra of RSH-8; Figure S16: ^13^C NMR spectra of RSH-8; Figure S17: HR-MS spectra of RSH-8; Figure S18: ^1^H NMR spectra of RSH-10; Figure S19: ^13^C NMR spectra of RSH-10; Figure S20: HR-MS spectra of RSH-10; Figure S21: ^1^H NMR spectra of RSH-11; Figure S22: ^1^H NMR spectra of RSH-12; Figure S23: ^1^H NMR spectra of RSH-13; Figure S24: ^13^C NMR spectra of RSH-13; Figure S25: ^1^H NMR spectra of RSH-14; Figure S26: ^13^C NMR spectra of RSH-14; Figure S27: HR-MS spectra of RSH-14; Figure S28: ^1^H NMR spectra of RSH-15; Figure S29: ^13^C NMR spectra of RSH-15; Figure S30: HR-MS spectra of RSH-15; Figure S31^: 1^H NMR spectra of RSH-16; Figure S32: HR-MS spectra of RSH-16; Figure S33: ^1^H NMR spectra of RSH-17; Figure S34: ^13^C NMR spectra of RSH-17; Figure S35: HR-MS spectra of RSH-17; Figure S36: ^1^H NMR spectra of RSH-18; Figure S37: ^1^H NMR spectra of RSH-19; Figure S38: ^1^H NMR spectra of RSH-21; Figure S39: HR MS spectra of RSH-21; Figure S40: ^1^H NMR spectra of RSH-24; Figure S41: ^1^H NMR spectra of RSH-26; Figure S42: ^1^H NMR spectra of RSH-27; Figure S43: ^1^H NMR spectra of RSH-28; Figure S44: ^13^C NMR spectra of RSH-28; Figure S45: HR-MS spectra of RSH-28; Figure S46: ^1^H NMR spectra of RSH-29; Figure S47: ^1^H NMR spectra of RSH-30; Figure S48: ^1^H NMR spectra of RSH-31; Figure S49: Western Blot images.
