# Supplementary material for: Rational Design and Evaluation of Novel TGR5 Agonists for Diabetes
Source: Molecules. 2026 Mar 26;31(7):1093. doi: 10.3390/molecules31071093 (PMC13075183; doi:10.3390/molecules31071093)

Rational design and evaluation of novel TGR5 agonists for diabetes

Rachana S. Bhimanwar [1,2,3], Zachary Detwiler [4], Jinge G. Zhu [5], Samuel T. Saghafi [6], Carolyn A. Winder [5], Dawn Belt Davis [6,7], Amit Mittal [3], Vikas Sharma [3], David A. Harris [5,8,*] and Snehal N. Chaudhari [9,*]

[1]Department of Pharmaceutical Chemistry, Dr. D. Y. Patil Institute of Pharmaceutical Sciences and Research, Pune 411018, India; rachana.bhimanwar@dypvp.edu.in
[2]Department of Pharmaceutical Chemistry, School of Pharmacy and Research, Dnyaan Prasad Global University, Pune 411018, India
[3]Department of Pharmaceutical Chemistry, School of Pharmaceutical Sciences, Lovely Professional University, Phagwara 144411, India; amitmittal77@yahoo.com (A.M.); vikaspharma26@gmail.com (V.S.)
[4]Wisconsin Institute for Discovery, University of Wisconsin-Madison, Madison, WI 53706, USA; zdetwiler@wisc.edu
[5]Wisconsin Surgical Laboratory in Metabolism (WiSLiM), Department of Surgery, University of Wiscon-sin-Madison, Madison, WI 53705, USA; jgzhu@wisc.edu (G.Z.); cwinder@wisc.edu (C.A.W.)
[6]Division of Endocrinology, Diabetes, and Metabolism, Department of Medicine, University of Wiscon-sin-Madison, Madison, WI 53705 ,USA; ssaghafi@medicine.wisc.edu (S.T.S.); dbd@medicine.wisc.edu (D.B.D.)
[7]Geriatrics Research Education and Clinical Center, William S. Middleton Memorial Veterans Hospital,
Madison, WI 53705, USA
[8]William S. Middleton Memorial Veterans Hospital, Madison, WI 53705, USA
[9]Department of Biochemistry, University of Wisconsin-Madison, Madison, WI 53706, USA

*Co-corresponding authors:

David A. Harris MD
1111 Highland Avenue
WIMR 5131
Madison WI 53705
daharris6@wisc.edu

and

Snehal N. Chaudhari PhD
University of Wisconsin-Madison
433 Babcock Drive, Madison, WI 53706
snchaudhari@wisc.edu

Content

Copies of ^{1} H NMR, ^{13}C NMR, and HRMS spectrum of the final compounds--------S5-S53

Figure S1: ^{1}H NMR spectra of RSH-1 --S5

Figure S2: ^{1}H NMR spectra of RSH-2--S6

Figure S3: ^{1}H NMR spectra of RSH-3--S7

Figure S4: ^{13}C NMR spectra of RSH-3--S8

Figure S5: HR-MS spectra of RSH-3-- S9

Figure S6: ^{1}H NMR spectra of RSH-4-- S10

Figure S7: HR MS spectra of RSH-4--S11

Figure S8: ^{1}H NMR Spectra of RSH-5-- S12

Figure S9: HR MS spectra of RSH-5--S13

Figure S10: ^{1}H NMR Spectra of RSH-6--S14

Figure S11: HR MS spectra of RSH-6--S15

Figure S12: ^{1}H NMR spectra of RSH-7--S16

Figure S13: ^{13}C NMR spectra of RSH-7-- S17

Figure S14: HR-MS spectra of RSH-7--S18

Figure S15: ^{1}H NMR spectra of RSH-8--S19

Figure S16: ^{13}C NMR spectra of RSH-8--S20

Figure S17: HR-MS spectra of RSH-8--S21

Figure S18: ^{1}H NMR spectra of RSH-10-- S22

Figure S19: ^{13}C NMR spectra of RSH-10--S23

Figure S20: HR-MS spectra of RSH-10--S24

Figure S21: ^{1}H NMR spectra of RSH-11--S25

Figure S22: ^{1}H NMR spectra of RSH-12--- S26

Figure S23: ^{1}H NMR spectra of RSH-13--S27

Figure S24: ^{13}C NMR spectra of RSH-13-- S28

Figure S25: ^{1}H NMR spectra of RSH-14--S29

Figure S26: ^{13}C NMR spectra of RSH-14-- S30

Figure S27: HR-MS spectra of RSH-14--S31

Figure S28: ^{1}H NMR spectra of RSH-15--S32

Figure S29: ^{13}C NMR spectra of RSH-15---S33

Figure S30: HR-MS spectra of RSH-15--- S34

Figure S31: ^{1}H NMR spectra of RSH-16--- S35

Figure S32: HR-MS spectra of RSH-16--S36

Figure S33: ^{1}H NMR spectra of RSH-17--S37

Figure S34: ^{13}C NMR spectra of RSH-17--S38

Figure S35: HR-MS spectra of RSH-17---S39

Figure S36: ^{1}H NMR spectra of RSH-18---S40

Figure S37: ^{1}H NMR spectra of RSH-19---S41

Figure S38: ^{1}H NMR spectra of RSH-21---S42

Figure S39: HR MS spectra of RSH-21-- S43

Figure S40: ^{1}H NMR spectra of RSH-24---S44

Figure S41: ^{1}H NMR spectra of RSH-26---S45

Figure S42: ^{1}H NMR spectra of RSH-27---S46

Figure S43: ^{1}H NMR spectra of RSH-28---S47

Figure S44: ^{13}C NMR spectra of RSH-28--S48

Figure S45: HR-MS spectra of RSH-28-- S49

Figure S46: ^{1}H NMR spectra of RSH-29--S50

Figure S47: ^{1}H NMR spectra of RSH-30--S51

Figure S48: ^{1}H NMR spectra of RSH-31--S52

Figure S49: Western Blot images--S53-55

Copies of [1] H NMR, ^{13}C NMR, and HRMS spectrum of the final compounds

Figure S1. ^{1}H NMR spectra of **RSH-1**

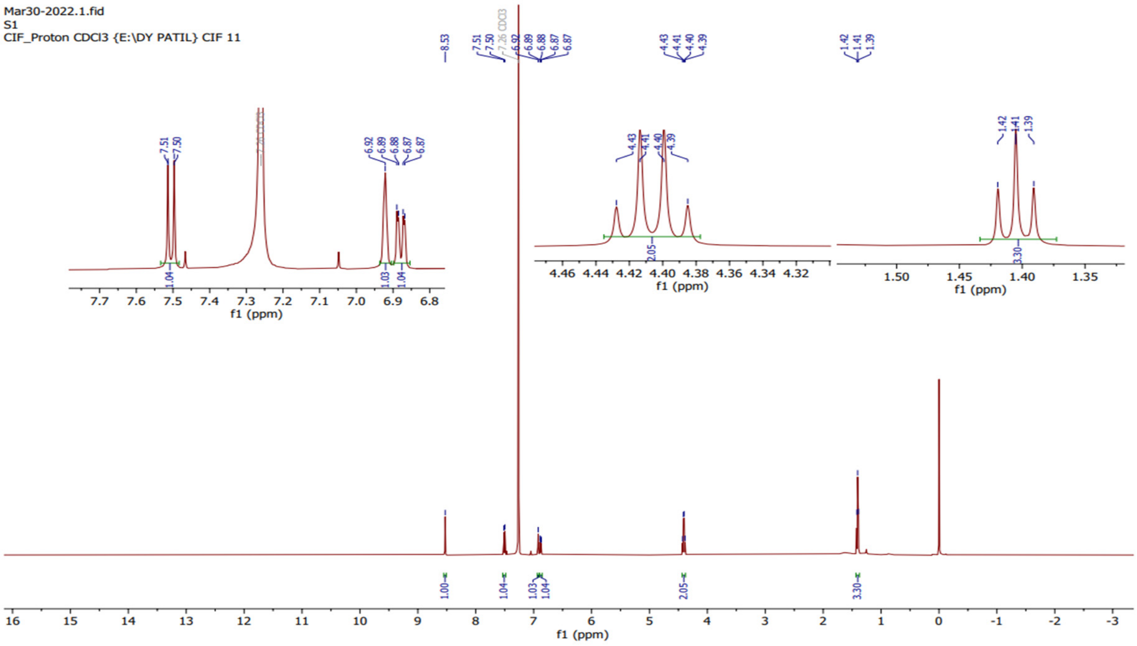

Figure S2. ^{1}H NMR spectra of **RSH-2**

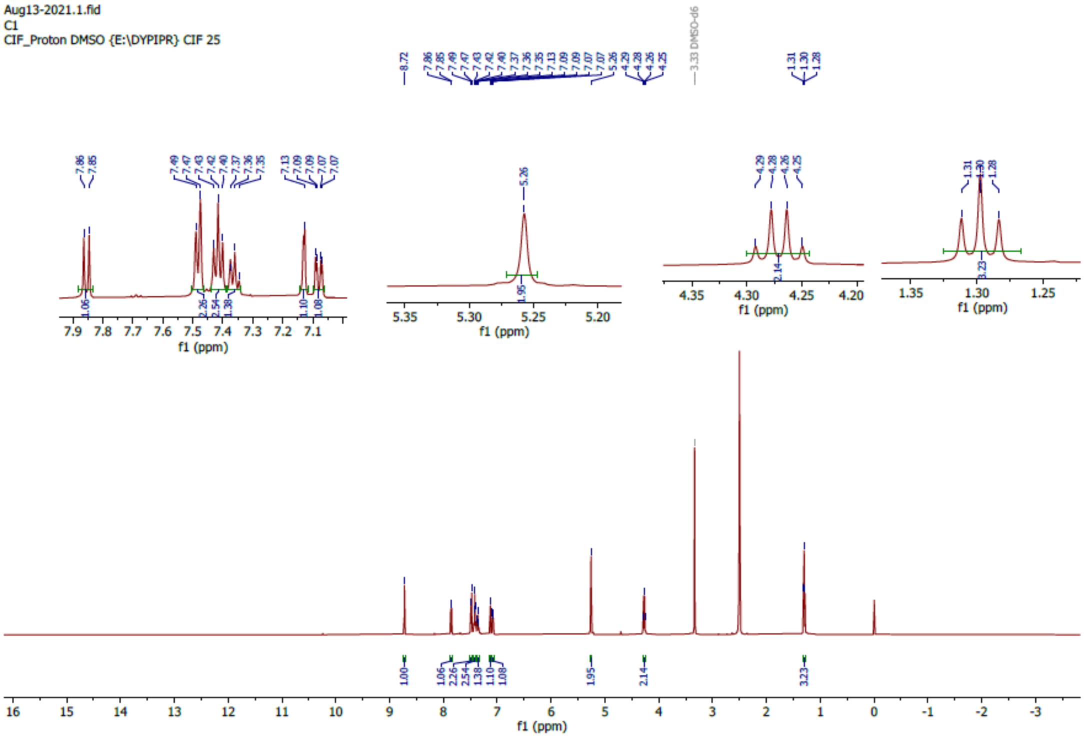

Figure S3. ^{1}H NMR spectra of **RSH-3**

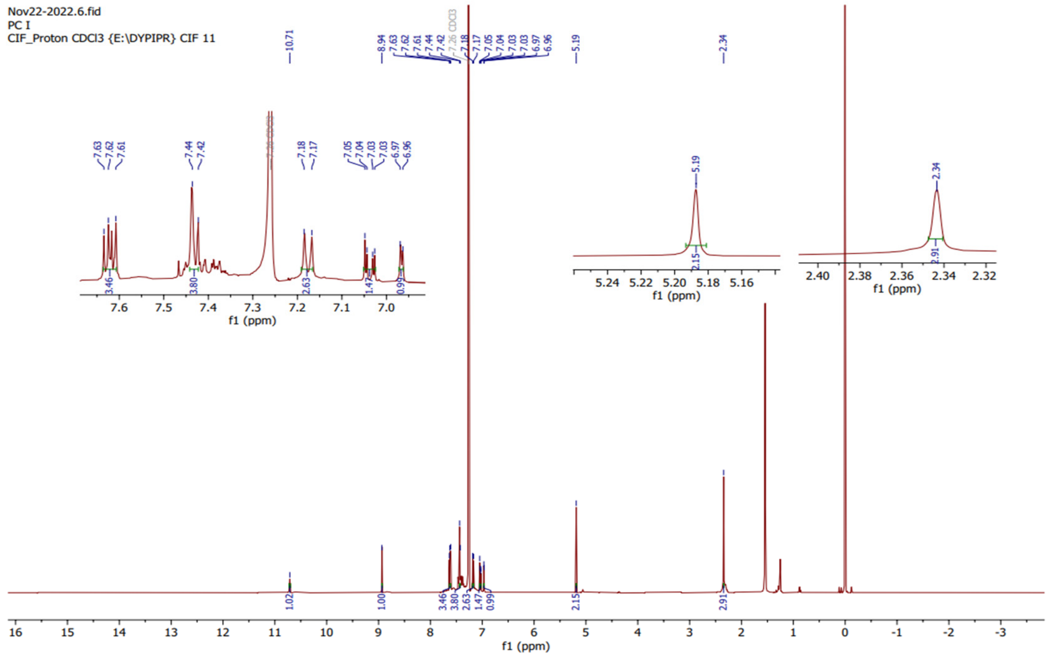

Figure S4. ^{13}C NMR spectra of **RSH-3**

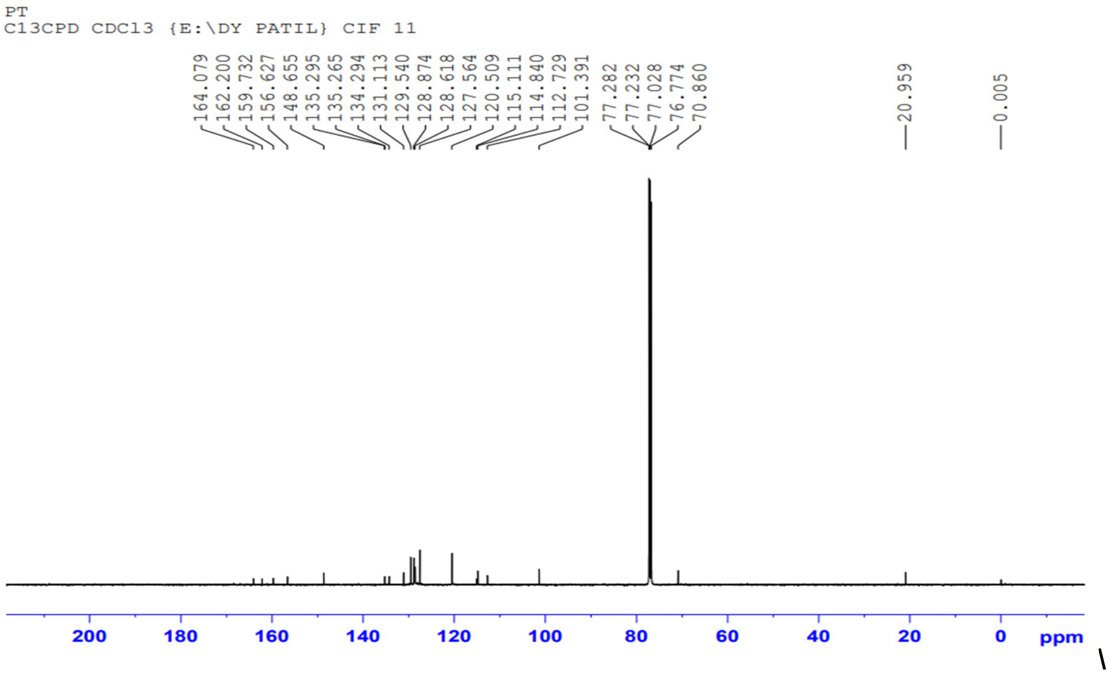

Figure S5. HR-MS spectra of **RSH-3**

Analysis Info

Analysis Name	D:\Data\2023\JULY\SPPU COLLEGE\DYPVP\RACHANA BHIMANWAR\PT_BA6_01_5998.d	Acquisition Date	7/6/2023 12:36:14 PM
Method	dlc_ms20-1200mz_7min_0.120mlflow_90b_mar23.m	Operator	CIF
Sample Name	PT	Instrument	impact HD 1819696.00184

Acquisition Parameter

Source Type	ESI	Ion Polarity	Positive	Set Nebulizer	1.2 Bar
Focus	Active	Set Capillary	4500 V	Set Dry Heater	200 °C
Scan Begin	20 m/z	Set End Plate Offset	-500 V	Set Dry Gas	7.0 l/min
Scan End	1200 m/z	Set Charging Voltage	2000 V	Set Divert Valve	Source
		Set Corona	0 nA	Set APCI Heater	0 °C

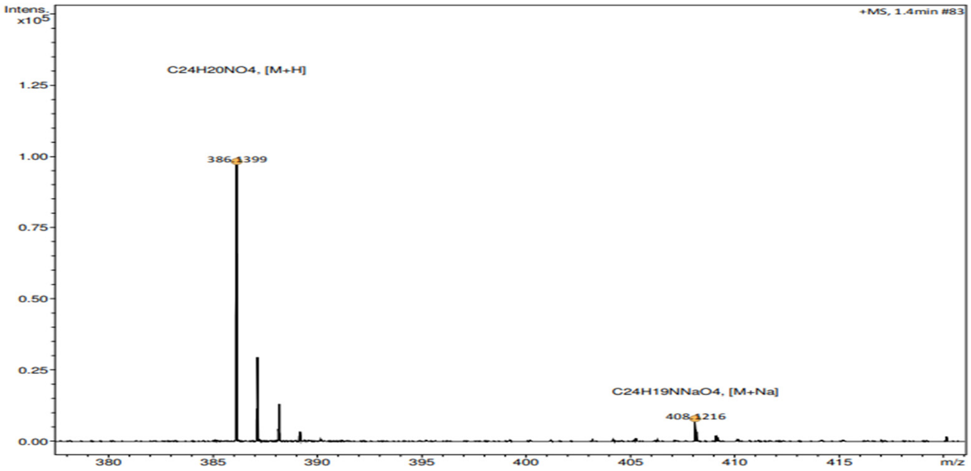

Meas. m/z	#	Ion Formula	Score	m/z	err [mDa]	err [ppm]	mSigma	rdb	e⁻ Conf	N-Rule	Adduct
386.139896	1	C24H20NO4	100.00	386.138685	-1.2	-3.1	21.6	15.5	even	ok	M+H
408.121582	1	C24H19NNaO4	100.00	408.120629	-1.0	-2.3	23.5	15.5	even	ok	M+Na

Figure S6. ^{1}H NMR spectra of **RSH-4**

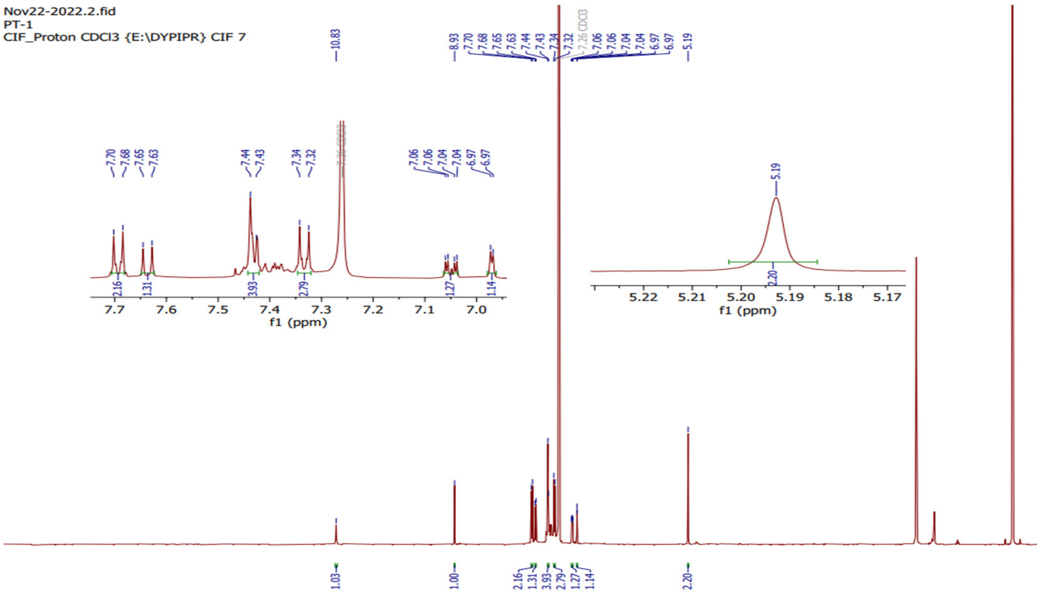

Figure S7. HR MS spectra of **RSH-4**

Acquisition Parameter

Source Type	ESI	Ion Polarity	Positive	Set Nebulizer	1.2 Bar
Focus	Active	Set Capillary	4500 V	Set Dry Heater	200 °C
Scan Begin	20 m/z	Set End Plate Offset	-500 V	Set Dry Gas	7.0 l/min
Scan End	1200 m/z	Set Charging Voltage	2000 V	Set Divert Valve	Source
		Set Corona	0 nA	Set APCI Heater	0 °C

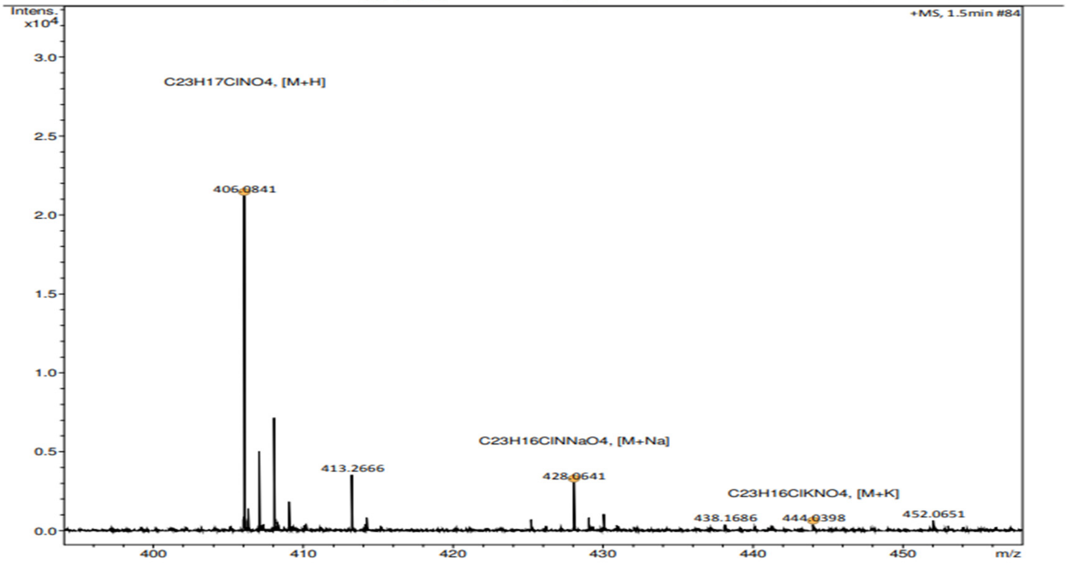

Meas. m/z	#	Ion Formula	Score	m/z	err [mDa]	err [ppm]	mSigma	rdb	e⁻ Conf	N-Rule	Adduct
406.084132	1	C23H17ClNO4	100.00	406.084062	-0.1	-0.2	18.8	15.5	even	ok	M+H
428.064124	1	C23H16ClNNaO4	100.00	428.066006	1.9	4.4	12.7	15.5	even	ok	M+Na

Figure S8. ^{1}H NMR Spectra of **RSH-5**

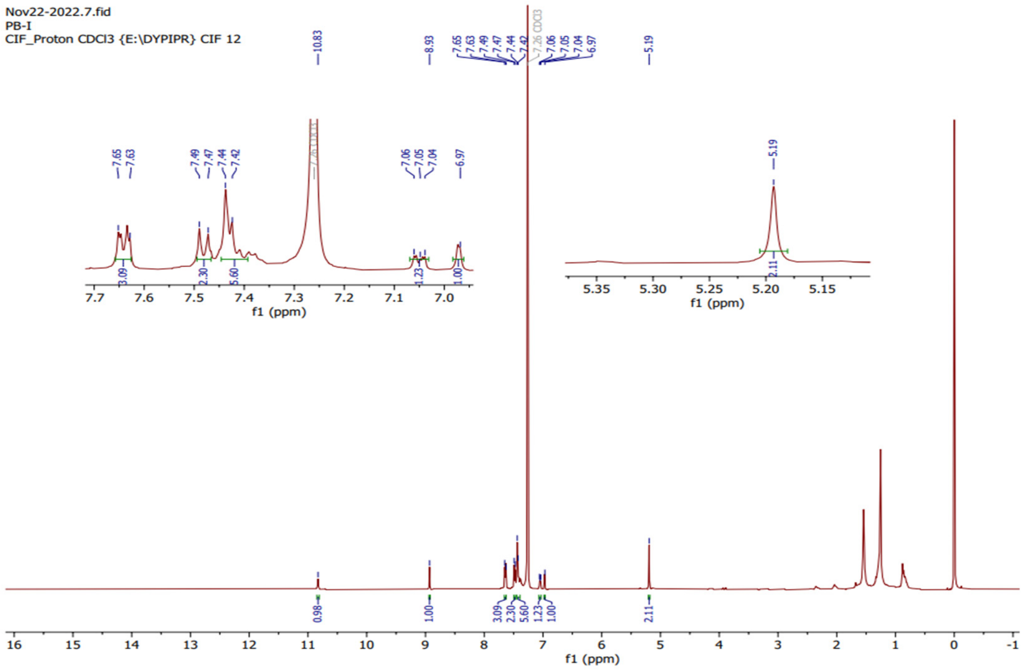

Figure S9. HR MS spectra of **RSH-5**

Acquisition Parameter

Source Type	ESI	Ion Polarity	Positive	Set Nebulizer	0.8 Bar
Focus	Active	Set Capillary	4500 V	Set Dry Heater	200 °C
Scan Begin	20 m/z	Set End Plate Offset	-500 V	Set Dry Gas	7.0 l/min
Scan End	1200 m/z	Set Charging Voltage	2000 V	Set Divert Valve	Source
		Set Corona	0 nA	Set APCI Heater	0 °C

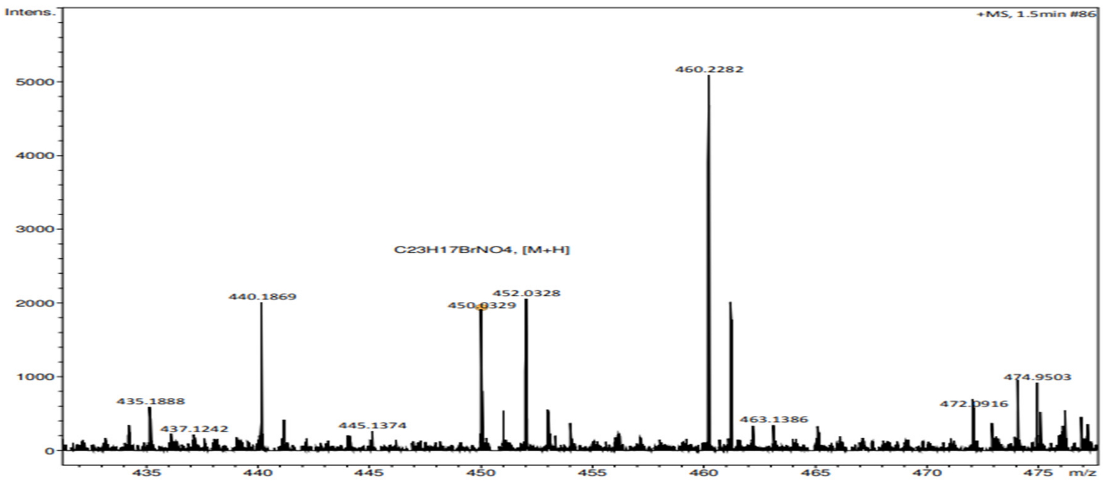

Meas. m/z	#	Ion Formula	Score	m/z	err [mDa]	err [ppm]	mSigma	rdb	e⁻ Conf	N-Rule	Adduct
450.032875	1	C23H17BrNO4	100.00	450.033547	0.7	1.5	62.6	15.5	even	ok	M+H

Figure S10. ^{1}H NMR spectra of **RSH-6**

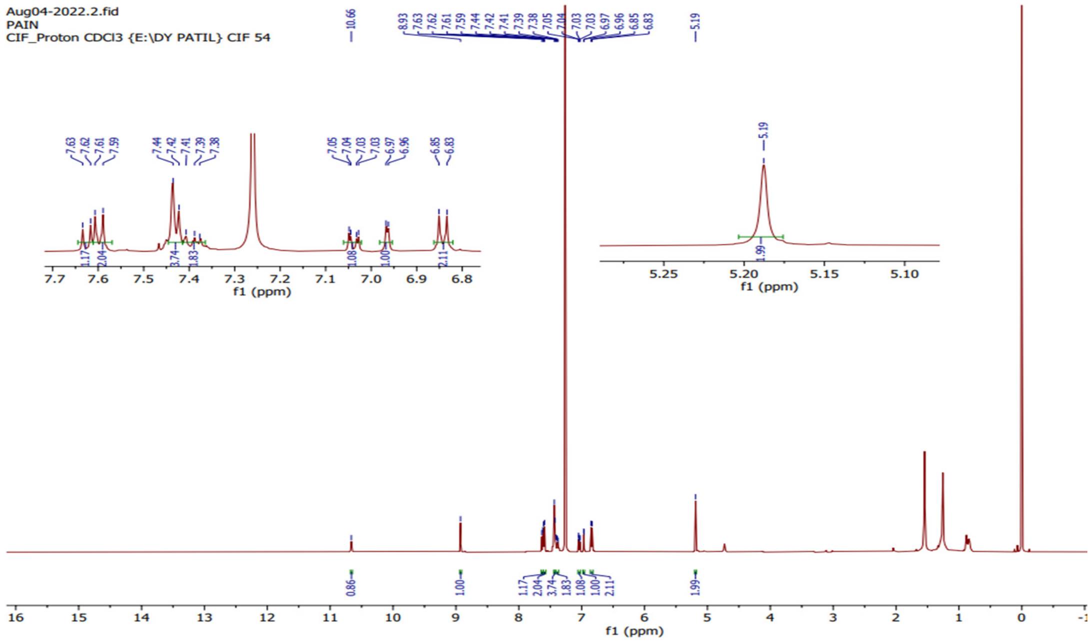

Figure S11. HR MS spectra of **RSH-6**

Acquisition Parameter

Source Type	ESI	Ion Polarity	Positive	Set Nebulizer	1.2 Bar
Focus	Active	Set Capillary	4500 V	Set Dry Heater	200 °C
Scan Begin	20 m/z	Set End Plate Offset	-500 V	Set Dry Gas	7.0 l/min
Scan End	1200 m/z	Set Charging Voltage	2000 V	Set Divert Valve	Source
		Set Corona	0 nA	Set APCI Heater	0 °C

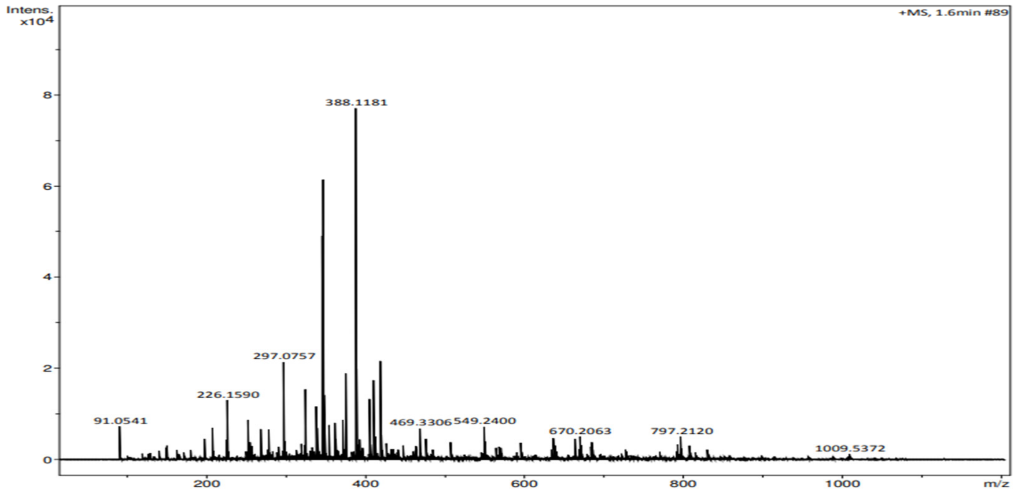

Figure S12. ^{1}H NMR spectra of **RSH-7**

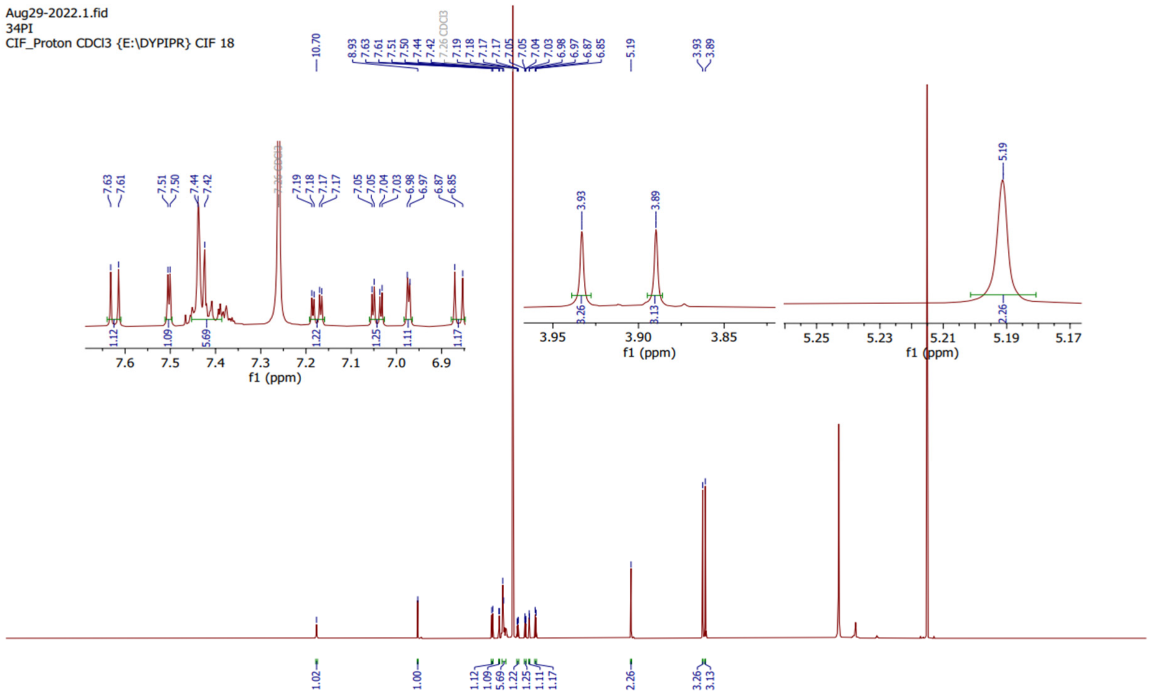

Figure S13. ^{13}C NMR spectra of **RSH-7**

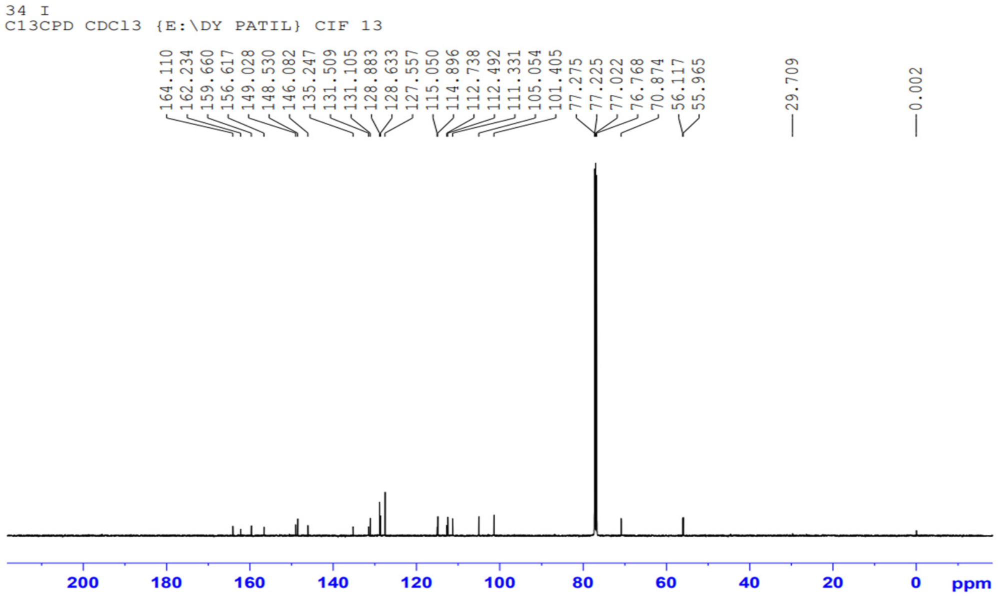

Figure S14. HR-MS spectra of **RSH-7**

Acquisition Parameter

Source Type	ESI	Ion Polarity	Positive	Set Nebulizer	1.2 Bar
Focus	Active	Set Capillary	4500 V	Set Dry Heater	200 °C
Scan Begin	20 m/z	Set End Plate Offset	-500 V	Set Dry Gas	7.0 l/min
Scan End	1200 m/z	Set Charging Voltage	2000 V	Set Divert Valve	Source
		Set Corona	0 nA	Set APCI Heater	0 °C

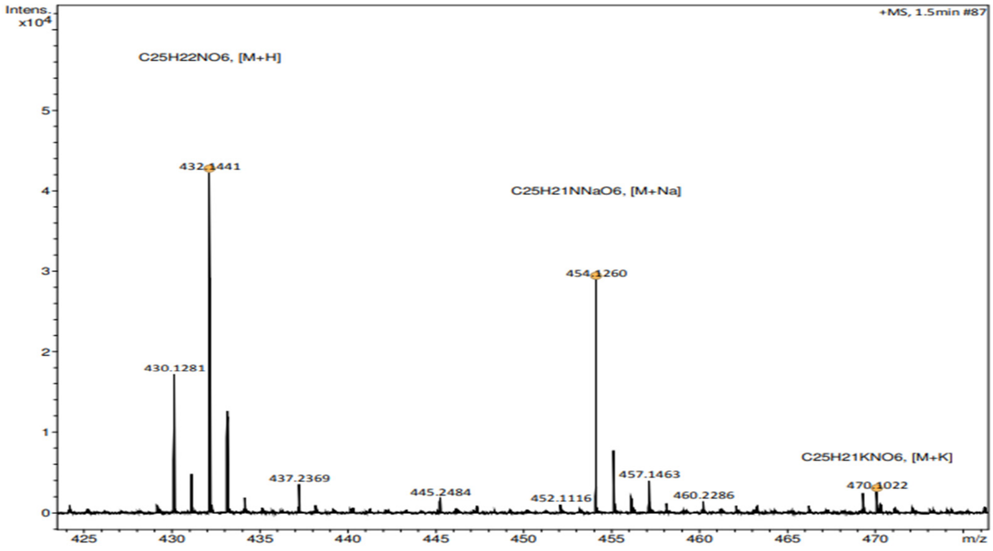

Meas. m/z	#	Ion Formula	Score	m/z	err [mDa]	err [ppm]	mSigma	rdb	e⁻ Conf	N-Rule	Adduct
432.144138	1	C25H22NO6	100.00	432.144164	0.0	0.1	11.6	15.5	even	ok	M+H
454.126038	1	C25H21NNaO6	100.00	454.126108	0.1	0.2	14.4	15.5	even	ok	M+Na

Figure S15. ^{1}HNMR spectra of **RSH-8**

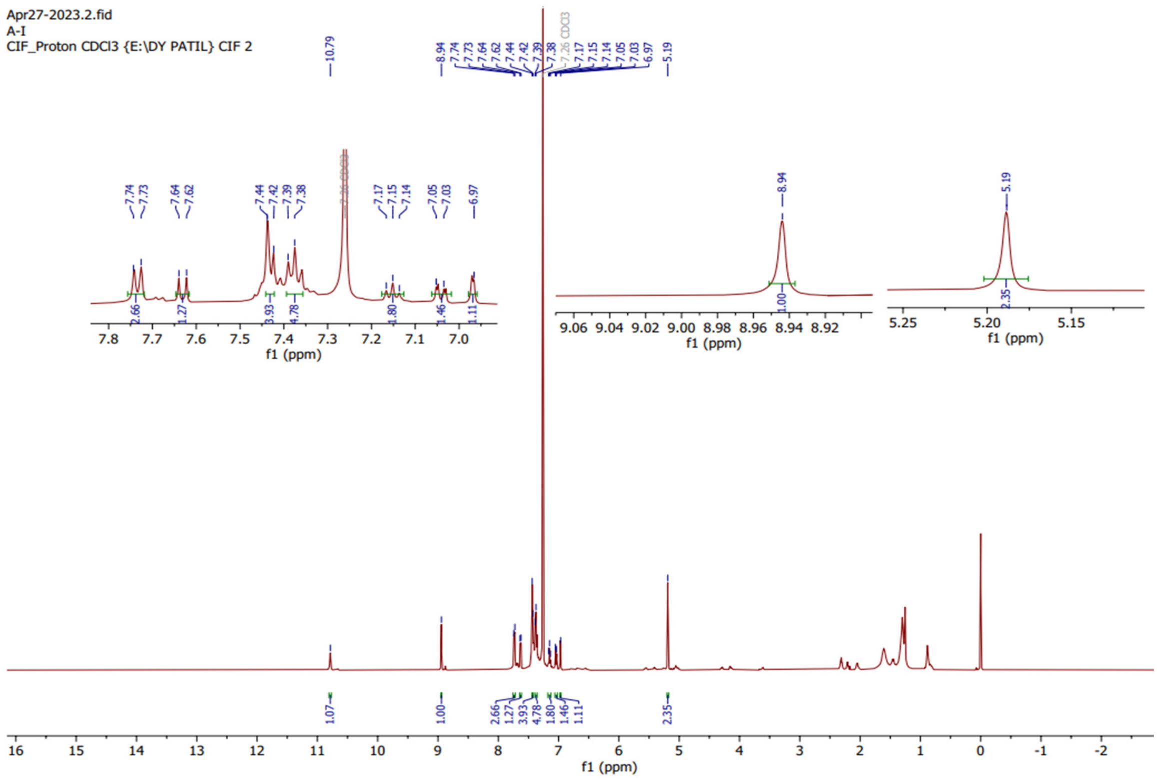

Figure S16. ^{13}C NMR spectra of **RSH-8**

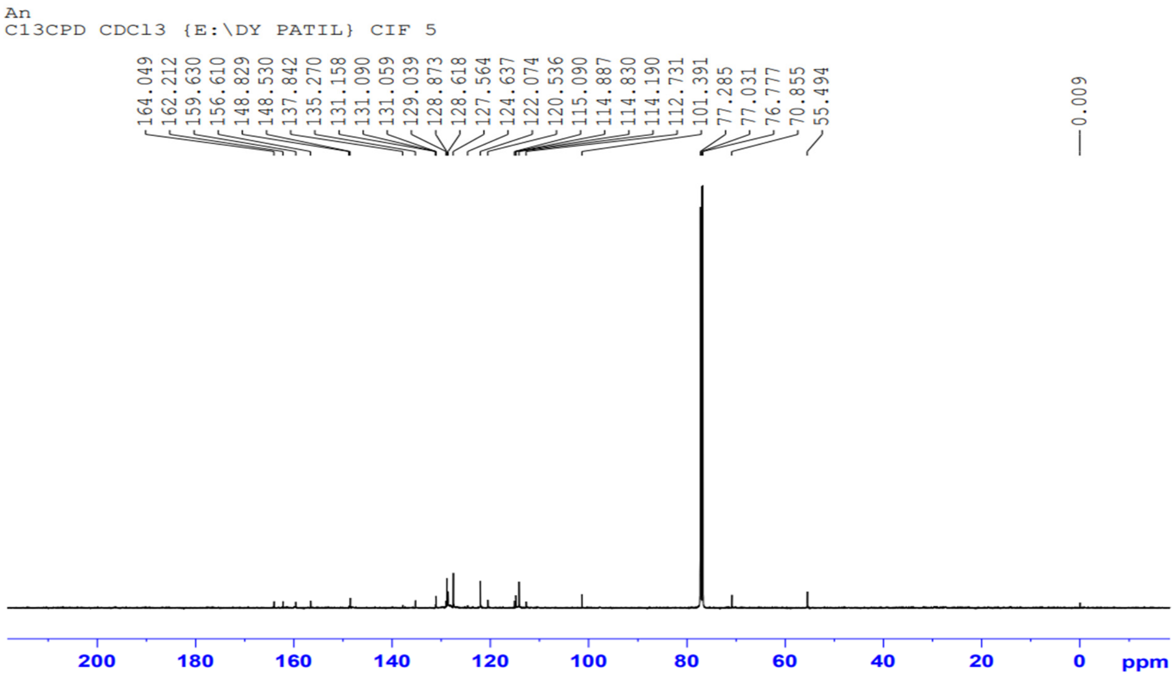

Figure S17. HR MS spectra of **RSH-8**

Acquisition Parameter

Source Type	ESI	Ion Polarity	Positive	Set Nebulizer	0.8 Bar
Focus	Active	Set Capillary	4500 V	Set Dry Heater	200 °C
Scan Begin	20 m/z	Set End Plate Offset	-500 V	Set Dry Gas	7.0 l/min
Scan End	1200 m/z	Set Charging Voltage	2000 V	Set Divert Valve	Source
		Set Corona	0 nA	Set APCI Heater	0 °C

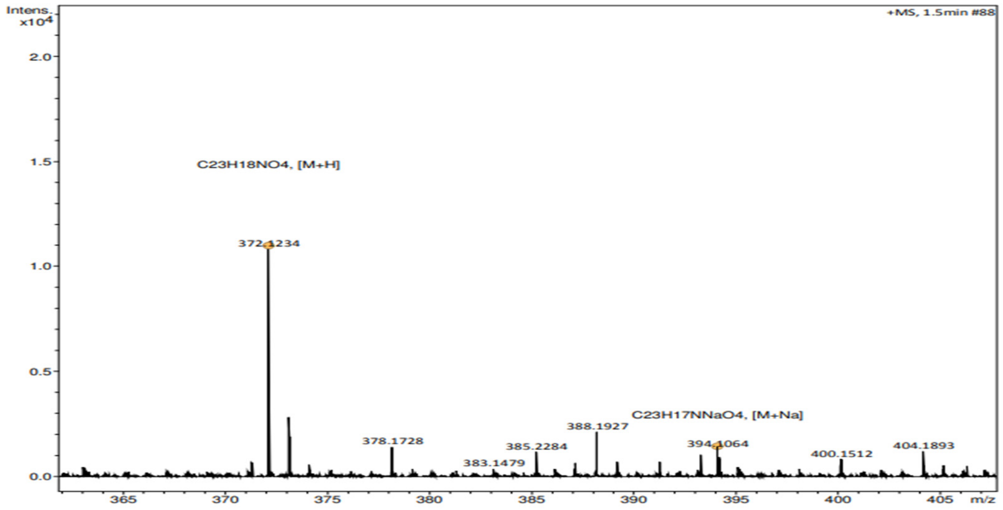

Meas. m/z	#	Ion Formula	Score	m/z	err [mDa]	err [ppm]	mSigma	rdb	e⁻ Conf	N-Rule	Adduct
372.123366	1	C23H18NO4	100.00	372.123034	-0.3	-0.9	7.9	15.5	even	ok	M+H
394.106390	1	C23H17NNaO4	100.00	394.104979	-1.4	-3.6	50.5	15.5	even	ok	M+Na

Figure S18. ^{1}H NMR spectra of **RSH-10**

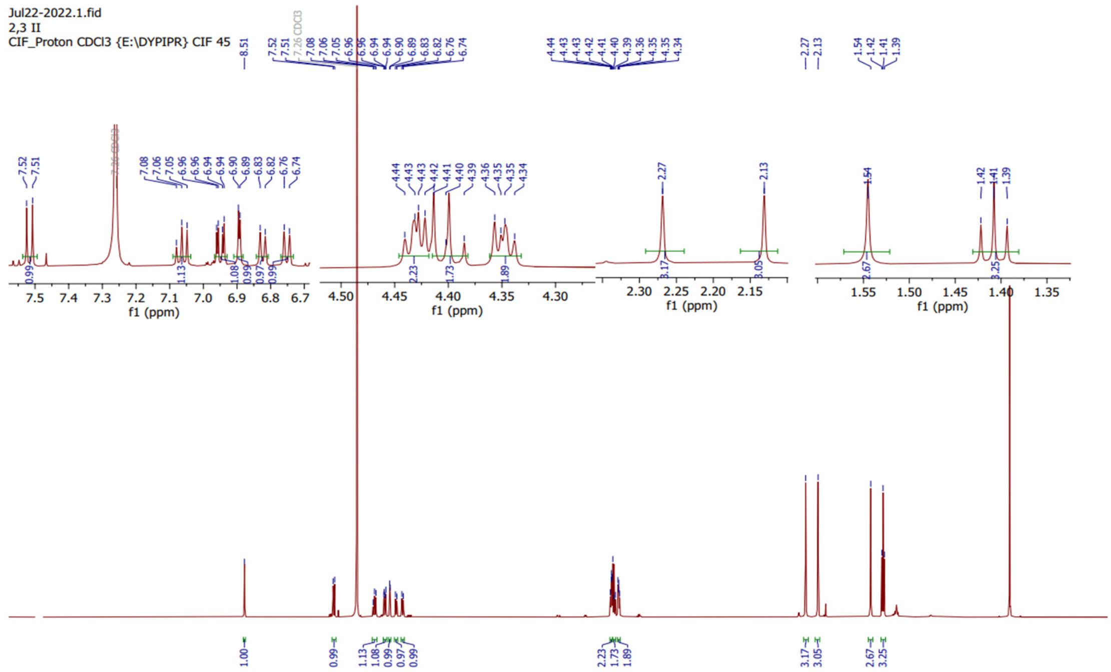

Figure S19. ^{13}C NMR spectra of **RSH-10**

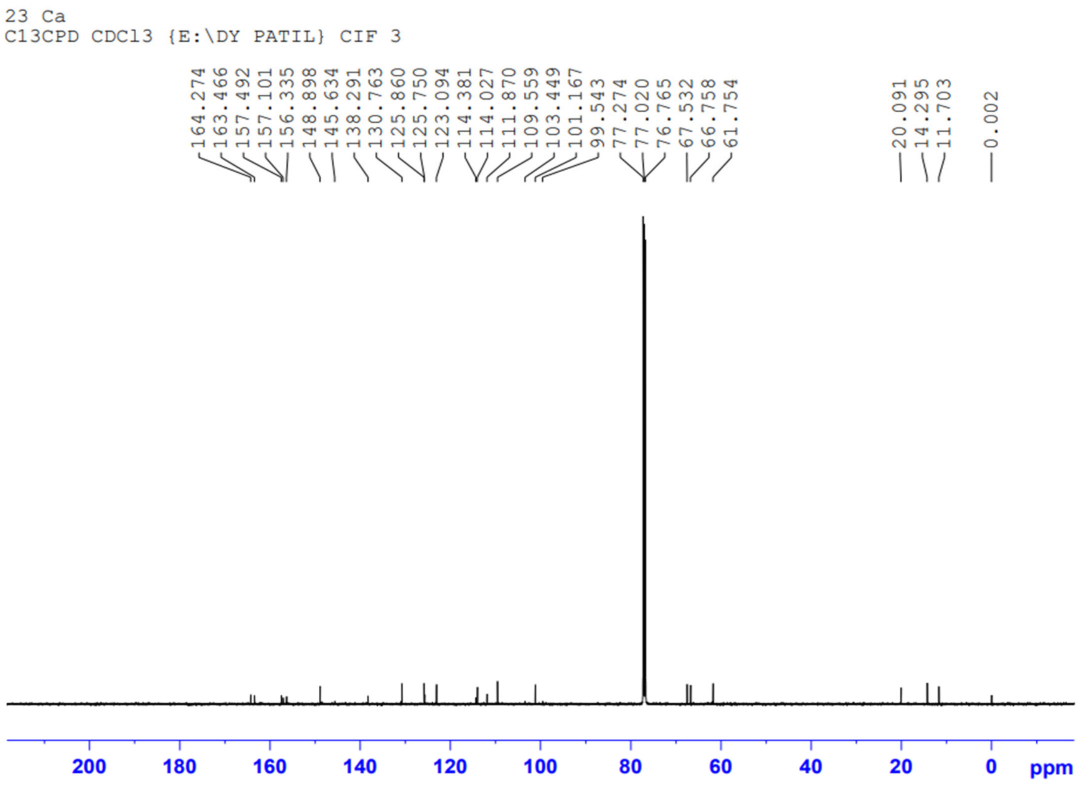

Figure S20. HR-MS spectra of **RSH-10**

Acquisition Parameter

Source Type	ESI	Ion Polarity	Positive	Set Nebulizer	1.7 Bar
Focus	Active	Set Capillary	4500 V	Set Dry Heater	200 °C
Scan Begin	50 m/z	Set End Plate Offset	-500 V	Set Dry Gas	7.0 l/min
Scan End	1200 m/z	Set Charging Voltage	2000 V	Set Divert Valve	Source
		Set Corona	0 nA	Set APCI Heater	0 °C

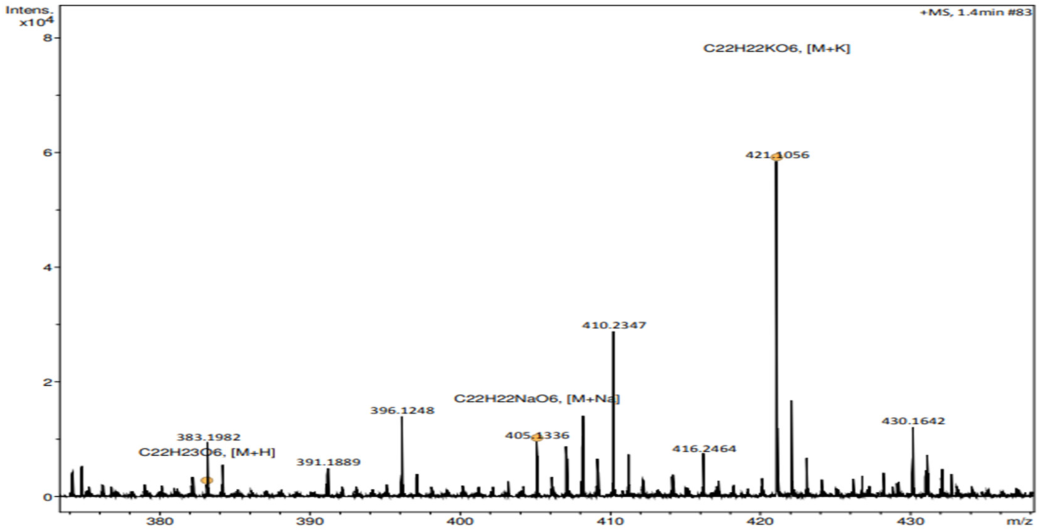

Meas. m/z	#	Ion Formula	Score	m/z	err [mDa]	err [ppm]	mSigma	rdb	e⁻ Conf	N-Rule	Adduct
383.154132	1	C22H23O6	100.00	383.148915	-5.2	-13.6	268.2	11.5	even	ok	M+H
405.133600	1	C22H22NaO6	100.00	405.130859	-2.7	-6.8	79.4	11.5	even	ok	M+Na

Figure S21.^{1}HNMR spectra of **RSH-11**

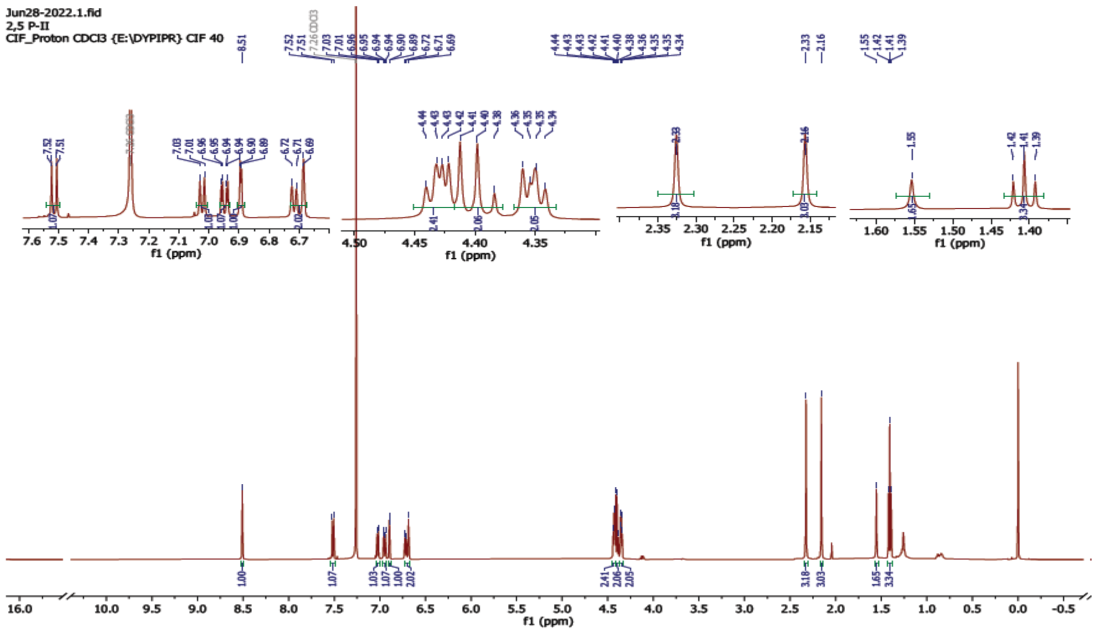

Figure S22.^{1}HNMR spectra of **RSH-12**

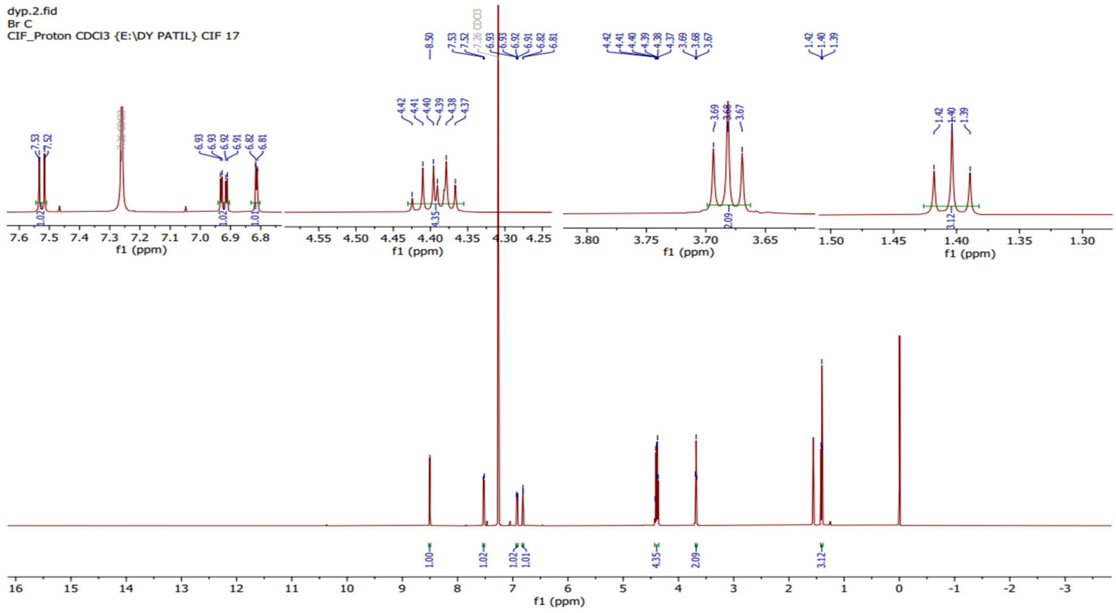

Figure S23.^{1}H NMR spectra of **RSH-13**

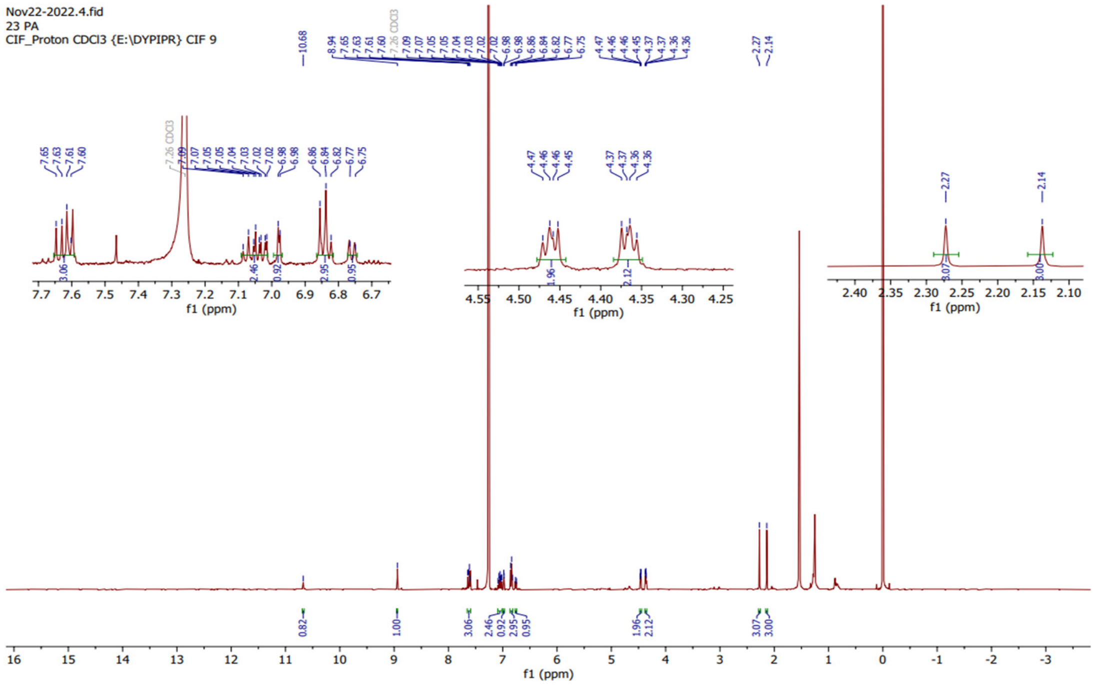

Figure S24.^{13}C NMR spectra of **RSH-13**

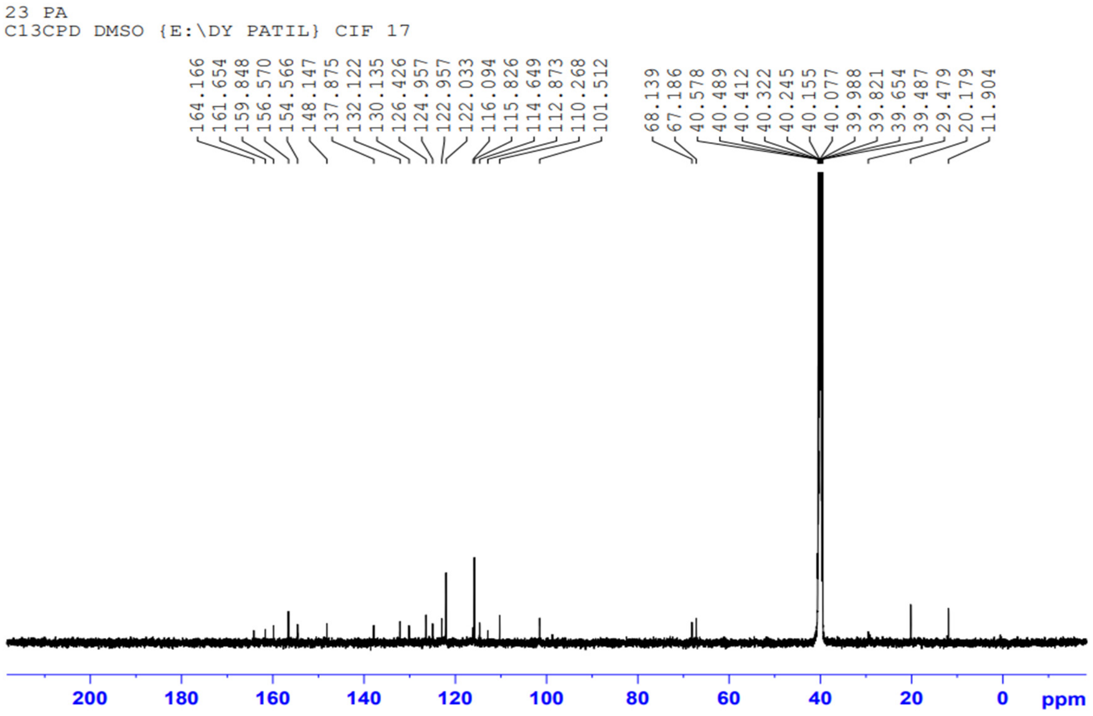

Figure S25.^{1}H NMR spectra of **RSH-14**

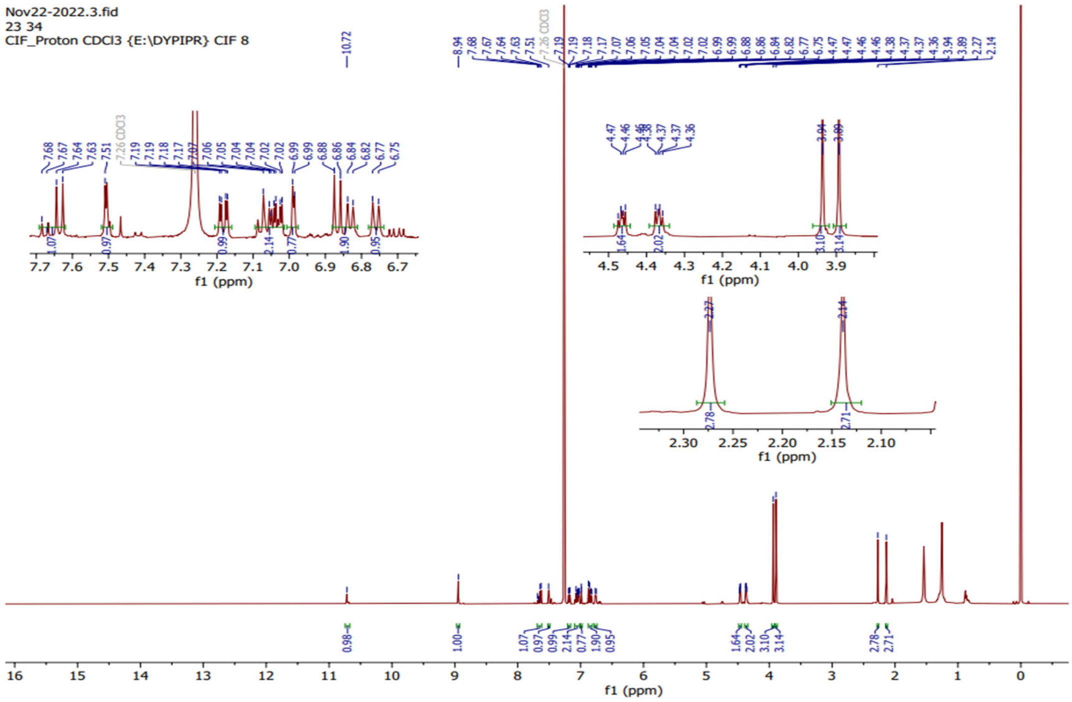

Figure S26.^{13}C NMR spectra of **RSH-14**

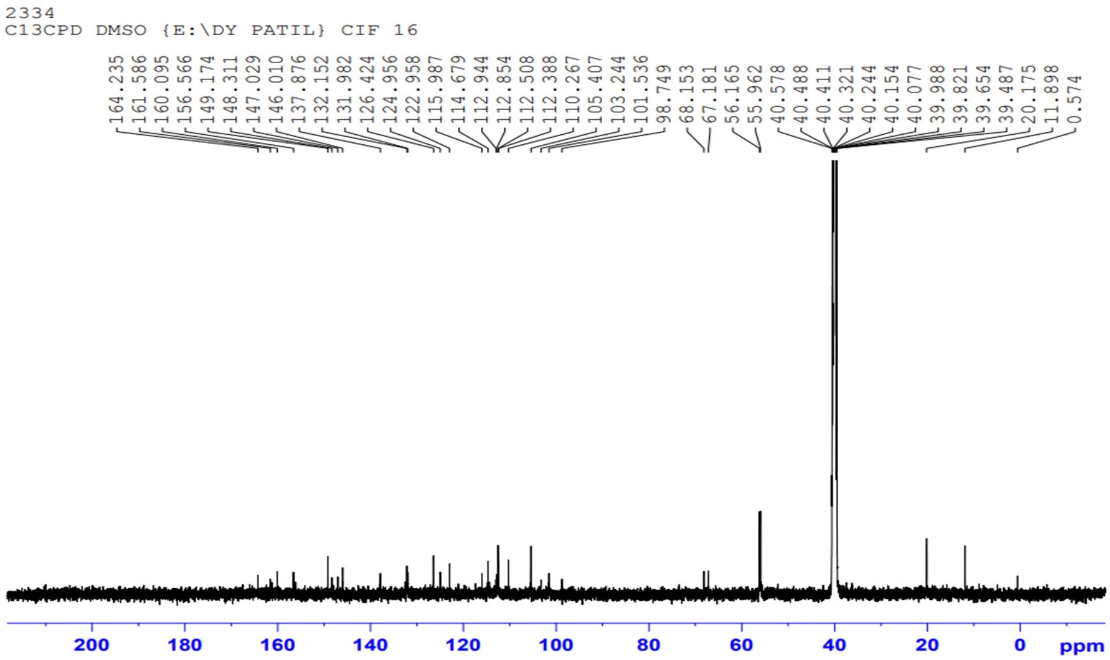

Figure S27.HR MS spectra of **RSH-14**

Acquisition Parameter

Source Type	ESI	Ion Polarity	Positive	Set Nebulizer	0.8 Bar
Focus	Active	Set Capillary	4500 V	Set Dry Heater	200 °C
Scan Begin	20 m/z	Set End Plate Offset	-500 V	Set Dry Gas	7.0 l/min
Scan End	1200 m/z	Set Charging Voltage	2000 V	Set Divert Valve	Source
		Set Corona	0 nA	Set APCI Heater	0 °C

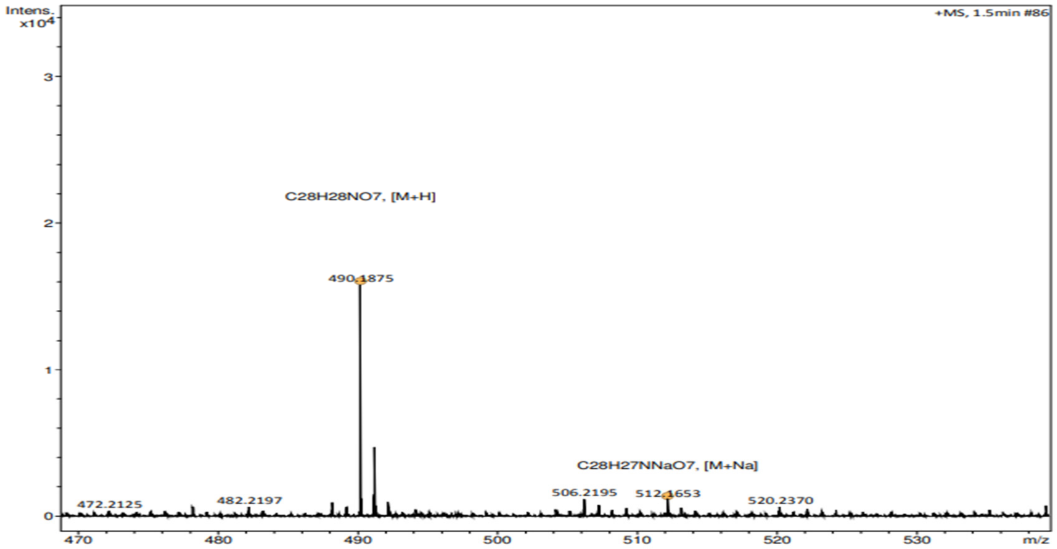

Meas. m/z	#	Ion Formula	Score	m/z	err [mDa]	err [ppm]	mSigma	rdb	e^- Conf	N-Rule	Adduct
490.187450	1	C28H28NO7	100.00	490.186029	-1.4	-2.9	8.2	15.5	even	ok	M+H
512.165280	1	C28H27NNaO7	100.00	512.167973	2.7	5.3	139.4	15.5	even	ok	M+Na

Figure S28.^{1}H NMR spectra of **RSH-15**

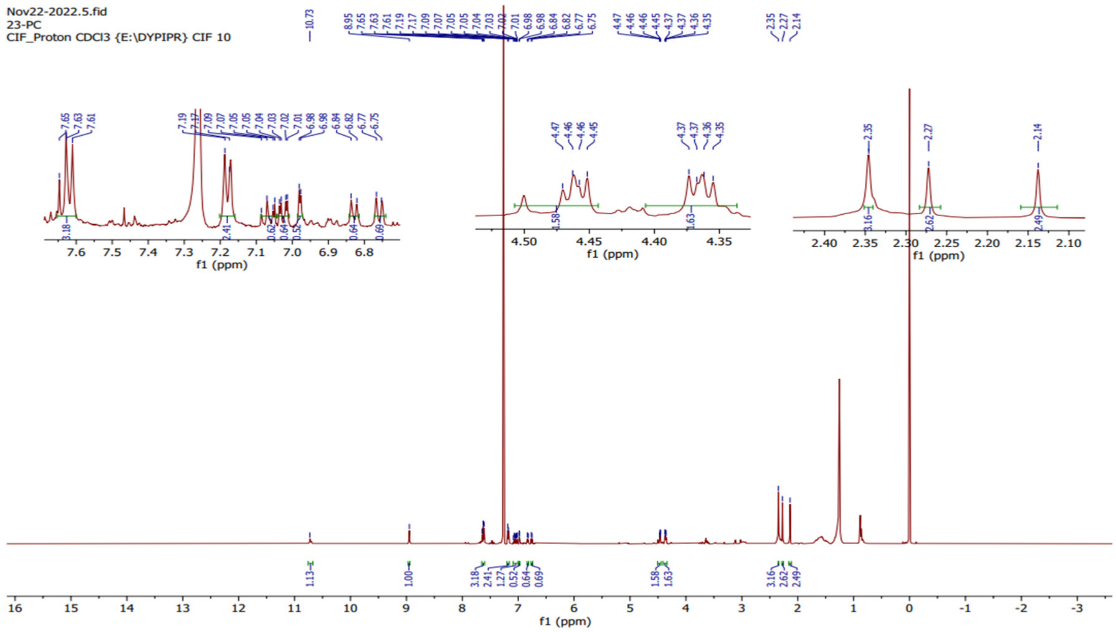

Figure S29.^{13}C NMR spectra of **RSH-15**

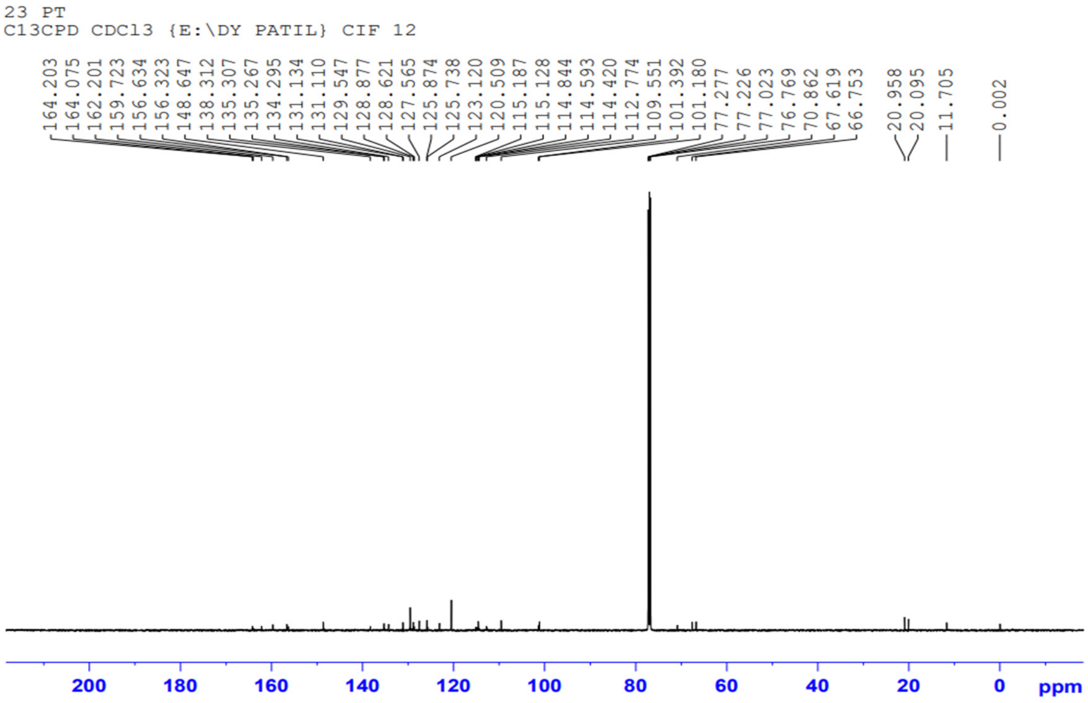

Figure S30. HR MS spectra of **RSH-15**

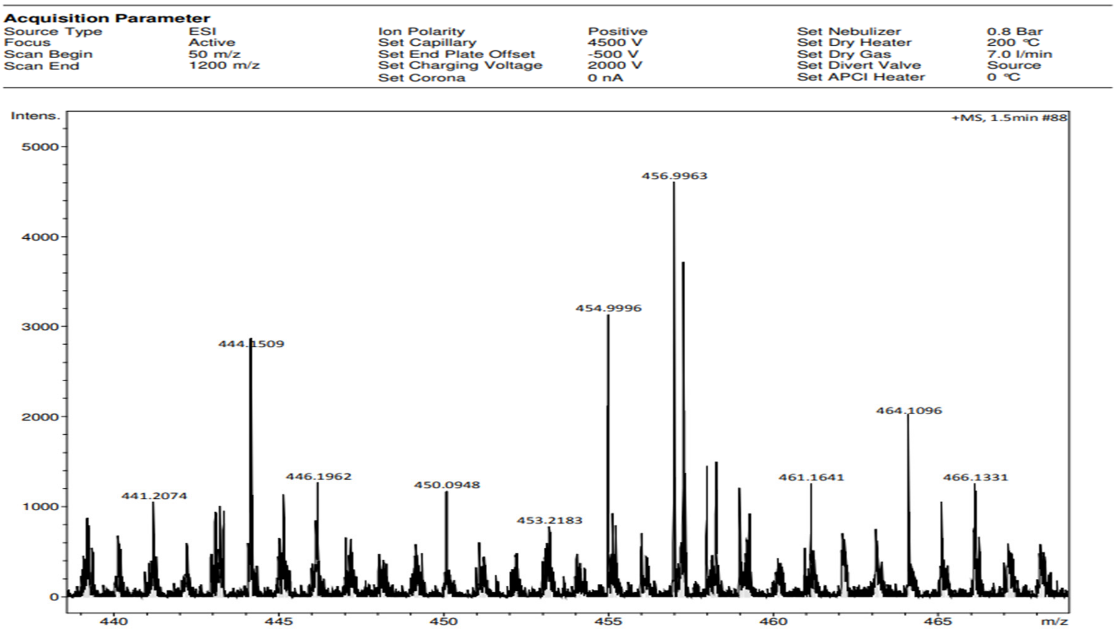

Figure S31. ^{1}H NMR spectra of **RSH-16**

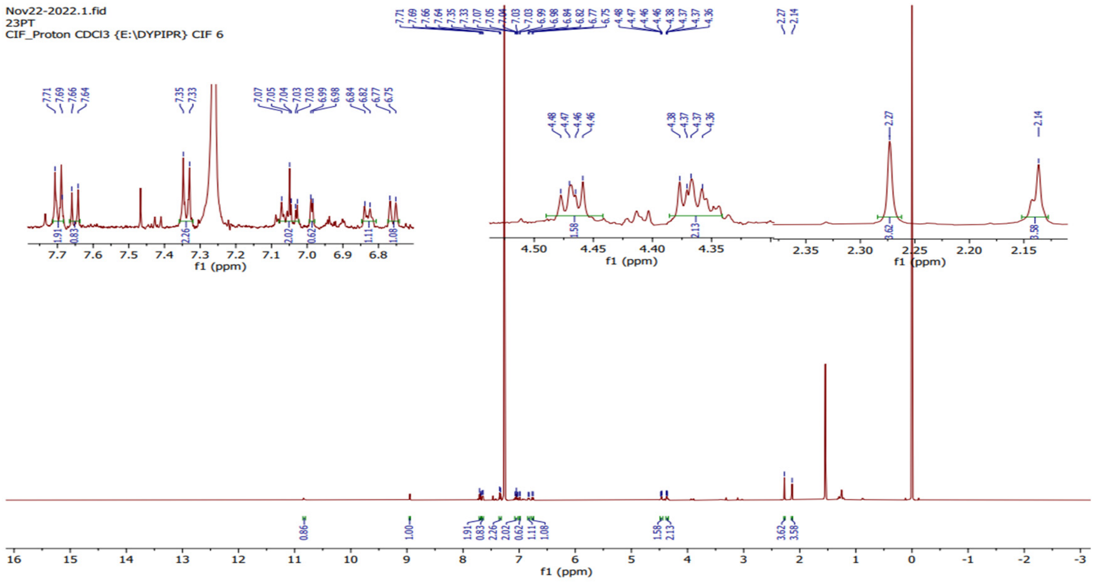

Figure S32. HR MS spectra of **RSH-16**

Acquisition Parameter

Source Type	ESI	Ion Polarity	Positive	Set Nebulizer	0.8 Bar
Focus	Active	Set Capillary	4500 V	Set Dry Heater	200 °C
Scan Begin	50 m/z	Set End Plate Offset	-500 V	Set Dry Gas	7.0 l/min
Scan End	1200 m/z	Set Charging Voltage	2000 V	Set Divert Valve	Source
		Set Corona	0 nA	Set APCI Heater	0 °C

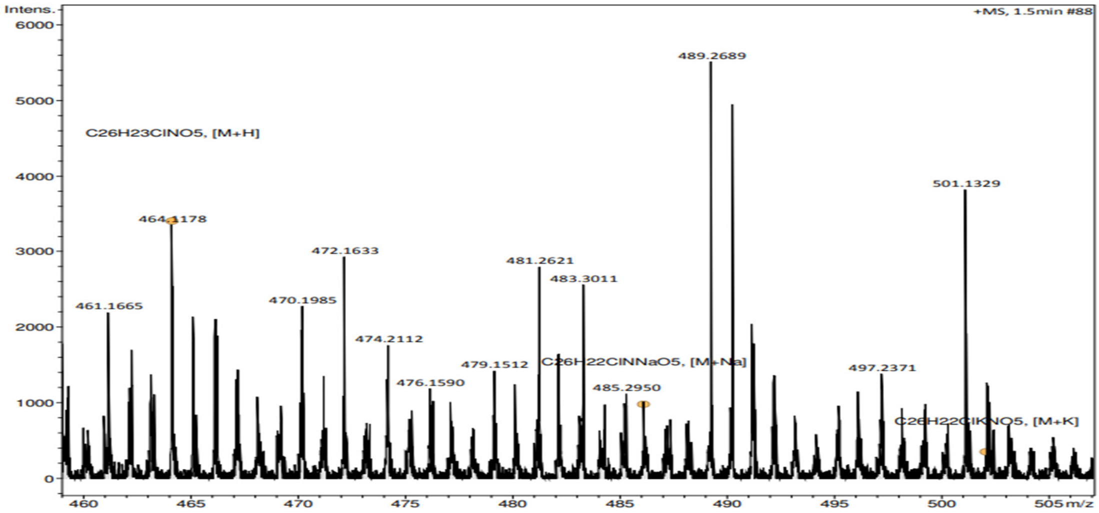

Meas. m/z	#	Ion Formula	Score	m/z	err [mDa]	err [ppm]	mSigma	rdb	e⁻ Conf	N-Rule	Adduct
464.117838	1	C26H23ClNO5	100.00	464.125927	8.1	17.4	261.6	15.5	even	ok	M+H
486.110934	1	C26H22ClNNaO5	100.00	486.107871	-3.1	-6.3	288.9	15.5	even	ok	M+Na
502.077333	1	C26H22ClKNO5	100.00	502.081809	4.5	8.9	524.0	15.5	even	ok	M+K

Figure S33. ^{1}HNMR spectra of **RSH-17**

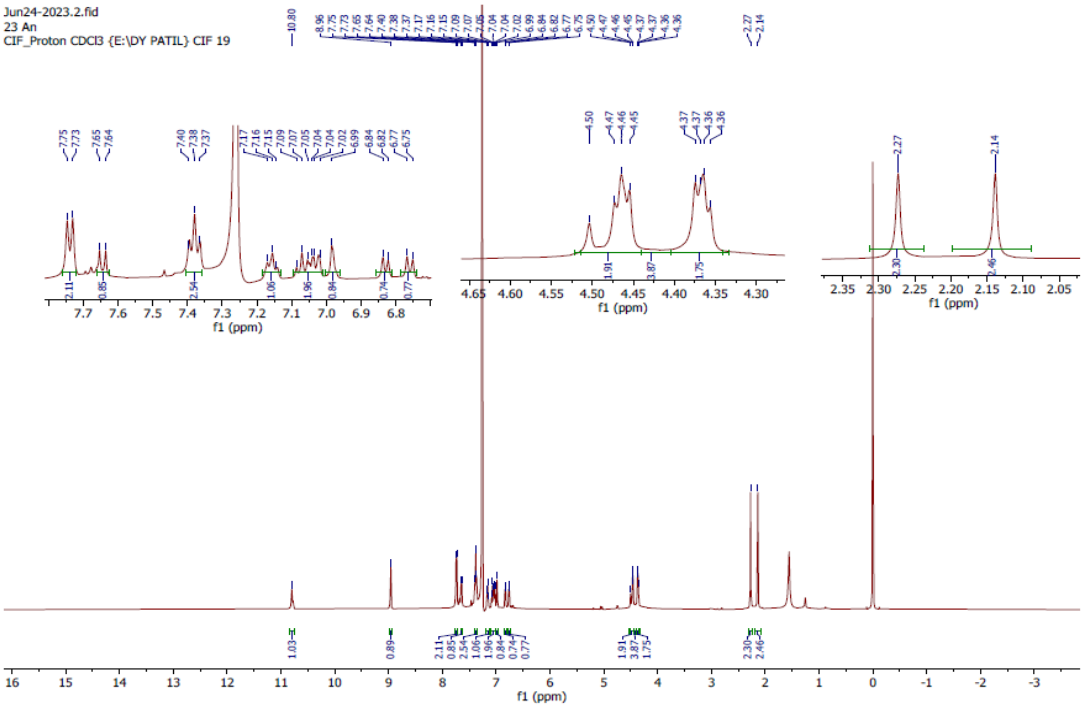

Figure S34. ^{13}C NMR spectra of **RSH-17**

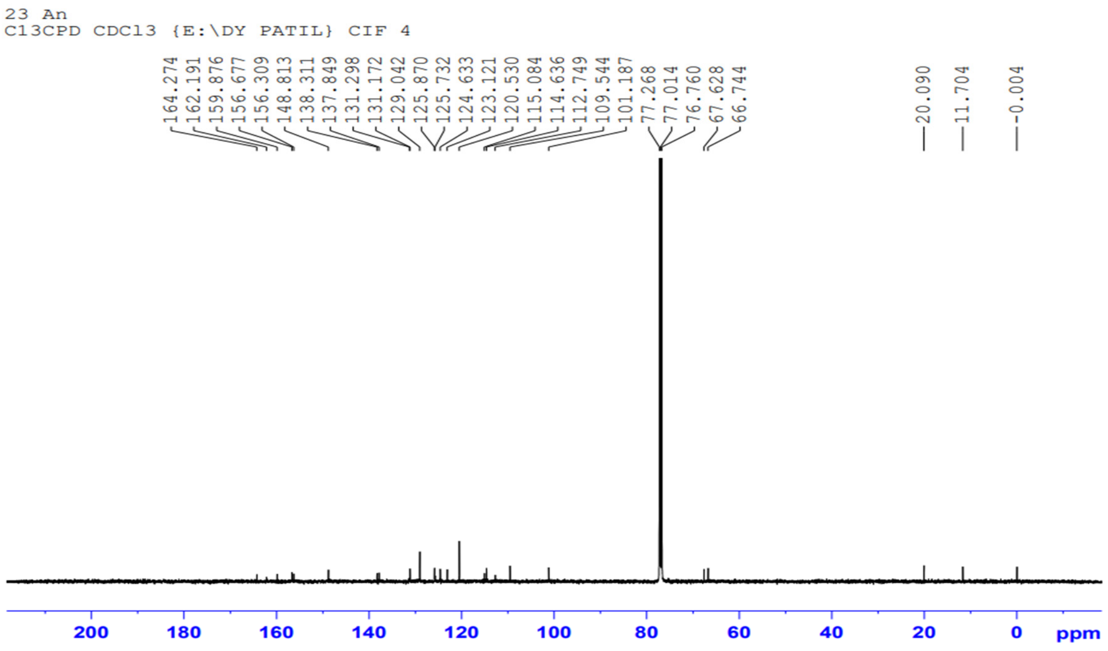

Figure S35. HR MS spectra of **RSH-17**

Acquisition Parameter

Source Type	ESI	Ion Polarity	Positive	Set Nebulizer	1.2 Bar		
Focus	Active	Set Capillary	4500 V	Set Dry Heater	200 °C		
Scan Begin	20 m/z	Set End Plate Offset	-500 V	Set Dry Gas	7.0 l/min		
Scan End	1200 m/z	Set Charging Voltage	2000 V	Set Divert Valve	Source		
		Set Corona	0 nA	Set APCI Heater	0 °C		

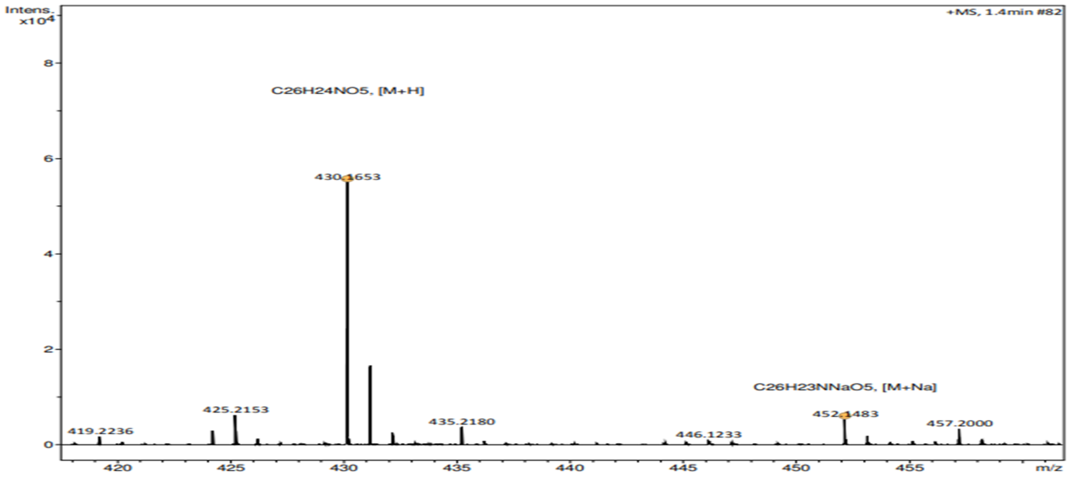

Meas. m/z	#	Ion Formula	Score	m/z	err [mDa]	err [ppm]	mSigma	rdb	e⁻ Conf	N-Rule	Adduct
430.165313	1	C26H24NO5	100.00	430.164899	-0.4	-1.0	7.6	15.5	even	ok	M+H
452.148275	1	C26H23NNaO5	100.00	452.146844	-1.4	-3.2	46.4	15.5	even	ok	M+Na

Figure S36. ^{1}H NMR spectra of **RSH-18**

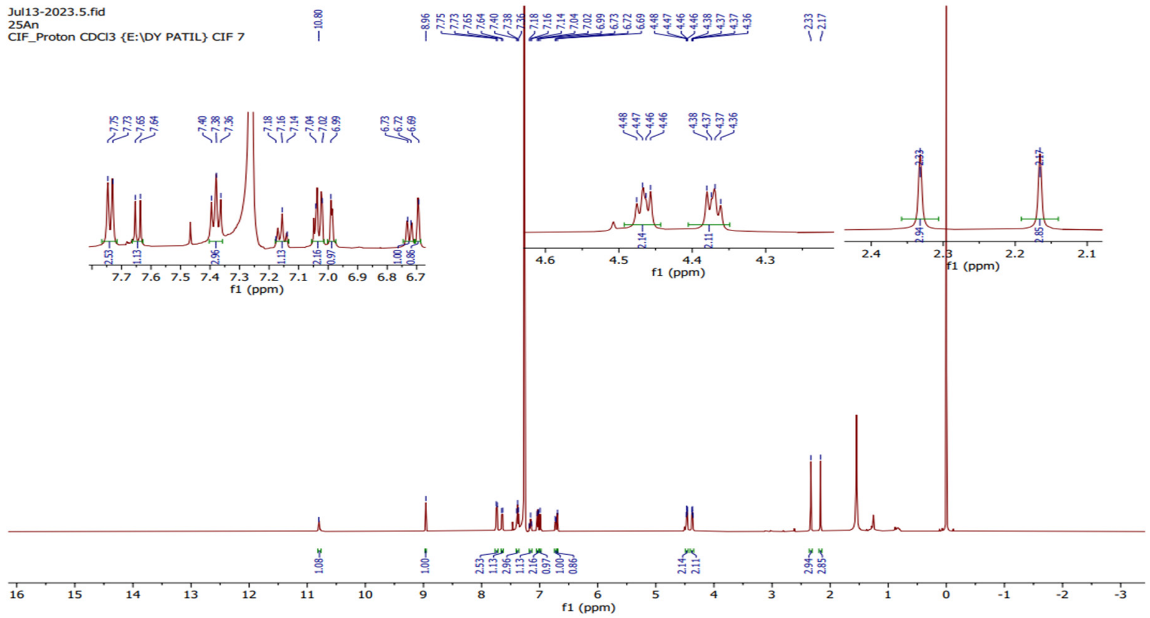

Figure S37. ¹H NMR spectra of **RSH-19**

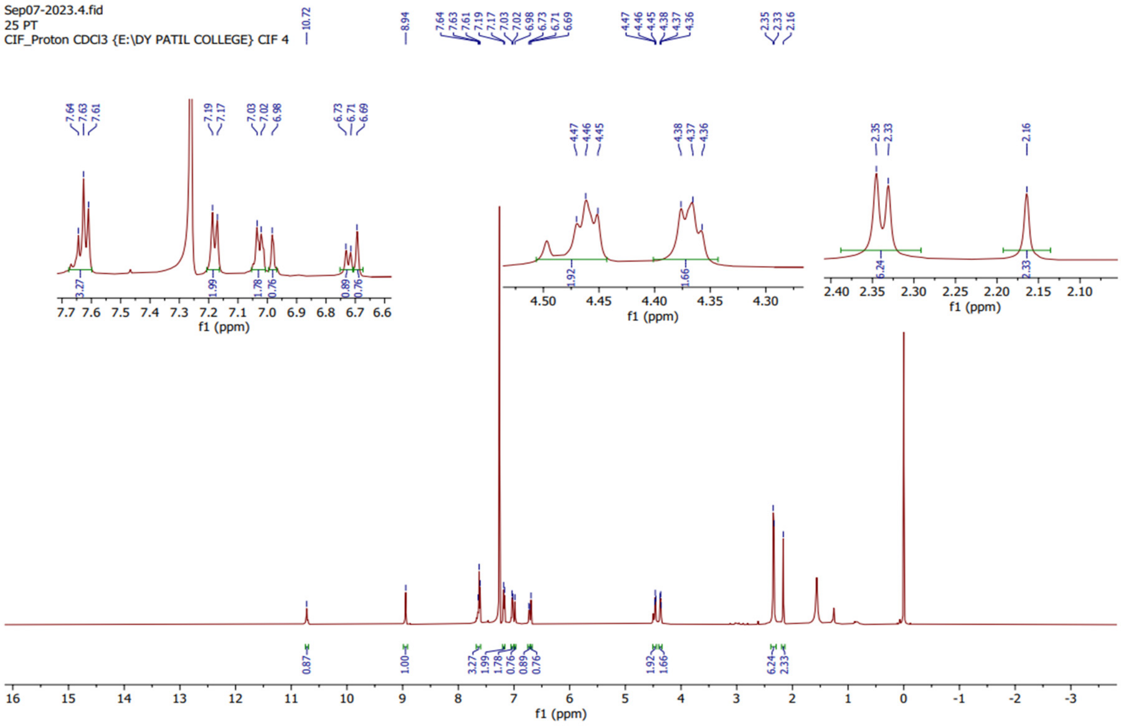

Figure S38. ^{1}H NMR spectra of **RSH-21**

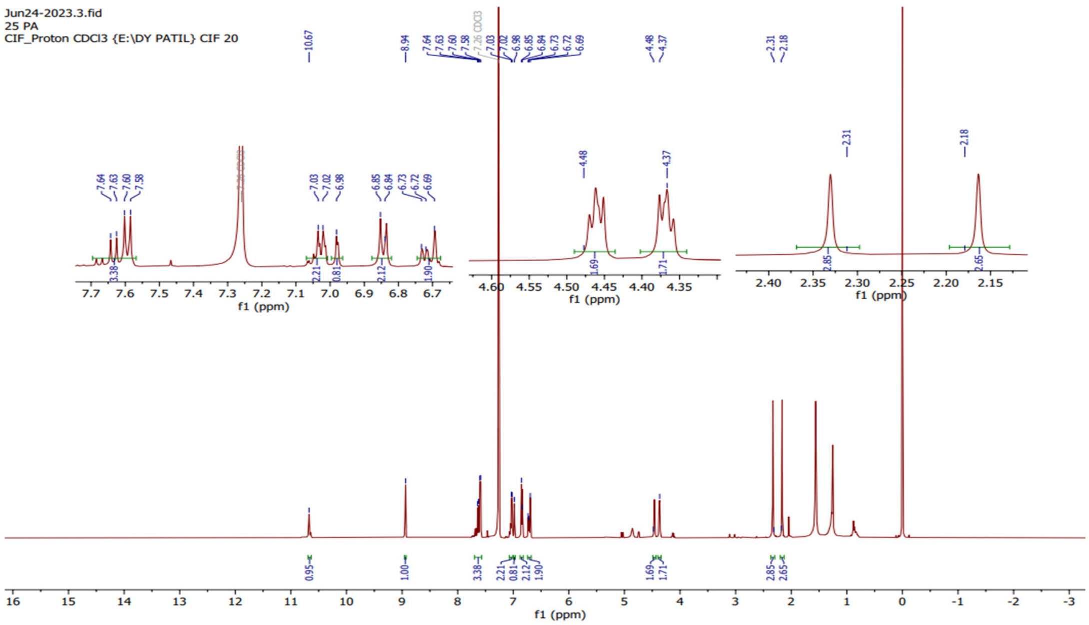

Figure S39. HR MS spectra of **RSH-21**

Acquisition Parameter

Source Type	ESI	Ion Polarity	Positive	Set Nebulizer	1.2 Bar
Focus	Active	Set Capillary	4500 V	Set Dry Heater	200 °C
Scan Begin	20 m/z	Set End Plate Offset	-500 V	Set Dry Gas	7.0 l/min
Scan End	1200 m/z	Set Charging Voltage	2000 V	Set Divert Valve	Source
		Set Corona	0 nA	Set APCI Heater	0 °C

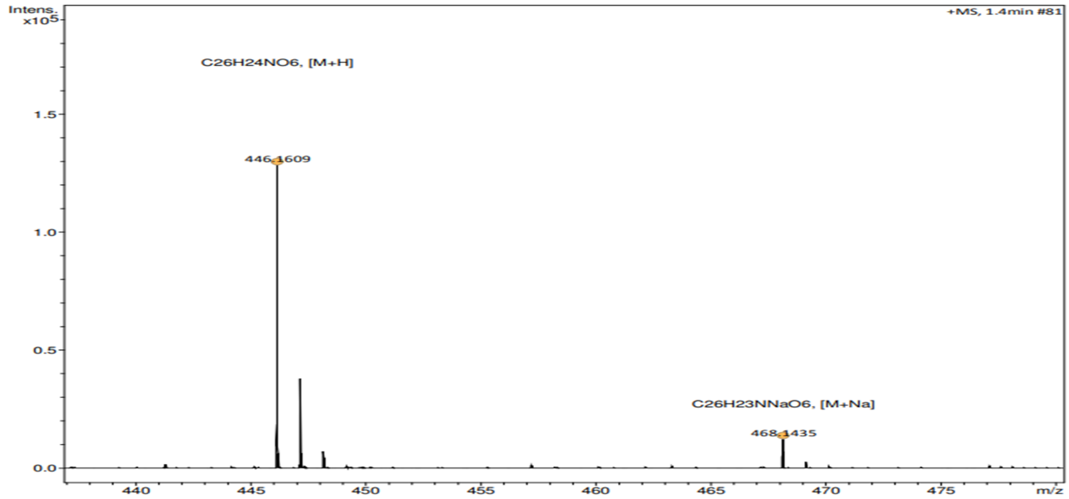

Meas. m/z	#	Ion Formula	Score	m/z	err [mDa]	err [ppm]	mSigma	rdb	e⁻ Conf	N-Rule	Adduct
446.160925	1	C26H24NO6	100.00	446.159814	-1.1	-2.5	3.6	15.5	even	ok	M+H
468.143476	1	C26H23NNaO6	100.00	468.141758	-1.7	-3.7	28.9	15.5	even	ok	M+Na

Figure S40. ^{1}H NMR spectra of **RSH-24**

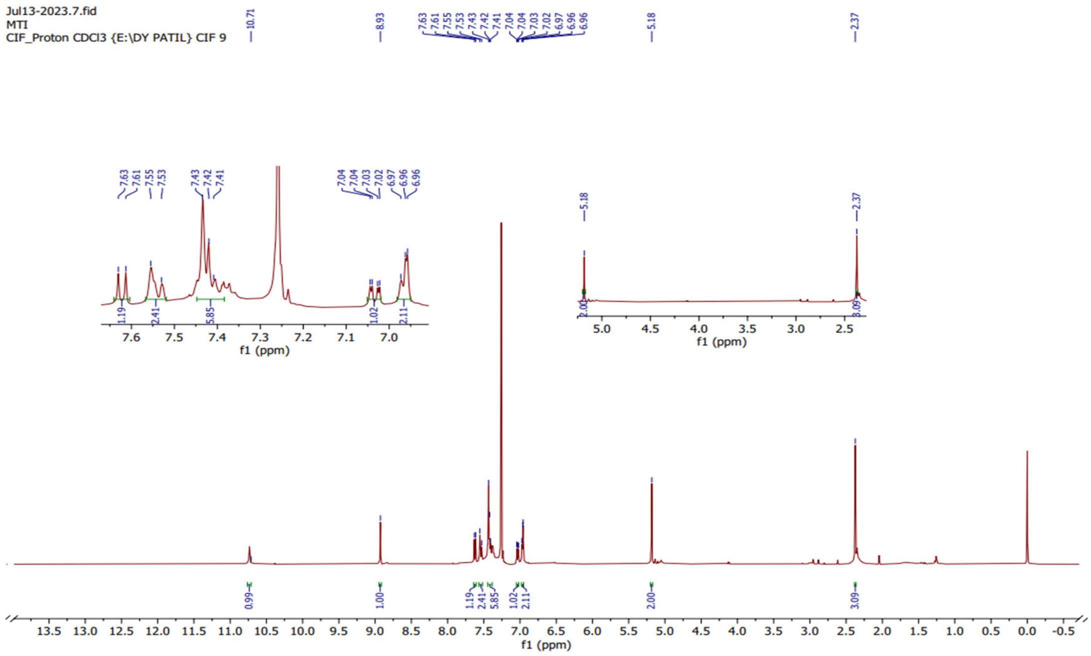

Figure S41. ^{1}H NMR spectra of **RSH-26**

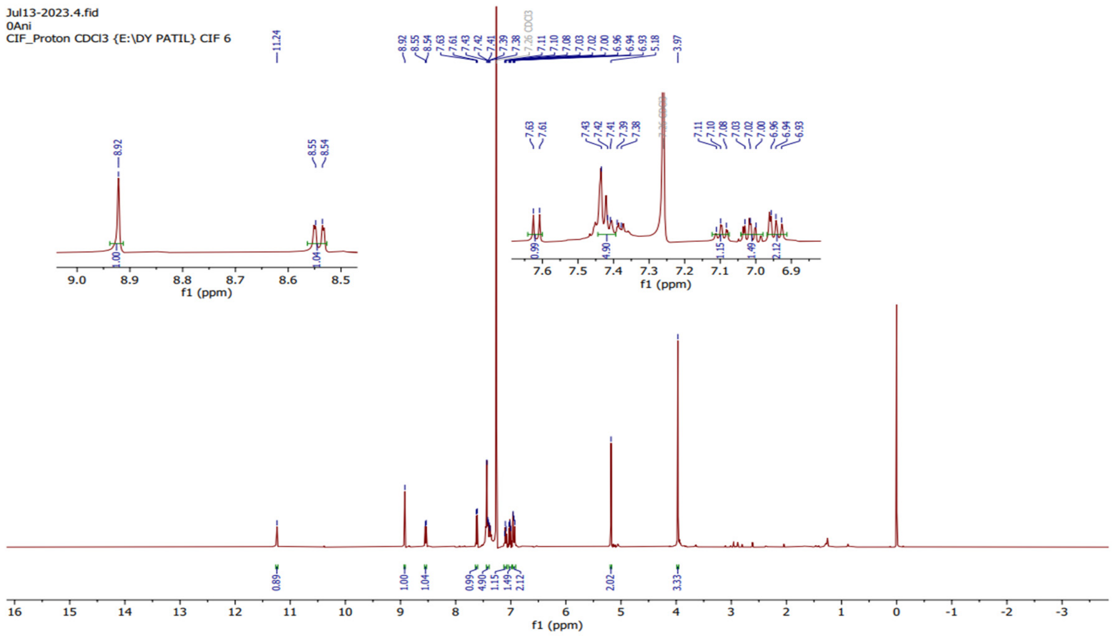

Figure S42. ^{1}H NMR spectra of **RSH-27**

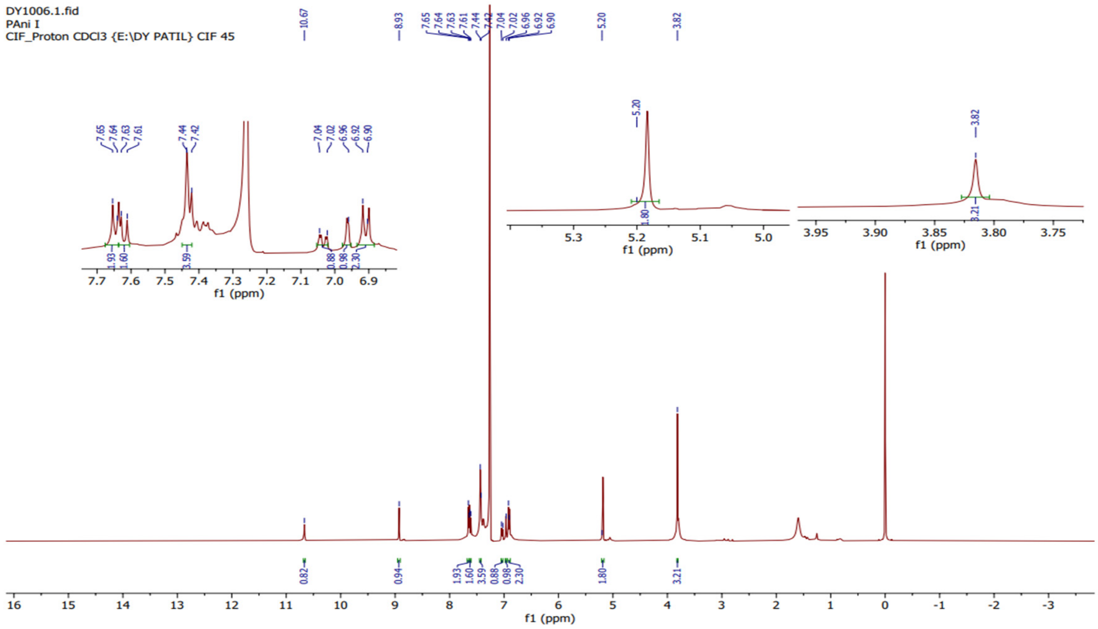

Figure S43. ^{1}H NMR spectra of **RSH-28**

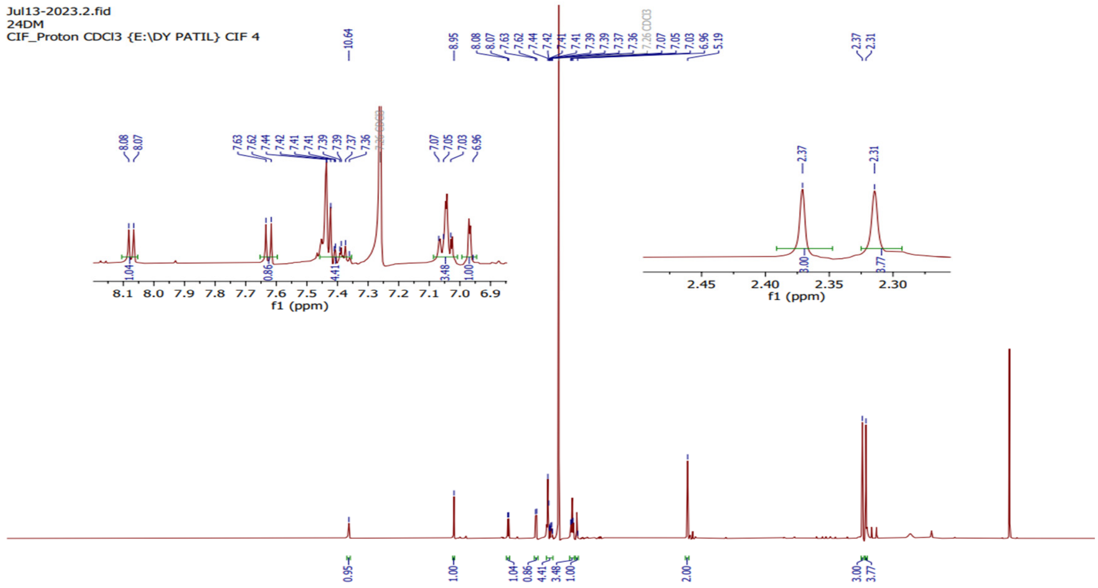

Figure S44. ^{13}C NMR spectra of **RSH-28**

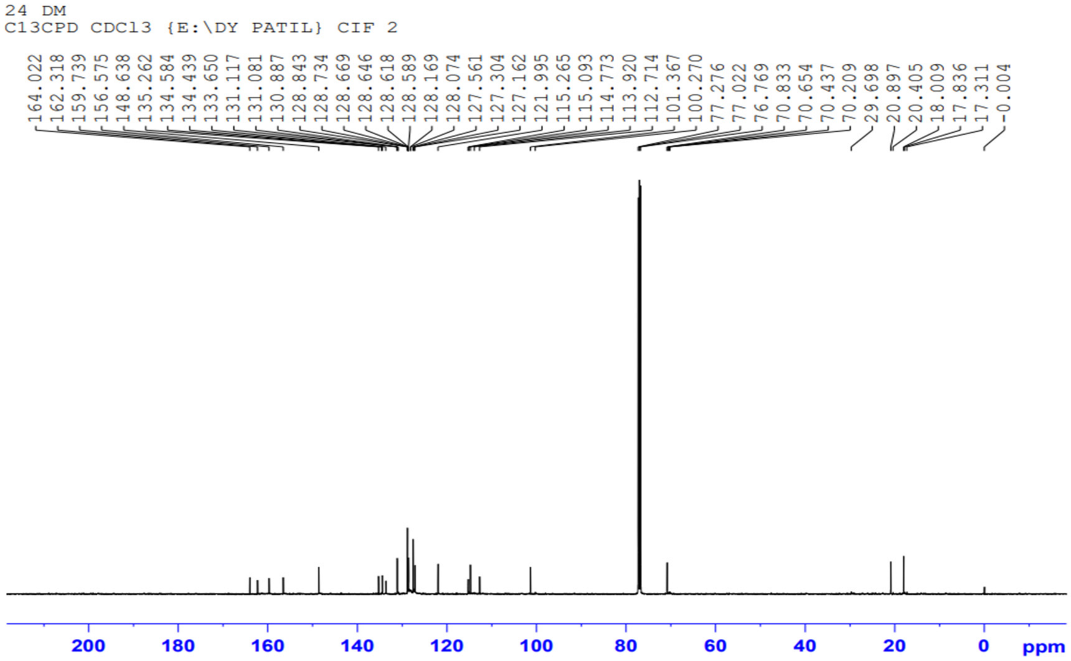

Figure S45. HR MS spectra of **RSH-28**

Acquisition Parameter

Source Type	ESI	Ion Polarity	Positive	Set Nebulizer	0.8 Bar
Focus	Active	Set Capillary	4500 V	Set Dry Heater	200 °C
Scan Begin	20 m/z	Set End Plate Offset	-500 V	Set Dry Gas	7.0 l/min
Scan End	1200 m/z	Set Charging Voltage	2000 V	Set Divert Valve	Source
		Set Corona	0 nA	Set APCI Heater	0 °C

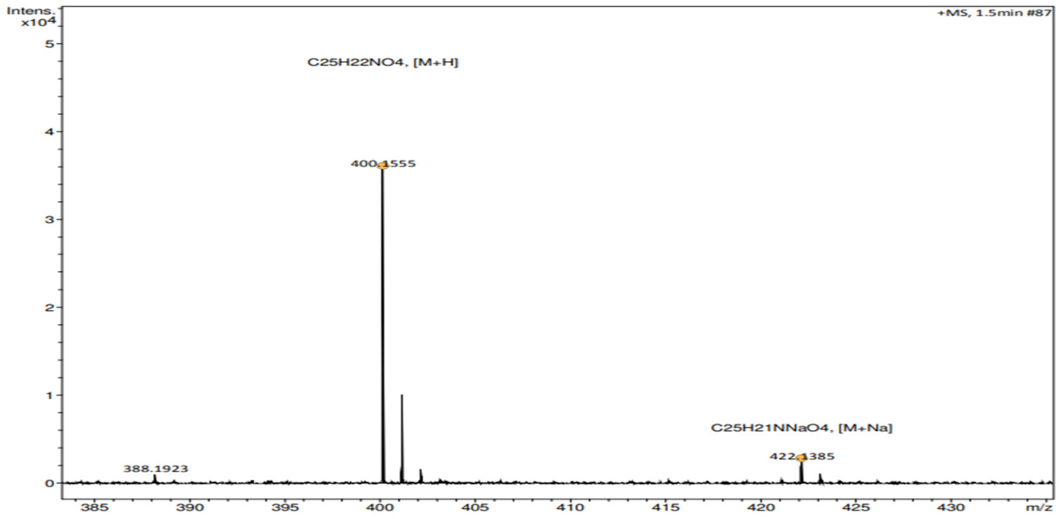

Meas. m/z	#	Ion Formula	Score	m/z	err [mDa]	err [ppm]	mSigma	rdb	e⁻ Conf	N-Rule	Adduct
400.155471	1	C25H22NO4	100.00	400.154335	-1.1	-2.8	2.3	15.5	even	ok	M+H
422.138511	1	C25H21NNaO4	100.00	422.136279	-2.2	-5.3	92.6	15.5	even	ok	M+Na

Figure S46. ¹H NMR spectra of **RSH-29**

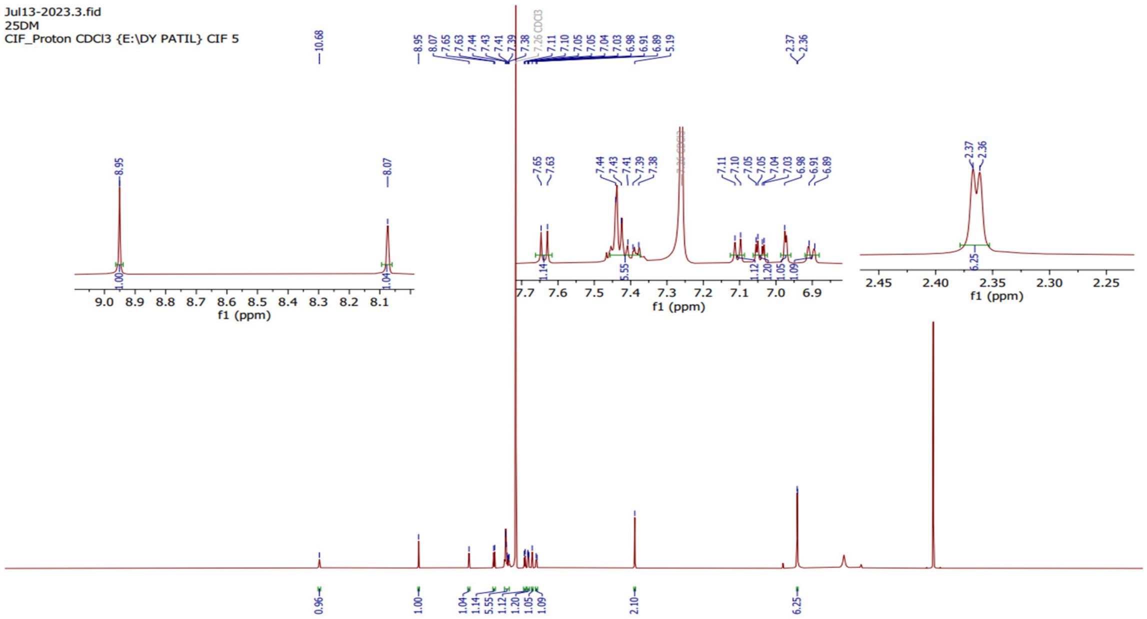

Figure S47. ^{1}H NMR spectra of **RSH-30**

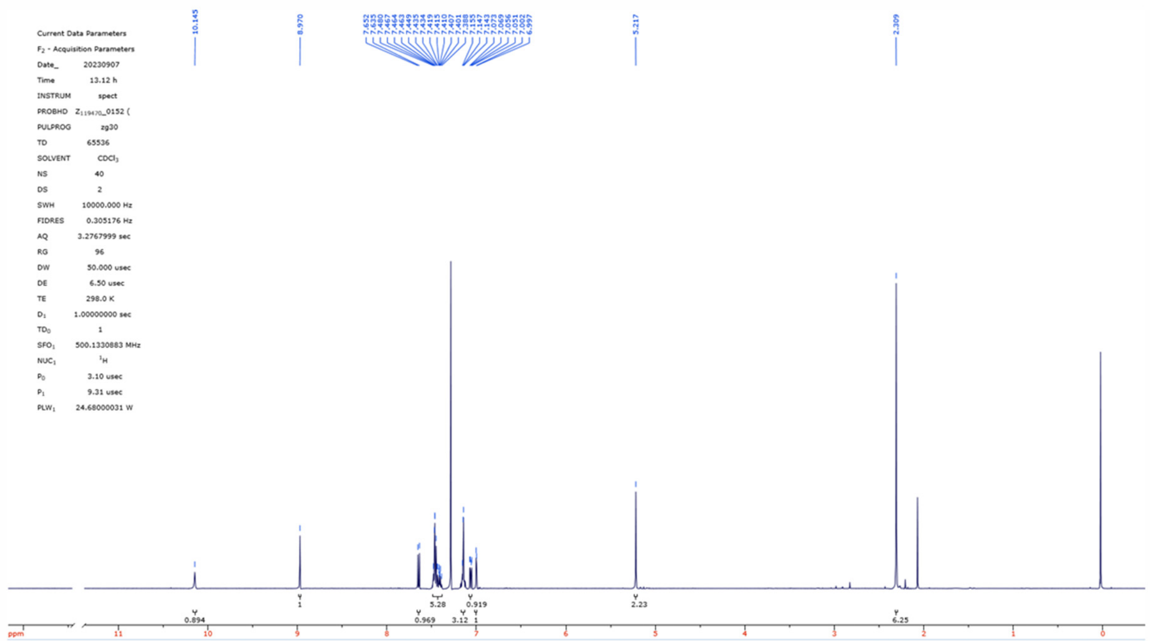

Figure S48. ^{1}H NMR spectra of **RSH 31**

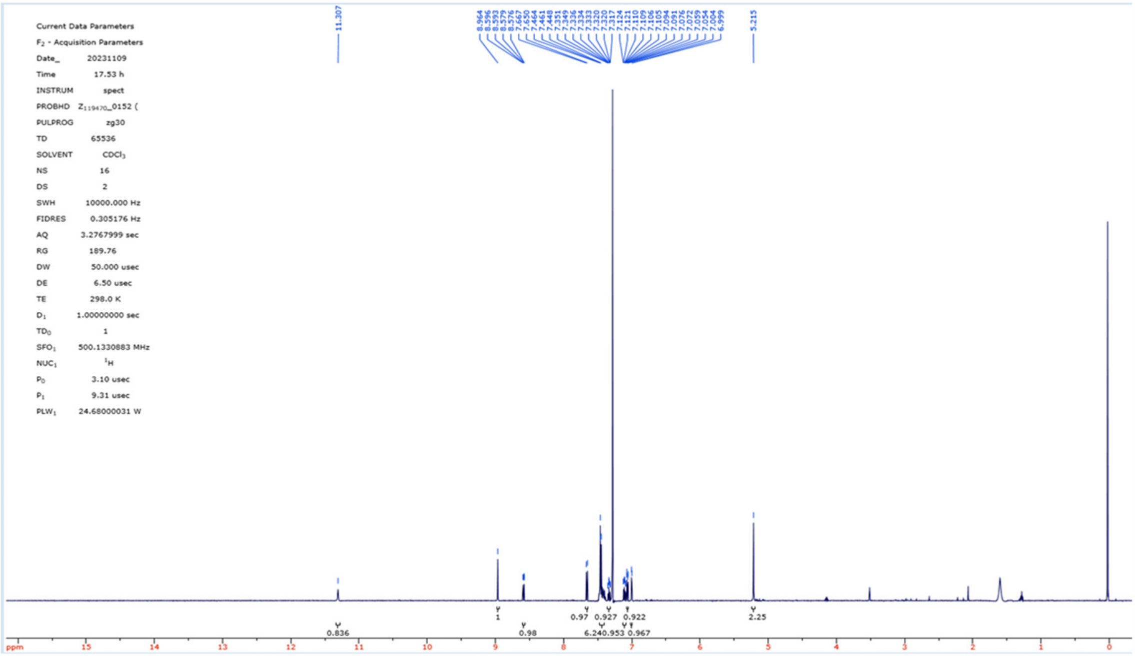

Figure S49: Western Blot images.

No-stain whole protein labeling following gel transfer (related to Fig. 8A)

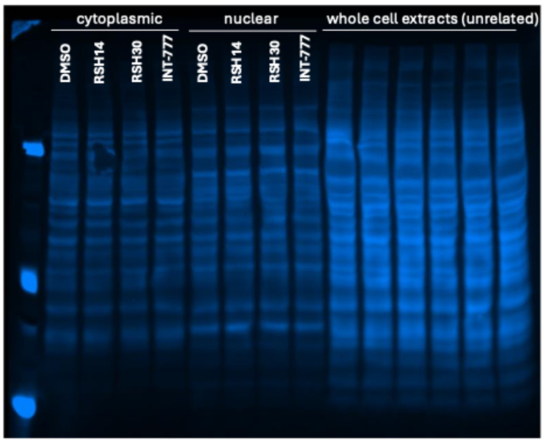

Western blot for ATF6α (related to Fig. 8A)

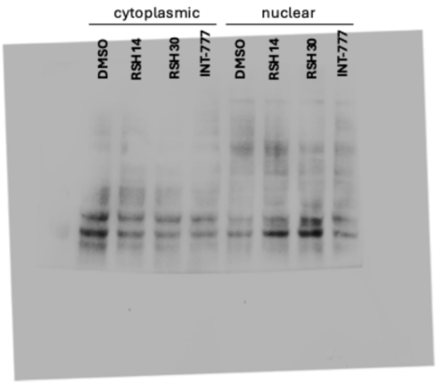

Western blot for histone (related to Fig. 8A)

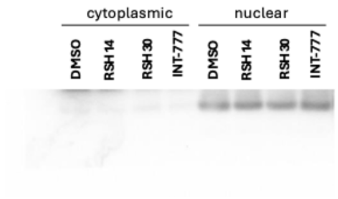

Supplement: Supplementary file 1 [file molecules-31-01093-s001.zip › molecules-4188258-supplementary.pdf]
